# Computational Investigation on the Origin of Atroposelectivity for the Cinchona Alkaloid Primary Amine-Catalyzed Vinylogous Desymmetrization of *N*-(2-*t*-Butylphenyl)maleimides

**DOI:** 10.1021/acs.joc.1c01235

**Published:** 2021-08-04

**Authors:** Nicolò Tampellini, Paolo Righi, Giorgio Bencivenni

**Affiliations:** Department of Industrial Chemistry “Toso Montanari”, Alma Mater Studiorum University of Bologna, Viale del Risorgimento 4, 40136 Bologna, Italy

## Abstract

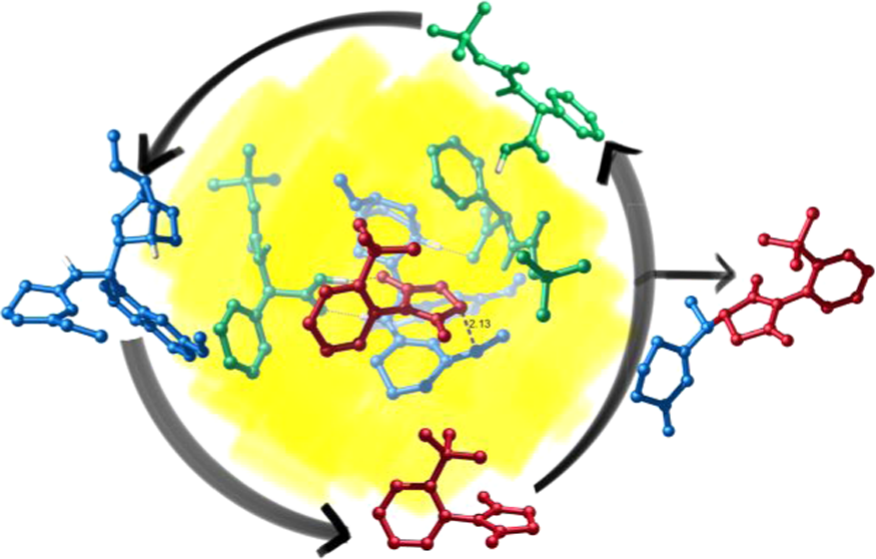

Mechanistic studies clarifying how chiral primary amines control the stereochemistry of vinylogous processes are rare. We report a density functional theory (DFT) computational study for the comprehension of the reaction mechanism of the vinylogous atroposelective desymmetrization of *N*-(2-*t*-butylaryl)maleimide catalyzed by 9-amino(9-deoxy)epi-quinine. Our results illustrate how the origin of the atroposelectivity was realized by the catalyst through steric and dispersion interactions. The role of *N*-Boc-l-Ph-glycine was crucial for the formation of a closed transition-state geometry and the activation of both reaction partners.

## 1 Introduction

**Scheme 1:**
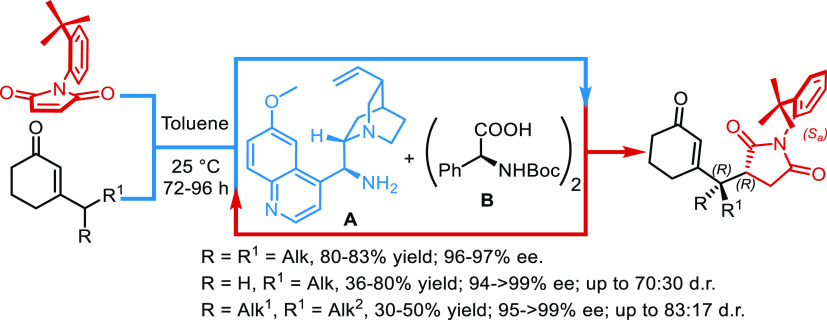
Vinylogous Atroposelective Desymmetrization of *N*-(2-*t*-Butylphenyl)maleimides

The use of organocatalysis for the synthesis of enantioenriched compounds represents a powerful tool in chemists’ hands. In particular, asymmetric aminocatalysis, based on the use of chiral secondary and primary amines, demonstrated its efficiency in the activation of carbonyl compounds.^1^ In this vast scenario, the role played by chiral primary amines derived from natural Cinchona alkaloids is well recognized as fundamental and necessary for the enantioselective functionalization of encumbered linear or cyclic aldehydes and ketones, not only through a canonical iminium ion and enamine activation but also by means of vinylogous reactivity.^2^ As a consequence of the propagation of the electronic properties of functional groups through a conjugated system, the vinylogous activation allows the functionalization of unsaturated carbonyl compounds at a remote position from the catalytic core.^3^ Despite the fact that primary amine catalysts derived from Cinchona alkaloids are efficient aminocatalysts enabling vinylogous reactions, the way the catalytic machinery operates to control the remote stereoselectivity is almost unknown. If we exclude the pioneering work on the γ-amination of linear unsaturated aldehydes by Jørgensen,^4^ nevertheless catalyzed by a proline-derived catalyst, computational investigations have been mainly concentrated on enamine and iminium ion activation ways. To date, the fundamental contributions by Melchiorre, Houk, List, and Higashi furnished solid models for the rational comprehension of the reaction mechanisms of venerable organic reactions.^5^ In 2014, we realized the synthesis of atropisomeric succinimides via vinylogous Michael addition of 3-alkylcyclohexenones to axially prochiral *N*-(2-*t*-butylaryl)maleimide^6^ (Scheme 1). This desymmetrization, a rare application of a vinylogous aminocatalytic reaction to the synthesis of atropisomeric compounds, was efficiently promoted by 9-amino(9-deoxy)epi-quinine (9-ADEQ) **A** as a catalyst in combination with an *N*-Boc-l-Ph-glycine **B** cocatalyst. The experimental results evidenced how the vinylogous addition took place to the side of the maleimide double bond not shielded by the *t*-butyl group. As a result, the contemporary control of two different stereogenic elements was achieved and the desymmetrization path was highly enantio- and diastereoselective when nonprochiral dienamines were employed. The great stability of the stereogenic axis (Δ*G*_epi_^‡^ = 31.9 kcal/mol) allows no epimerization of the C–N single bond, and the observed overall diastereomeric ratio was the result of an epimerization taking place at the exocyclic stereocenter. With the aim to elucidate the reasons for the stereochemical outcome observed and to provide a general mechanistic model, we report, herein, our results of the computational studies using density functional theory (DFT) methods on the vinylogous desymmetrization of proatropochiral maleimides catalyzed by 9-ADEQ.

## 2 Results and Discussion

First, we tried to estimate the activation energy for the inversion of the stereogenic axis configuration of **3a**. We found two transition states (**TS**) **3a-TS1** and **3a-TS2**, one for each direction in which one ring can be rotated about the other (Table 1). To obtain a better Δ*G*^‡^ estimate, a quasi-harmonic (qh) frequency analysis of these structures was carried out with the GoodVibes program.^7^ The obtained results are in very good agreement with the experimentally observed value of 31.9 kcal/mol.^6^

**Table 1:**
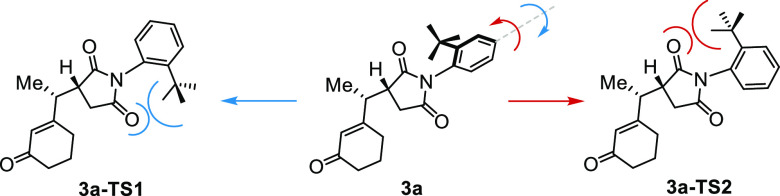
TS Energies for the Inversion of the Stereogenic Axis Configuration of 3a^a^

name	rel. EE^b^	rel. qh-H^c^	rel. qh-G^d^
3a	+0.00	+0.00	+0.00
3a-TS1	+33.31	+33.15	+35.35
3a-TS2	+33.53	+33.29	+35.42

a TS energies are reported in kcal/mol at the ωB97X-D/6-311G(d,p)-conductor-like polarizable continuum model (CPCM) (toluene) level of theory.

b Relative electronic energy (Gaussian).

c Relative enthalpy (Gaussian and GoodVibes).

d Relative Gibbs free energy (Gaussian and GoodVibes).

**Scheme 2:**
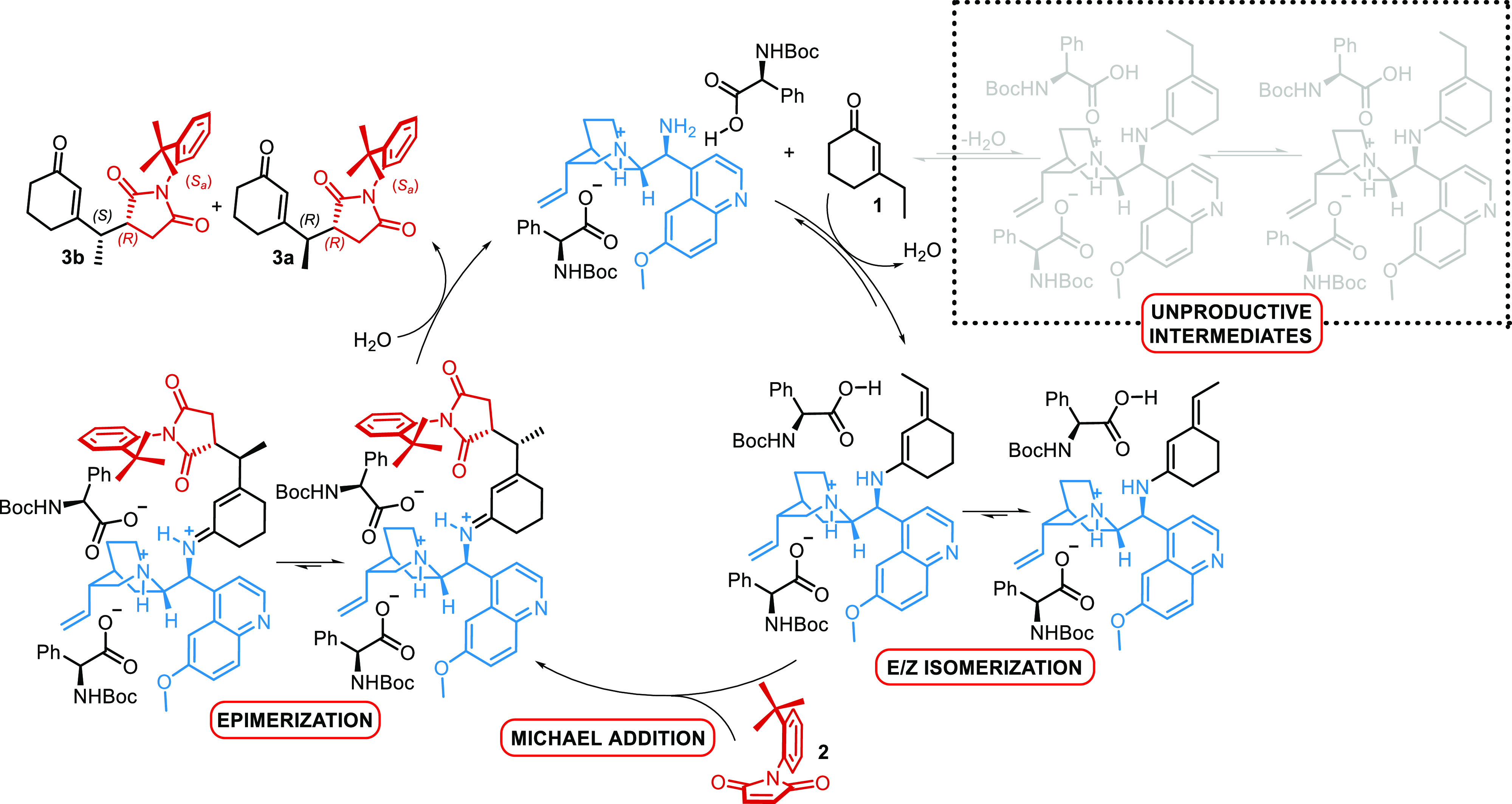
Catalytic Cycle for the Vinylogous Atroposelective Desymmetrization of *N*-(2-*t*-Butylphenyl)maleimide

Then, as a model for the computational study, we choose the reaction between 3-ethyl-cyclohex-2-en-1-one **1** and *N*-(2-*t*-butylphenyl)maleimide **2**, which was conducted using 20 mol % chiral amine **A** and 40 mol % *N*-Boc-l-Ph-glycine **B** in toluene at room temperature. Since the very beginning of the experimental work, we observed that the amount and the nature of the acidic cocatalyst strongly impacted the yield of the process, but not the enantioselectivity.^8^ Initial experimental results showed that an aminocatalyst:acidic cocatalyst ratio of 1:2 afforded the best yield, so we used that ratio throughout the subsequent development of the experimental work. All tested acids gave very high levels of enantiocontrol, underlying that 9-ADEQ had a key role in the enantioselectivity. Therefore, in agreement with the experimental protocol, in this computational study, we included two molecules of the acidic cocatalyst in **TS** models we studied. Under these conditions, we were able to obtain **TS** models that match the experimental results in terms of the enantioselectivity of the process, the absolute configuration of the products, and the diastereoselectivity. In addition, we could not find any reasonable **TS** model when we tried to include only one molecule of the acidic cocatalyst. We anticipated that in the **TS** models we found, one molecule of the acidic cocatalyst protonates the quinuclidine nitrogen of **A**, while the second activates both the nucleophile and electrophile of the reaction. A catalytic cycle for the desymmetrization is proposed (Scheme 2). After the condensation of catalyst **A** to ketone **1**, an equilibrium mixture of four intermediates is established. *E*- and *Z*-exocylic dienamines react with maleimide **2**, leading to a mixture of diastereoisomeric iminium ions that release the observed mixture of diastereoisomers **3a** and **3b** after hydrolysis.

**Scheme 3:**
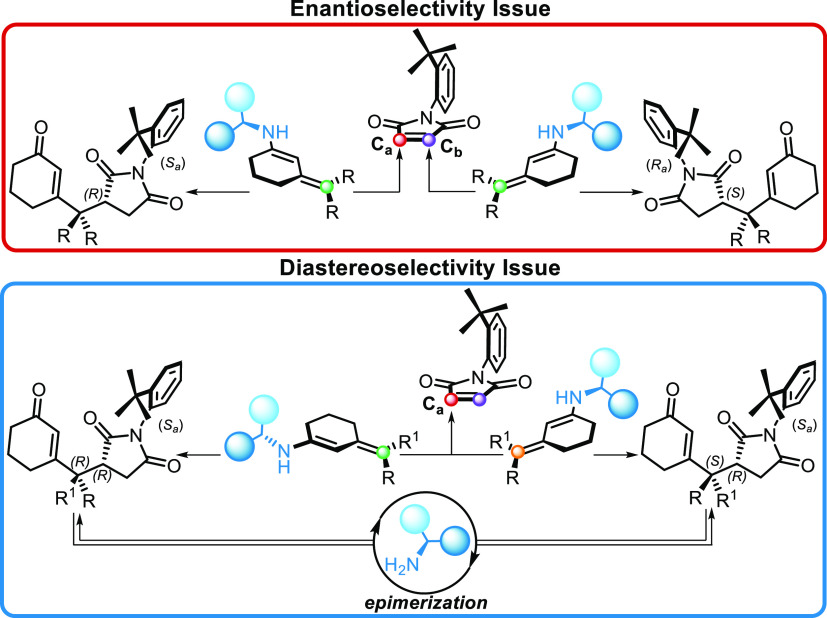
Enantio- and Diastereoselectivity for the Vinylogous Michael Addition

As we know, the final ratio between **3a** and **3b** is the result of catalyst-mediated epimerization.^9^ Because of the particular structure of maleimide **2**, wherein the *t*-butyl group shields the upper side of the double bond, the only plausible approach of the nucleophile can be from the bottom, where the Si face of *C*_a_ and the Re face of *C*_b_ are exposed. The exclusive addition of a vinylogous nucleophile (prochiral or not) to one of the two carbon atoms is selective for the resulting stereogenic axis and endocyclic stereocenter. Addition to the *C*_a_ gives the (*R*,*S*_a_)-product and the addition to *C*_b_ gives the enantiomeric (*S*,*R*_a_)-product. So, the origin of the asymmetric induction must be found in the reason why catalyst **A** directs the Michael addition of the dienamine preferentially to one carbon atom rather than the other one (Scheme 3, red circle). Instead, once considered the *t*-butyl shielding effect as a principal factor of diastereoselectivity, relative to the endocyclic stereocenter, when prochiral vinylogous nucleophiles are employed, the configuration at the exocyclic carbon atom depends on the relative approach between the prochiral faces of dienamines and maleimide (Scheme 3, blue circle). Despite knowing that a catalyst-promoted epimerization leads to the observed diastereoselectivity, a detailed study of the Michael addition is required to elucidate catalyst performance in controlling the relative configuration at the remote position.

### 2.1 Dienamine and Catalyst Conformation Analysis

Computational investigations started from the simplest iteration of the reaction described in our previous work, represented in Scheme 2. Initial efforts were focused on investigating intermediate species in the reaction mechanism. Dienamines arising from condensation between cyclohexenones **1** and the catalyst 9-ADEQ were the first to be studied.

**Figure 1:**
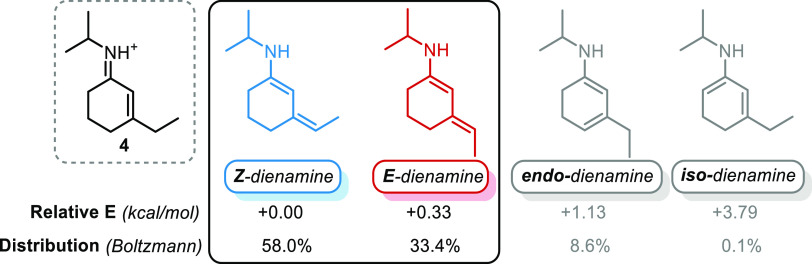
Relative energy for the most stable dienamine conformers at the ωB97X-D/6-311G(d,p)-CPCM (toluene) level of theory.

To address the energetic accessibility of different isomers, an initial investigation of their energy was carried. To isolate the contribution of double-bond disposition and reduce the overall complexity, the primary amine catalyst was substituted with isopropylamine in these calculations. Four dienamines could potentially arise from iminium ion **4** conformational analyses and DFT optimization of conformers led to the equilibrium geometry for each structure (Figure 1).

**Scheme 4:**
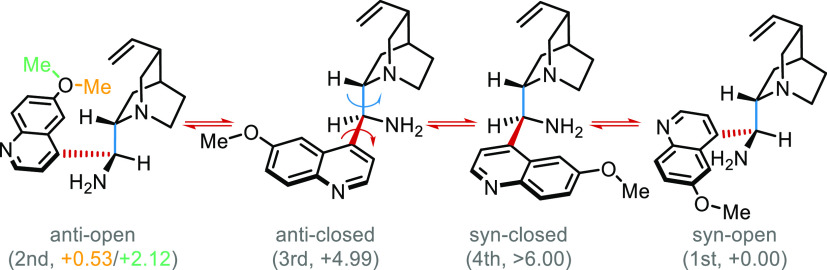
Relative Energy for the Most Stable Catalyst Conformers at the ωB97X-D/6-311G(d,p)-CPCM (Toluene) Level of Theory

Results show that *E*- and *Z*-dienamines are dominant in solution, while *endo*-dienamine is only remotely accessible. The presence of *iso*-dienamine in solution appears negligible. Transition states (**TS**) for the four possible γ-deprotonation reactions of iminium ion **4** were sought with various techniques, but no energetic maximum was found for any of them. We conclude that there is no evident barrier for the formation of any of these dienamines, and their distribution is dictated by thermodynamics. Therefore, since both *E-* and *Z-* isomers are very similar in energy, the dienamine population ratio is likely to be excluded from the factors shifting product distribution. Next, we performed a conformational analysis and DFT optimization on catalyst **A** and found results coherent with previous works.^5a,10^ Catalyst **A** adopts two main conformations, namely, ***syn*-open** and ***anti*-open**. The ***syn*-** or ***anti*-** labels are assigned on the spatial relation of the amino and methoxy groups, while open or closed are based on amine nitrogen and quinuclidine nitrogen disposition. Results of conformational search and DFT optimizations are summarized in Scheme 4. Most stable catalyst conformers adopt an open geometry, most likely due to stabilization by an intramolecular hydrogen bond between the amino and the quinuclidine groups.

**Scheme 5:**
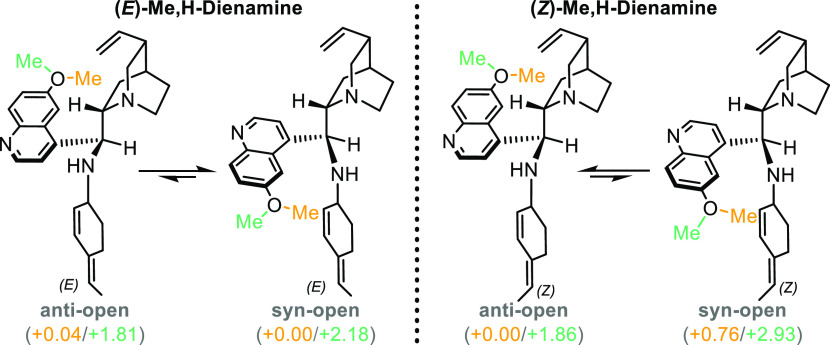
Relative Energy for the Most Stable *E*- and *Z*-Dienamine Conformers at the ωB97X-D/6-311G(d,p)-CPCM (Toluene) Level of Theory

The same conformational analysis is then extended to complete catalyst-derived *E*- and *Z*-dienamines. Similar results are obtained, favoring closed conformations over open ones by 3–5 kcal/mol. Quinoline ***syn*-** or ***anti*-**conformations on the other hand are very close in energy and are similarly populated (Scheme 5).

### 2.2 Transition-State Modeling

**Figure 2:**
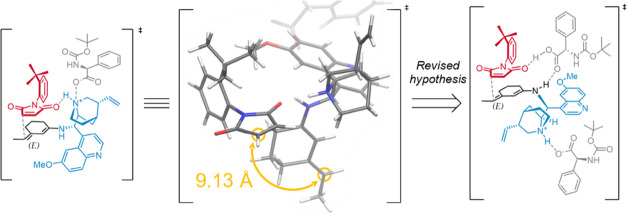
First hypothesis of the transition-state model (left), the 3D structure showing the reactive atoms distance in the prereaction complex (center), and the updated model (right).

Starting from the conformations obtained, we modeled the **TS** as it was proposed in the original experimental paper. In this initial model, the main secondary interaction, which we believed to be responsible for the efficient atroposelection observed, lied in a strong hydrogen bond between the charged quinuclidinium nitrogen and the maleimide carbamoyl oxygen. We soon realized that this disposition, although rational, was not spatially apt for the reaction. The equilibrium geometry for the prereaction complex showed a distance between reacting carbon atoms greater than 9 Å. Moreover, the system tended to pair the ions: the protected glycine carboxylate was strongly attracted to the quinuclidinium charged site, replacing the maleimide in its activating interaction (Figure 2).

**Scheme 6:**
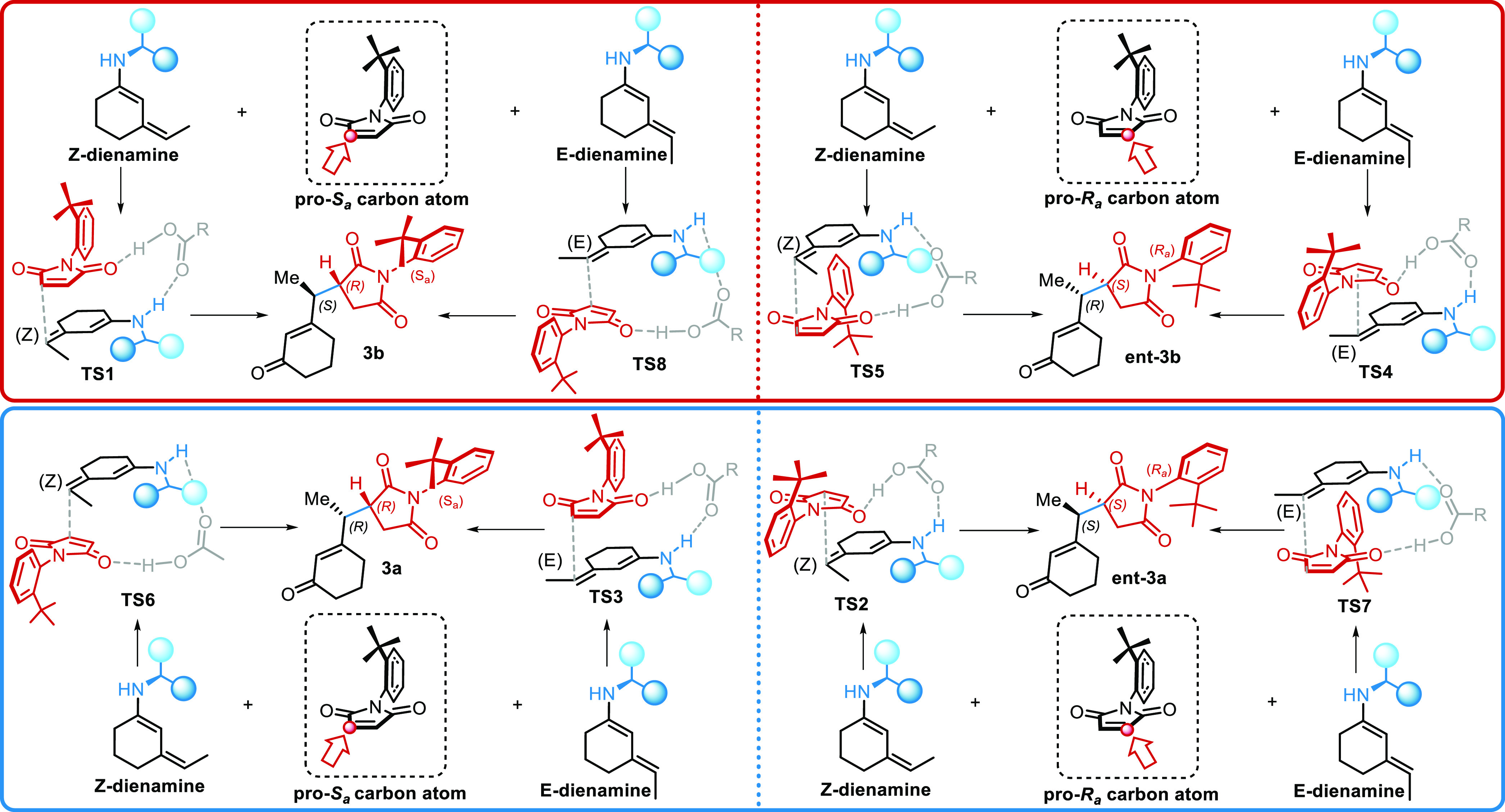
Transition-State Arrangements

With these results in hand, the first **TS** model was questioned. Speculations were made on whether the second molecule of the acidic cocatalyst might play a determining role in this **TS**. A hypothesis was put forward, where the first molecule protonates the quinuclidine moiety of the catalyst forming a salt, while the second bridges and activates the reacting partners in a cyclical fashion. From this new model, we identified four binary degrees of freedom for the construction of a **TS**, which could explain the enantio- and diastereoselectivity: maleimide β-carbon atom, maleimide face, dienamine face, and dienamine exocyclic double-bond configuration. Moreover, another binary possibility is given by the quinoline portion, which showed to be energetically accessible either in the ***syn*-** or ***anti*-**conformation. While these considerations would lead to seeking a total of 32 transition-state arrangements, only a minor number of them are presented (Scheme 6). In fact, the maleimide face degree of freedom is removed by the consideration that the *t*-butyl group shields one maleimide face completely. The remaining 16 **TSs** are numbered 1 to 8, with a **-*syn*** or **-*anti*** suffix to indicate quinoline conformation. When trying to obtain even-numbered **TSs** (**TS2**, **TS4**, **TS6**, and **TS8**), we noticed that unlike the odd ones, they did not allow the bridging interaction with the acid. Therefore, odd numbered “**endo-**” **TSs** were maintained, while even-numbered “**exo**-” ones were discarded.

**Scheme 7:**
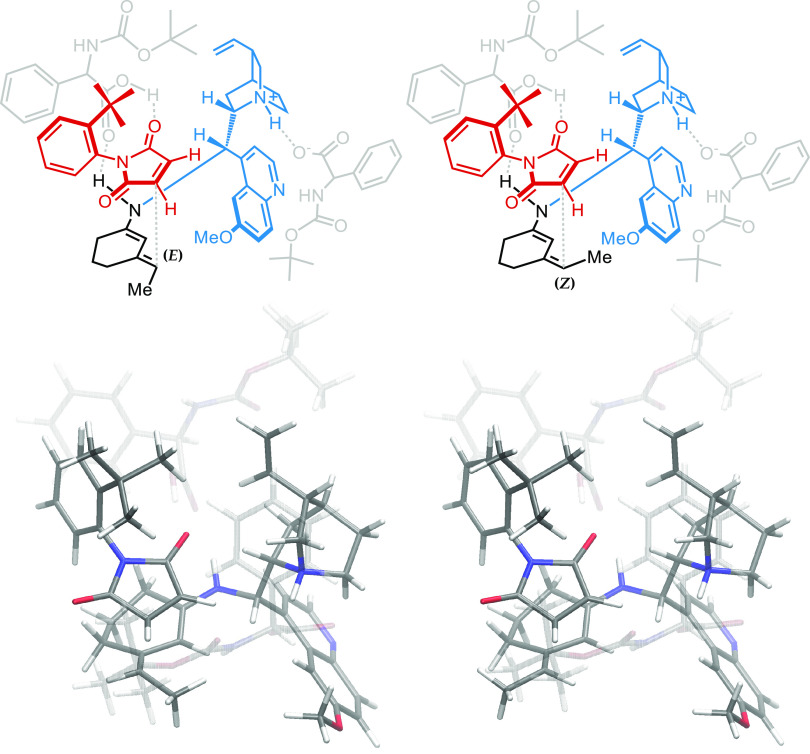
Transition-State Geometry Overview: Two Equivalents of Acid Are Present; the Second One Activates the Reacting Partners in a Cyclical Fashion

After a thorough exploration of computational strategies, we finally obtained the desired transition states (Scheme 7).^11^

**Figure 3:**
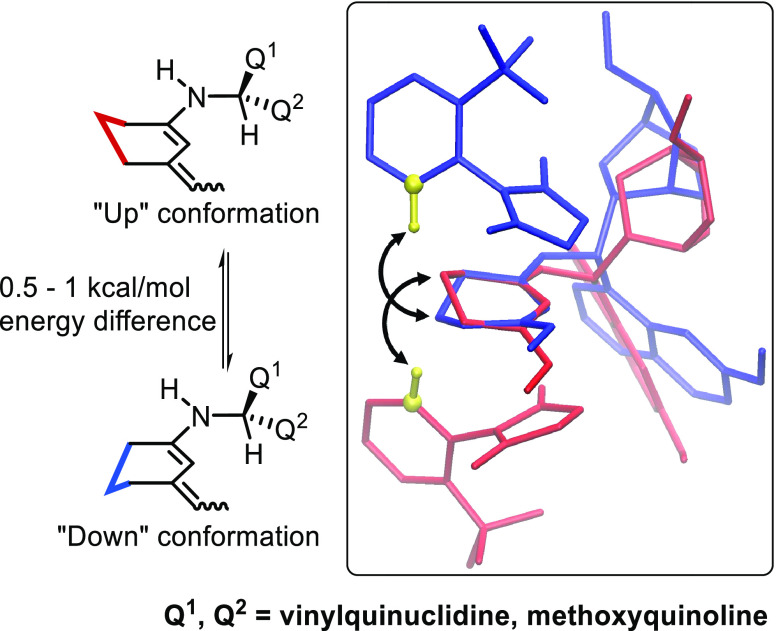
Superposition of fragments of **TS3-*anti*** (blue) and **TS7-*anti*** (red), aligned on a dienamine π system. The dienamine half-chair conformation must avoid clashing between phenyl C–H (yellow) and dienamine-saturated fragment (arrows).

Cyclical transition states were obtained starting from one of two similar dienamine conformers, which differ for the cyclohexene ring conformation and can be classified as half-chairs (Figure 3). Transition states were composed using the conformer that avoided clashing between the maleimide phenyl ring C–H bond and the out-of-plane half-chair fragment. The energetic difference between these half-chair conformers is small, and never exceeds 1 kcal/mol.

**Figure 4:**
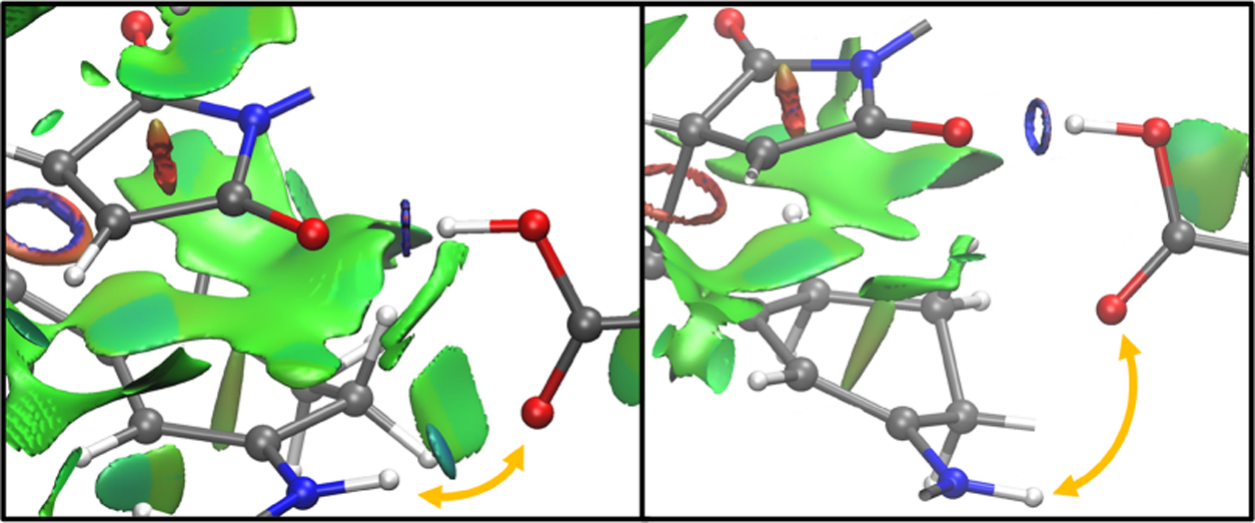
NCIPLOT image of noncovalent interactions around the bridging interaction site. Colored volumes ranging from red to green to blue indicate regions of space where the interactions are repulsive, neutral, and attractive, respectively.

The energy cost of adopting a higher energy conformation is abundantly compensated by the energy gain of obtaining cyclical transition states. Indeed, a great energy difference is present between cyclical **TSs** and noncyclical analogues we obtained while pursuing the former. One specific analogue of **TS1-*****anti***, built from the wrong “up” conformation of the dienamine cyclohexene half-chair, could not achieve an ideal bridging interaction with the amino acid catalyst. Indeed, the resulting **TS** had a CO–NH hydrogen bonding distance of 2.52 Å. We could not achieve proper continuity in any way other than by inverting the half-chair fragment conformation, accessing **TS1-*anti*** structure. This novel geometry, with a CO–NH hydrogen bonding distance of 1.90 Å, was 4.98 kcal/mol lower in energy (ωB97X-D/6-311G(d,p) level of theory). The same process of half-chair inversion on a noncyclical analogue afforded **TS3-*anti***, 7.12 kcal/mol more stable than its previous analogue. We used NCIPLOT^12^ to confirm the presence of the hypothesized CO–NH interaction by visualizing the noncovalent interaction volumes. The settings we used were all default ones, with a FINE integration grid specification. The program confirmed that this hydrogen bond is indeed present and has a stabilizing character, as indicated by the green-blue color of the interaction disc (Figure 4).

### 2.3 Role of the Acidic Cocatalyst

Two equivalents of acid are present in the transition-state model (Scheme 7). The first one reacts with a quinuclidinic nitrogen atom of the catalyst, forming a catalytic salt. The second one is needed to both bridge the reacting partners and to act as a proton exchanger to avoid charge separation after the dienamine attack. For this reason, using two equivalents of acid promotes the reaction by allowing the formation of cyclical transition states. The importance of achieving this compact geometry is underlined by the great energy difference between these transition states and analogue structures we obtained while pursuing the former. If the system lacks continuity, that is, if hydrogen bonding of the bridging *N*-Boc-l-phenylglycine molecule is not perfectly achieved, transition states resulted in 5–7 kcal/mol higher in energy (Figure 4). Our previous work underlined the ineffectiveness of acids with too low p*K*_a_ or too great steric bulk. Indeed, using trifluoroacetic acid and (*S*)-TRIP as cocatalysts, no product was observed.^6,8^ Moreover, the use of either l- or d-amino acid enantiomers of *N*-Boc-l-phenylglycine yielded the same diastereomeric ratio, showing no matched/mismatched behavior in the reaction stereocontrol. With a clear picture of the epimerization process and defined transition-state geometries, it is now clear that the amino acid plays an important role in defining the catalyst conformation and regulating the access to dienamine faces.

### 2.4 Refining Transition States: A Docking Approach

**Figure 5:**
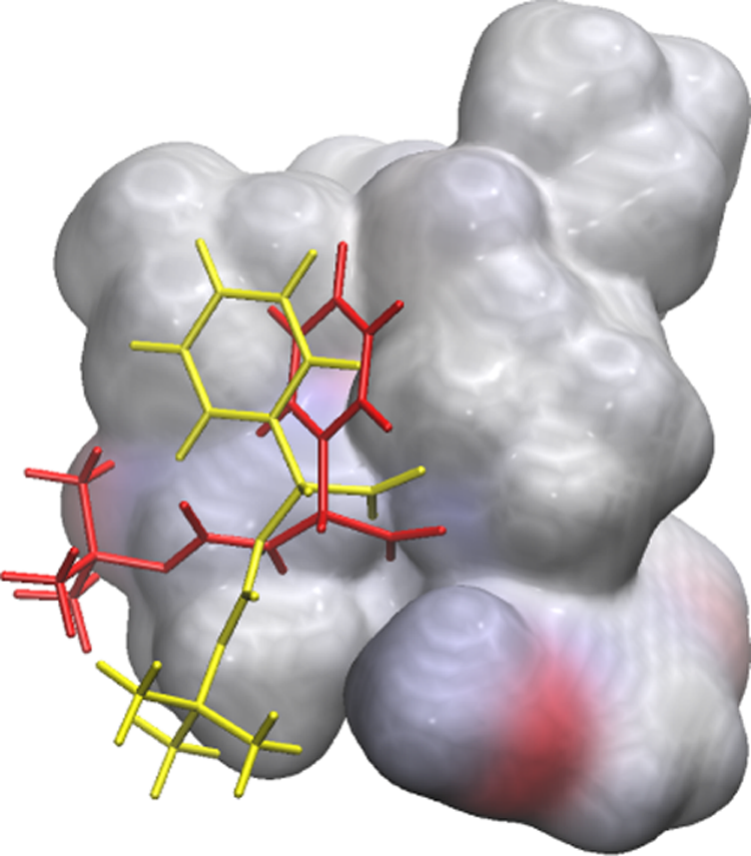
Transition state treated as a rigid receptor (gray surface) with two *N*-Boc-l-phenyl-glycine docked conformations: hand-positioned (yellow) and Autodock-generated (red). The red conformation is 4.22 kcal/mol more stable [ωB97X-D/6-311G(d,p)-CPCM (toluene) level of theory].

With eight transition states in hand (first iterations of **TS1**, **TS3**, **TS5**, and **TS7**, both **-*syn*** and **-*anti***) we wanted to explore the influence of amino acid conformation in transition-state energy. To do so, we decided to seek optimal conformations in an unusual way. Instead of a classical force field-guided conformational search, we used the docking software Autodock 4.2. Although the latter is not as fast as the latest Autodock Vina,^13^ it allows more flexibility in tweaking the parameters of the heuristic energetic contributions. Indeed, it is important to note that the software is optimized for water-solvated systems, while the reaction in this work is conducted in toluene. To better mimic the apolar environment of the reaction, the dielectric constant and desolvation map contribution were zeroed. By treating one *N*-Boc-l-phenylglycine molecule as a flexible ligand and the rest of the transition state as a rigid receptor, we were able to generate a series of poses for the amino acid. After running docking calculations, promising conformers were optimized along with the rest of the transition state with DFT methods. This process was repeated for the second equivalent of *N*-Boc-l-phenylglycine, where the first docked amino acid became part of the receptor for the new docking. Quantum chemical calculations proved the approach successful, as it afforded the final **TS1-*anti*** structure, 4.22 kcal/mol lower in energy than the previous in which the two amino acids were positioned manually (Figure 5).

These successful acid conformations obtained with Autodock were used to obtain **TS3-*anti*** since its only difference with **TS1-*anti*** is dienamine exocyclic double-bond geometry. Other transition states were treated similarly to obtain the best binding mode for the cocatalyst. After obtaining docking-optimized structures for transition states, a quasi-harmonic (qh) frequency analysis was carried with the GoodVibes program.^7^ Computed enthalpies and free energies were used for calculating Boltzmann distributions (Table 2).

**Table 2:** Refined TS Energies and Boltzmann distribution^a^

		Boltzmann distribution (%)		
name	rel. EE	EE^b^	qh-H^c^	qh-G^d^
TS1-*anti*	+0.00	53.27	40.54	36.89
TS1-*syn*	+8.32	0.00	0.00	0.00
TS3-*anti*	+0.08	46.71	59.05	60.24
TS3-*syn*	+6.33	0.00	0.01	0.19
TS5-*anti*	+5.88	0.00	0.04	0.10
TS5-*syn*	+10.31	0.00	0.00	0.00
TS7-*anti*	+4.68	0.02	0.36	2.56
TS7-*syn*	+6.95	0.00	0.00	0.02

a TS energies are reported in kcal/mol at the ωB97X-D/6-311G(d,p)-CPCM (toluene) level of theory.

b Based on electronic energy (Gaussian).

c Based on enthalpy (Gaussian and GoodVibes).

d Based on Gibbs free energy (Gaussian and GoodVibes).

It is important to note that these results may not reflect experimental products distribution since the products formed can undergo an epimerization process. To directly compare transition state and product distribution, the same transition-state calculations were also run on a nonepimerizable substrate, which is later discussed.

### 2.5 Enantioselectivity Rationalization

Data in Table 2 show how **TS1-*anti*** and **TS3-*anti*** are greatly favored among the other **TSs**. We rationalize this result with two observations: one regarding the enantioselectivity (atroposelectivity) favoring **TS1** and **TS3** over **TS5** and **TS7** and one about *anti*-**TSs** being favored over *syn*-**TSs** in all cases. The enantioselectivity issue can be addressed by comparing the **TS** disposition from the maleimide perspective: as an example, a superimposition of **TS3-*anti*** and **TS7-*anti*** is presented in the table below (Table 3). From this point of view, we observed that the site where the phenylglycine should bridge and activate the partners is more hindered and tighter in **TS7-*anti*** (red) than in **TS3-*anti*** (blue). In the former, the catalyst quinoline portion is oriented toward the acid, while in the latter, the closest fragment is the quinuclidine. A measure of this effect is obtained from the hydrogen bonding distances of the bridging acid, showing a stronger interaction with the less hindered site, in the favored **TS3-*anti***.

**Table 3:**
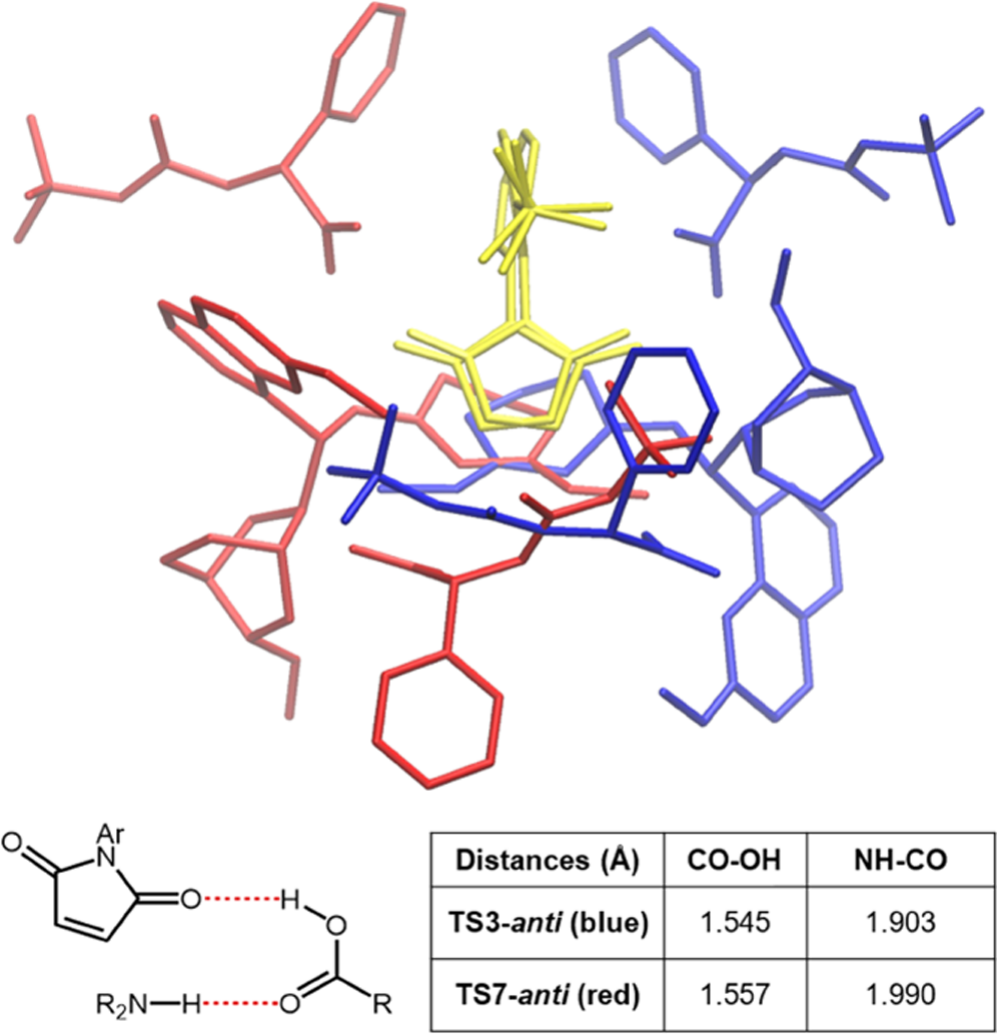
Hydrogen Bonding Distances in Enantiomeric Product Transition States^a^

a Superposition of **TS3-*anti*** (blue) and **TS7-*anti*** (red), aligned on maleimide (yellow). The blue **TS-*anti*** is favored, as the less hindered bridging acid interaction site allows for a better activating interaction. This effect is reflected in shorter hydrogen bonding distances for the red **TS3-*anti***.

**Figure 6:**
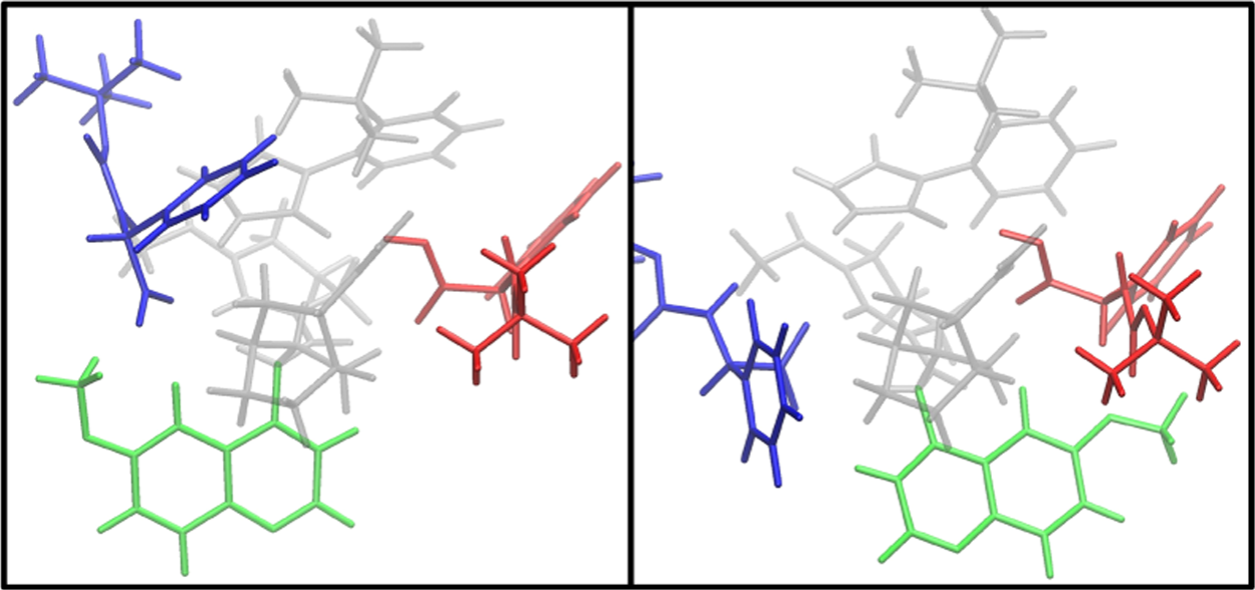
Quinoline conformation (green): preventing cyclical **TS** formation by shielding red *N*-Boc-l-phenyl-glycine in **TS1-*syn*** (right) and allowing access to the interaction site in **TS1-*anti*** (left).

Interestingly, we also noted how ***syn*-TSs** always appear less favorable than their ***anti*-** counterparts (Table 2). They only differ for the methoxyquinoline conformation, which is directed toward the quinuclidine-bound amino acid in ***anti-*TSs** and toward the bridging amino acid in ***syn*-TSs**. The latter is more unfavorable for the reaction because it is detrimental to the formation of a cyclical **TS** (Figure 6), once again hindering the bridging interaction site.

### 2.6 Epimerization Process

As previous work proved, an epimerization process is present in the reaction conditions due to the catalytic salt presence (Scheme 3). If the used cyclohexenone presents a hydrogen atom in the exocyclic γ-position, this process is responsible for equilibration between diastereoisomers formed in the reaction. Therefore, transition-state energies dictate product distribution only for nonepimerizable products, while epimerizable product distribution is only controlled by their thermodynamic stability. Thereby, a conformational search and DFT optimization of reaction products was carried, and results are presented in Table 4. Calculations correctly predict **3a** as the major diastereoisomer. The resulting distribution is also in good agreement with experimental results.

**Table 4:**
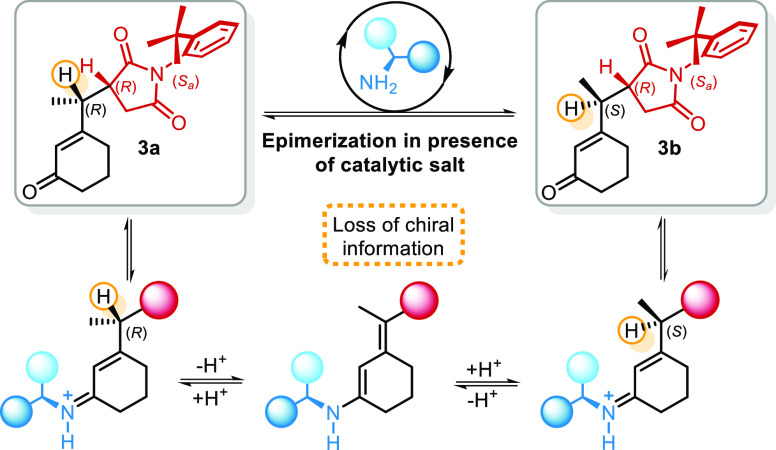
Conformational Search and DFT Optimization Results for Reaction Products that Undergo Epimerization^a^

		Boltzmann distribution (%)			
product	rel. E	EE^b^	qh-H^c^	qh-G^d^	exp^e^
**3a**	+0.00	83.15	81.85	80.05	70
**3b**	+0.95	16.85	18.15	19.85	30

a Product energies are reported in kcal/mol at the ωB97X-D/6-311G(d,p)-CPCM (toluene) level of theory.

b Based on electronic energy (Gaussian).

c Based on enthalpy (Gaussian and GoodVibes).

d Based on Gibbs free energy (Gaussian and GoodVibes).

e Experimental value from ^1^H NMR of the crude reaction mixture.

### 2.7 Nonepimerizable Product Comparison

**Figure 7:**
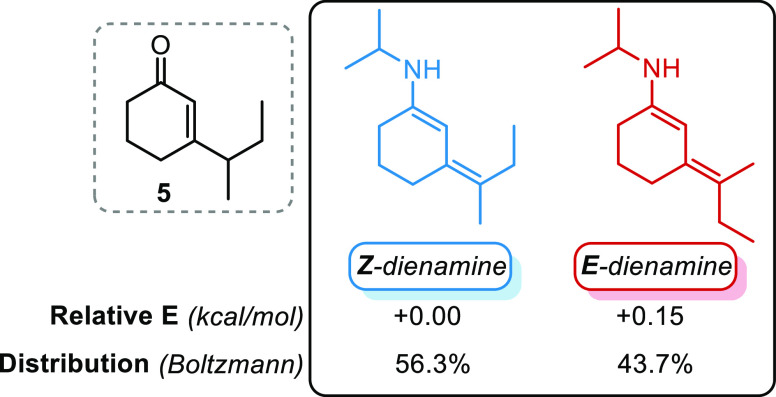
Relative energy for the most stable dienamine conformer found at the ωB97X-D/6-311G(d,p)-CPCM (toluene) level of theory.

Finally, to compare computational predictions with experimental data, **TS1-*anti*** and **TS3-*anti*** were also obtained for one nonepimerizable cyclohexenone. Cyclohexenone **6** was chosen because it was the simplest cyclohexenone having two different substituents on the γ-carbon atom. Conformational search and DFT optimization of the relative *E*- and *Z*-dienamines suggested that their presence in the reaction conditions is comparable, as for the previous case study (Figure 7).

Transition state obtainment for ketone **5** was limited to observed products forming **TS9-*anti*** and **TS10-*anti*** (Table 5). Results show that in this case, the favored transition state leads to the observed major product, as we expected since the exclusion of the epimerization process.

**Table 5:**
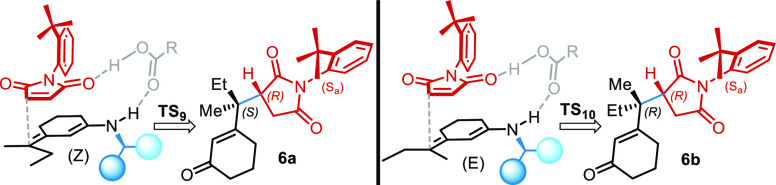
Transition-State Energies for Compounds **6a** and **6b**^a^

		Boltzmann distribution (%)			
name	Ts rel. EE	EE^b^	qh-H^c^	qh-G^d^	exp^e^
TS9	+0.00	88.13	91.18	93.79	75
TS10	+1.19	11.87	8.82	6.21	25

a Product energies are reported in kcal/mol at the ωB97X-D/6-311G(d,p)-CPCM (toluene) level of theory.

b Based on electronic energy (Gaussian).

c Based on enthalpy (Gaussian and GoodVibes).

d Based on Gibbs free energy (Gaussian and GoodVibes).

e Experimental value from ^1^H NMR of the crude reaction mixture.

**Figure 8:**
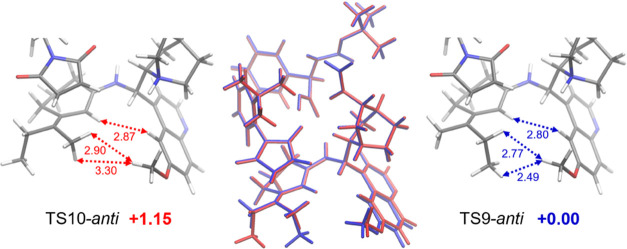
Key dispersion interactions favoring **TS9-*anti*** over **TS10-*anti***. Distances are in Ångström and relative energies are in kcal/mol at the ωB97X-D/6-311G(d,p), CPCM (toluene) level of theory.

The rationalization behind this selectivity is not trivial, and it may look counterintuitive at first glance. The favored **TS** is **TS9-*anti***, which differs from **TS10-*anti*** only by the dienamine exocyclic double-bond geometry. While the former may seem sterically unfavored over the latter, we surprisingly found that their difference allows a better establishment of dispersion interactions for the more hindered **TS9-*anti*** (Figure 8). The inner position of the longer alkyl chain favors the mutual interaction of it with the quinoline methoxy group, thereby bringing the two closer.

**Figure 9:**
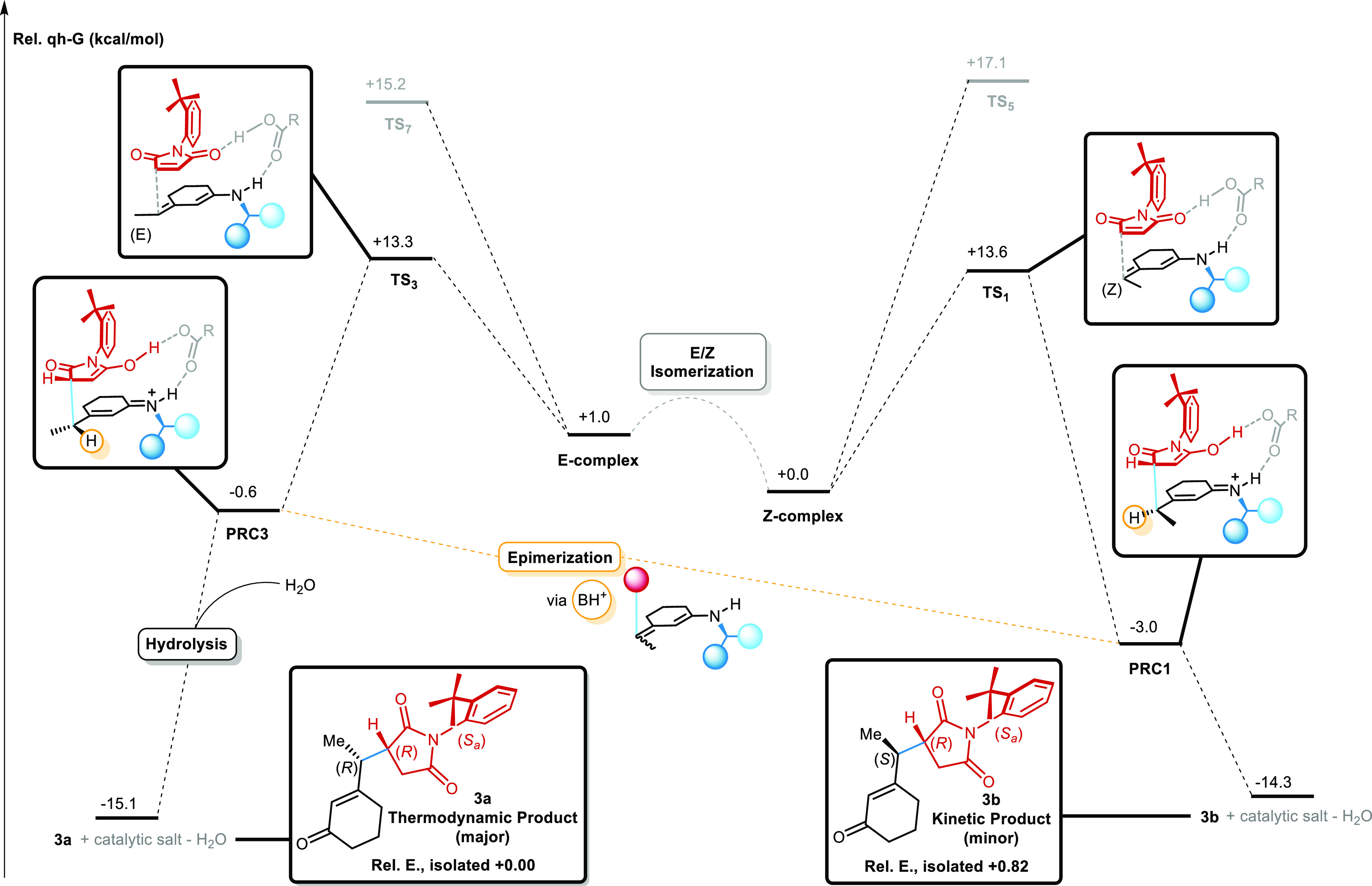
IRC diagram for the reaction of 3-ethyl-2-cyclohexenone **1** with maleimide **2** and catalyst 9-ADEQ. The epimerization process results in a kinetically controlled atroposelectivity and a thermodynamically controlled diastereoselectivity. Quasi-harmonic Gibbs free energies are obtained through GoodVibes.

**Figure 10:**
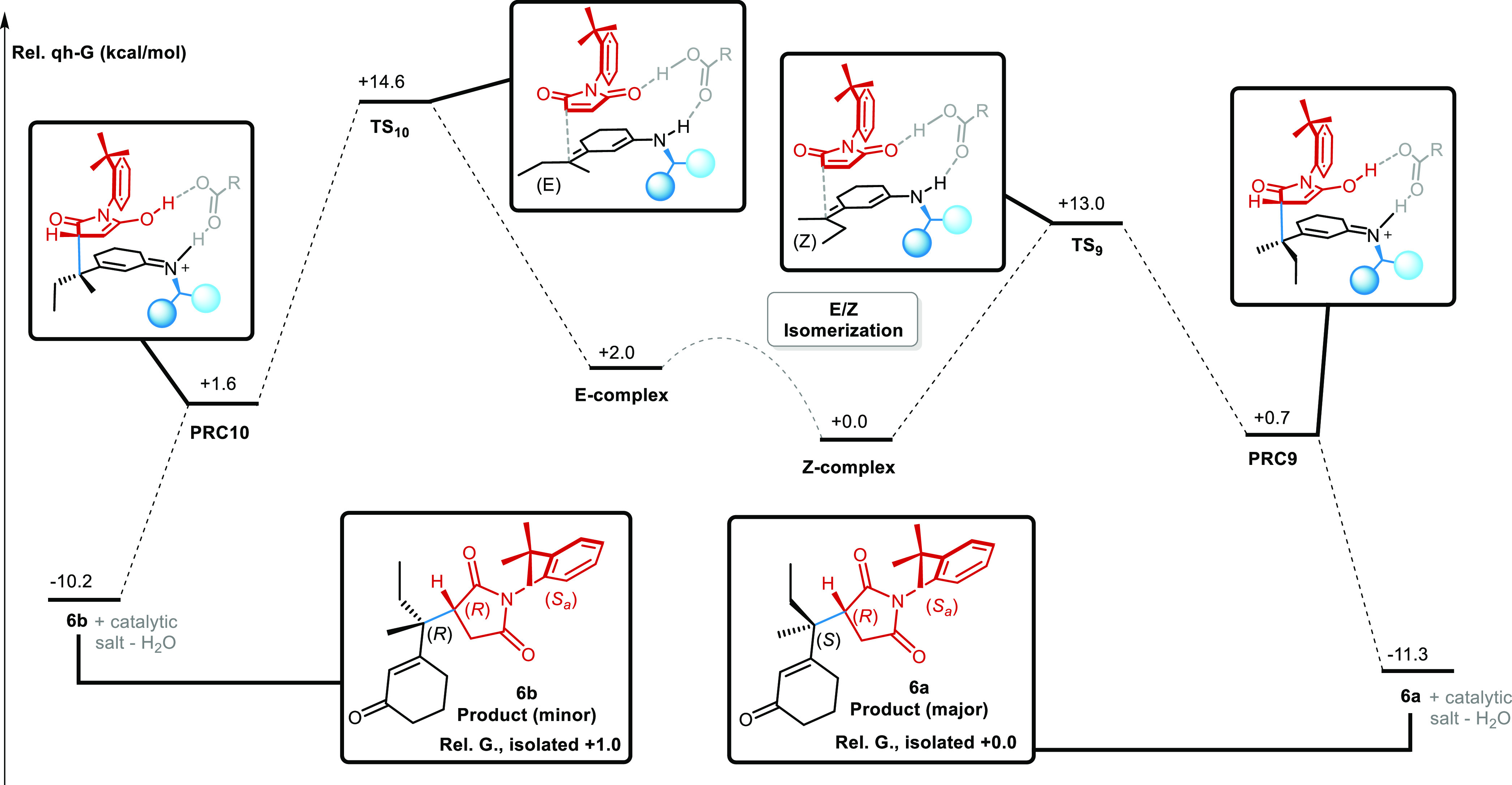
IRC diagram for the reaction of 3-(*sec*-butyl)-2-cyclohexone **5** with maleimide **2** and catalyst 9-ADEQ. The lack of epimerizable centers results in complete kinetic control of the reaction. Quasi-harmonic Gibbs free energies are obtained through GoodVibes.

This deformation also has the effect of approaching other atoms present in both **TSs**, improving their London dispersion bonding interaction. Further calculations with density functionals that allow dispersion contribution on–off switching further confirmed that the origin of the energy difference comes from dispersion interactions (see the Supporting Information for more details). These results, along with the IRCs of the two reactions analyzed (Figures 9 and 10), sustain the idea that the reaction initially forms the postreaction complexes (PRCs) irreversibly, under kinetic control. The most favored TS is the one that best satisfies steric, electronic, and dispersion interactions, namely, **TS1-*anti*** and **TS9-*anti***. Then, if epimerization is feasible, a path equilibrating these postreaction complexes is opened and thermodynamics shift product distribution toward the most stable free product: **3a** in our case study.

## 3 Conclusions

In summary, the mechanism and selectivity of the aminocatalytic atroposelective desymmetrization of maleimides by 3-alkylcyclohexenones has been elucidated using DFT computational studies. Transition states and intermediate species geometries have been identified, obtaining IRC diagrams.

The first issue we addressed was the energetic distribution and possibility of interconversion between isomeric dienamines arising from cyclohexenones **1** and **6**. The energy difference between *E*- and *Z*-geometric isomers of these species is minimal, and both are present in solution.

We obtained different **TSs** leading to observed products and we rationalized the quinine-derived primary amine catalyst stereoselectivity. Reacting partners can only achieve a proper orbital overlap and contiguity in ***endo-*TSs**, while ***exo*-TSs** are unfavorable (Scheme 6).

The atroposelection is controlled by the catalyst geometry, which favors the attack of one maleimide β-carbon atom over the other (Scheme 3 and Table 2). Observed *S*_a_ products arise from *endo* transition states involving the “upper” dienamine face (**TS1** and **TS3**), while enantiomerical *R*_a_ products should arise from ***endo*-TSs** involving the “lower” dienamine face (**TS5** and **TS7**). The latter show a less favorable disposition of the reagents because the increased quinoline steric bulk around the CO–NH site jeopardizes the bridging acid interaction (Table 2).

On the other hand, diastereoselection is catalyst-controlled only if enone lacks hydrogen atoms at the γ-position, allowing for a kinetic control of the reaction. Otherwise, an epimerization process takes place, equilibrating two diastereomeric products via an intermediate that loses the chiral information at the γ-position (Table 3), favoring the most stable of the two epimers.

For nonepimerizable γ,γ-disubstituted ketones, the rationale behind diastereoselectivity lies in attractive dispersion interactions. As recent works by Schreiner^14^ and Bistoni^15^ showed, dispersion forces play a crucial role in shifting the energy of organocatalytic reaction transition states. Their contribution is as important as the steric repulsion, and a careful balance of the two must be in place to obtain the maximum ΔΔ*G*^‡^ possible for diastereomeric **TSs**.

The role of the acidic cocatalyst has been explored as well, and the requirement of 2 molecules of acid has been clarified. While the first molecule reacts with the catalyst forming the catalytic salt, the second bridges reacting partners in a cyclical fashion. The effectiveness of *N*-Boc-l-phenylglycine is traced back to both its p*K*_a_ value and its stereoelectronic characteristics that allow regulation of access to the dienamine faces while still being able to bridge the reacting partners.

Moreover, it is now clear that the total absence of cocatalyst stereochemical induction in previous experiments is due to the domination of the epimerization process in the experimental conditions. Therefore, l- and *N*-Boc-d-phenylglycine enantiomers may indeed show a matched/mismatched stereochemical induction behavior when catalyzing the reaction of γ,γ-disubstituted-*α,β*-unsaturated ketones.

Acid conformation in transition-state geometries has been explored using Autodock 4.2, a popular docking software. The amino acids were treated as flexible ligands, while the rest of the transition state was used as a rigid receptor. This unusual approach proved itself effective, and we believe docking programs and analogous heuristic software have an underexplored potential in investigating organocatalytic transition-state conformational space, particularly after the required tailoring of parameters to the problem in hand.

## 4 Computational Methods

Conformational searches were performed with the OPLS3 force field^16^ implemented in Macromodel 11.9, part of Schrödinger’s Maestro suite. Low energy conformations were located by a Monte Carlo multiple minimum (MCMM) method following the protocol by Willoughby et al.^17^

Quantum mechanical calculations were run with Gaussian 16.^18^ Methods for the stepwise refinement of the optimized geometries were taken from Gaussian’s introductory guide text, suggesting an initial B3LYP/3-21G level with the keyword opt = tight, followed by a refinement at the B3LYP/6-31G(d) level of theory. Subsequently, the theory level was increased to ωB97X-D/6-31G(d), and in all cases, was finally optimized at the ωB97X-D/6-311G(d,p), accounting for the toluene environment by adopting the CPCM solvation model. Large conformational ensembles (>100 molecules) were often pruned by semiempirical PM6 calculations prior to ab initio methods. TSs were sought by different approaches, and among the strategies tested, the only successful one is reported in detail in the Supporting Information.

Docking calculations were performed using Autodock 4.2.^19^ One *N*-Boc-l-phenylglycinate ion was used as a ligand, while the rest of the transition state, including the second amino acid, was used as a rigid receptor. This process was repeated for both *N*-Boc-l-phenylglycine molecules. Anchors for the ligands (bound docking) were, respectively, the hydrogen atom of the quinuclidine NH moiety and the hydrogen atom of the dienamine NH moiety. A grid box of 8 Å × 8 Å × 8 Å size was centered on the respective anchor point. Before running the docking, contributions from the desolvation map potential were zeroed by manually editing the receptor.d.map, substituting every coefficient with 0.000. The dielectric constant was also zeroed from the grid parameter file (.gpf). All other preparation steps were conducted as per the standard Autodock protocol,^20^ using AutoDockTools (ADT) to add Gasteiger–Marsili partial charges to the structures. Hydrogen atoms were already present in all structures and were retained in structure preparation. The search algorithm used was Lamarckian GA. In some cases, due to nondeterministic nature of the docking process and the peculiar use of the program, more than one docking was run to obtain more poses for each amino acid. In each case, only poses possessing an amino acid carboxylate–receptor NH interaction were kept for DFT optimizations of the entire system, as described in the Supporting Information. Poses that did not possess such a hydrogen bond were discarded. TS structures are the ones with the lowest energy after DFT optimization. Ligand structures are conformations obtained with Autodock, before DFT optimization with the rest of the system.

Frequency analysis was performed using the GoodVibes program from the Paton group.^7^ All reported free energies and enthalpies are calculated at the reaction temperatures, which is 298 K for the desymmetrization reactions and 403 K for the chiral axis racemization reaction. Anharmonic correction used was the Grimme/Head-Gordon (-q keyword) and the solvent used was toluene (--freespace toluene). The frequency cutoff used was the default value (100 cm^–1^).

## Supporting Information Available

Quantitative dispersion assessment, computational strategy and IRC diagrams construction, and geometries and energies of calculated stationary points (PDF)
