# Supplementary material for: Computational Investigation on the Origin of Atroposelectivity for the Cinchona Alkaloid Primary Amine-Catalyzed Vinylogous Desymmetrization of N-(2-t-Butylphenyl)maleimides
Source: J Org Chem. 2021 Aug 4;86(17):11782–93. doi: 10.1021/acs.joc.1c01235 (PMC8764656; doi:10.1021/acs.joc.1c01235)
Supplement: Supplementary file 1 — jo1c01235_si_001.pdf [file jo1c01235_si_001.pdf]

A Computational Investigation on the Origin of Atroposelectivity for the Cinchona Alkaloid Primary Amine Catalyzed Vinylogous Desymmetrization of *N*-(2-*t*-butylphenyl)maleimides.

Nicolò Tampellini*, Paolo Righi, Giorgio Bencivenni*.

Contents

1. **Quantitative dispersion assessment** **S1**
2. **Computational strategy** **S3**
3. **IRC diagrams** **S5**
4. **Geometries and Energies of Calculated Stationary Points** **S7**

1. Quantitative dispersion assessment

In order to isolate the contribution of dispersion forces in stabilizing TS9-*anti* over TS10-*anti*, single point energy calculations were run. Using TS geometries calculated at ωB97X-D/6-311G(d,p), CPCM(Toluene) level, we ran two single point energy calculations for each structure with both B3LYP-D3(BJ) and B3LYP functionals so as to isolate the dispersion contribution by "turning off" a portion of the density functional. Interestingly, a negligible energy difference is present between TSs with the plain B3LYP functional, while including the D3(BJ) correction splits the two states more than one kcal/mol apart. This energy difference is also very similar to the one obtained with the ωB97X-D functional (1.15 kcal/mol). We conclude that not only differential dispersion interactions are important in determining stereoselectivity, but in this case study they are the <u>only</u> responsible for diastereoselectivity.

Transition State	Single point Electronic Energy	Rel. E (kcal/mol)
TS9-anti_B3LYP	-3877.522607	0.000
TS10-anti_B3LYP	-3877.522540	0.042
TS10-anti_B3LYP-D3(BJ)	-3878.025585	1.170
TS9-anti_B3LYP-D3(BJ)	-3878.027449	0.000

Table 1S. Single point energy of TS9-anti and TS10-anti at B3LYP/6-311G+(d,p), CPCM(Toluene)//ωB97X-D/6-311G(d,p), CPCM(Toluene) and B3LYP-D3(BJ)/6-311G+(d,p), CPCM(Toluene)//ωB97X-D/6-311G(d,p), CPCM(Toluene)

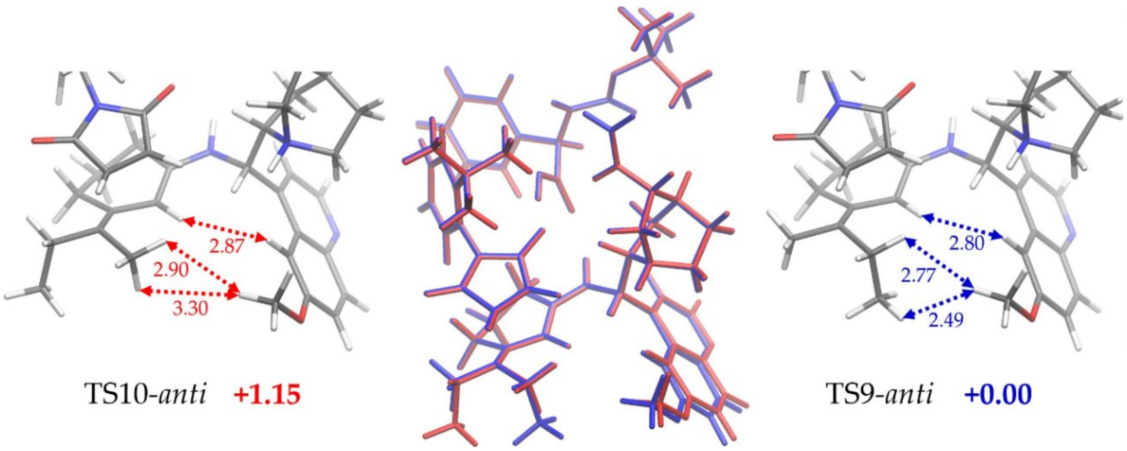

Figure 1S. Key dispersion interactions favoring TS9-anti over TS10-anti. Distances are in Ångström, relative energies are in kcal/mol at ωB97X-D/6-311G(d,p), CPCM(Toluene) level.

2. Computational strategy

Obtaining transition state structures, particularly for cyclical, contiguous arrangements of molecules, is not a standardized nor a trivial task. Our approach in seeking these arrangements mainly focused on a modular, incremental addition of molecular complexity along various steps. The theory level used in these initial calculations was high enough to obtain a good behavior from the density functional chosen (ωB97X-D/6-31G(d)) while fast enough to explore different techniques in a reasonable time. Firstly, a simplified analog of the transition state was built by substituting the two N-Boc-L-phenylglycine molecules with acetic acid, and an optimization to energetic minimum of the pre-reaction complexes was performed. Obtained this equilibrium geometry (Acetate EG), reacting partners were approached by means of scanning the reacting distance until about the length of a regular C-C bond (1.5 Å). The energetic maximum for the SCAN calculation was then directly used for a Berny optimization, obtaining the Acetate TS. At this point, acetic acid molecules were replaced with N-Boc-L-phenylglycine and a partial optimization (POPT) was performed, where the reacting C-C distance is constrained while the rest of the structure is left free to relax. The resulting structure was then re-optimized to a maximum with the Berny algorithm, allowing access to the desired transition state. These steps were all performed at ωB97X-D/6-31G(d) level. To raise the theoretical level, reacting C-C distance was again constrained, and another POPT was carried at ωB97X-D/6-311G(d,p), CPCM level. The resulting structure was then optimized again at the same level with Berny algorithm to afford the TS. Subsequent IRC calculations (forward and reverse), followed by optimization to energetic minima of the two endpoints, afforded both the equilibrium geometry before the reaction (EG) and the post-reaction complex (PRC), confirming the nature of the transition state.

Analogs of transition states were also sought from related structures: TS3-*anti* was obtained from TS1-*anti* by swapping the hydrogen and methyl substituents in γ-position. Then, only steps 6 and 7 were required to afford the new TSs.

Troubleshooting and difficulties in following this protocol were resolved either by tight convergence of the partial optimization steps (*opt=tight*) or by manually adjusting molecular fragments before running the calculation.

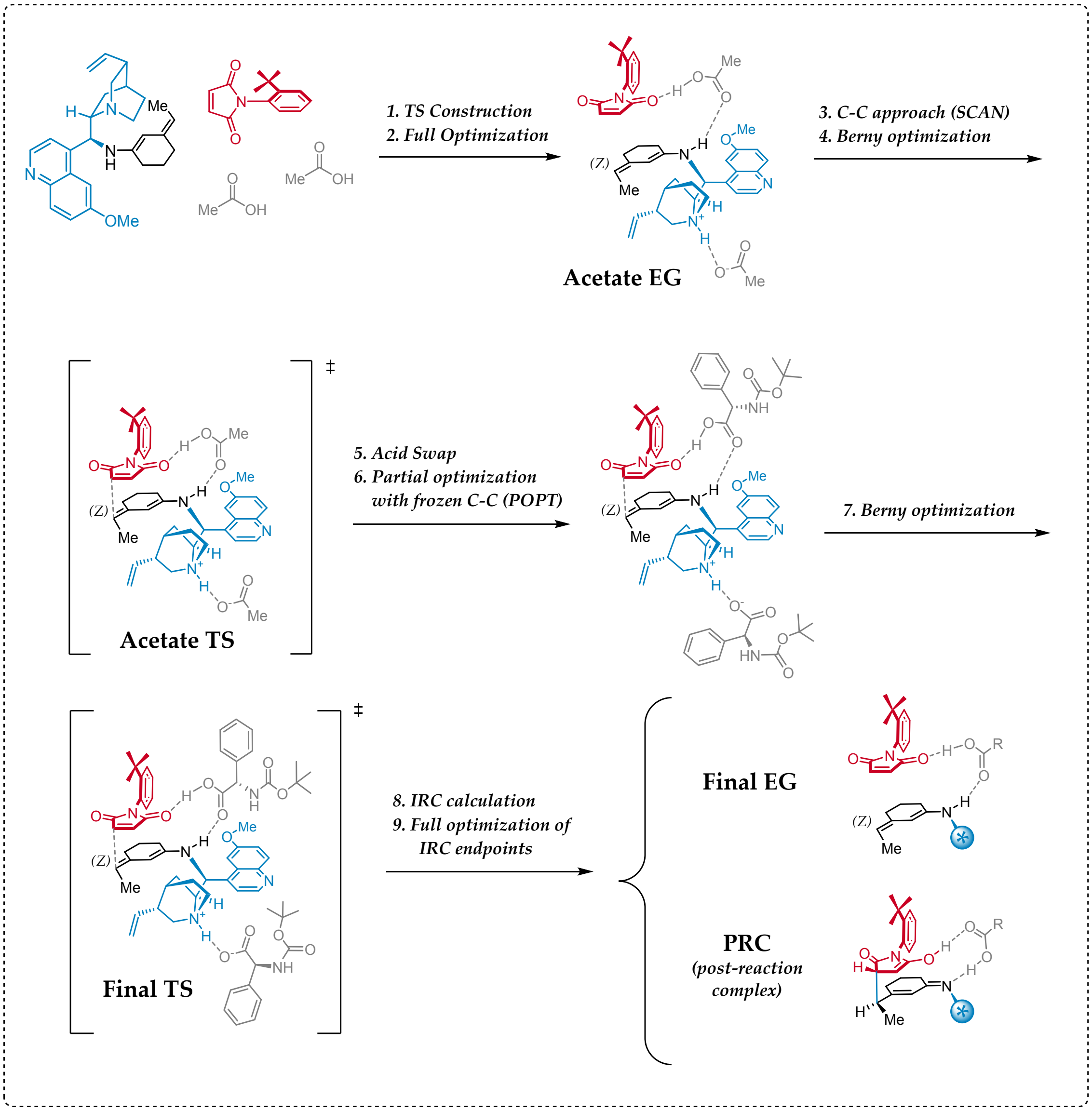

Scheme 1S. Steps performed to obtain and validate the desired transition states.

3. IRC diagrams

IRC diagrams were constructed as per common practice, at constant atom number. Hydrolyzed products (i.e., **3a**) are coupled with the catalytic salt, and their energy is obtained as:

$$E_{tot,\,4a} = E_{4a,\,isolated} + E_{cat.\ salt} - E_{H_2O}$$

Where the catalytic salt is represented by one molecule of 9-ADEQ optimized with two N-Boc-L-phenylglycine molecules. The energy of a water molecule at the same level of theory is subtracted to keep a constant number of atoms throughout the whole diagram.

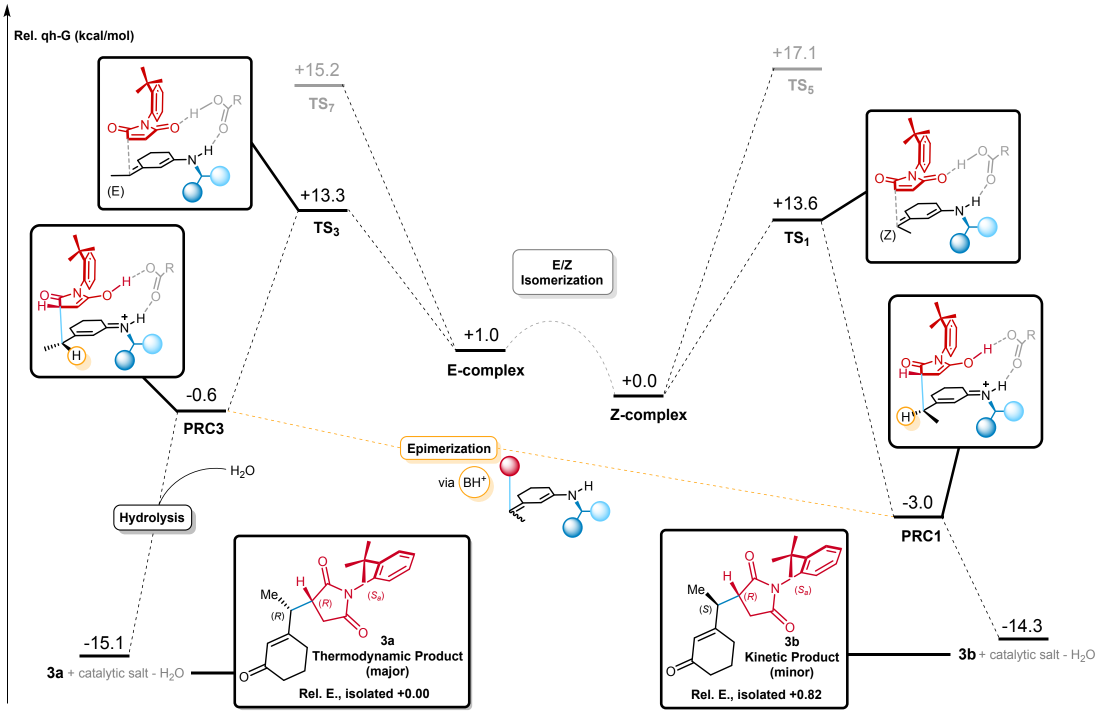

Figure 2S: IRC diagram for the reaction of 3-ethyl-2-cyclohexenone 1 with maleimide 2 and catalyst 9-ADEQ. The epimerization process results in a kinetically controlled atroposelectivity and a thermodynamically controlled diastereoselectivity.

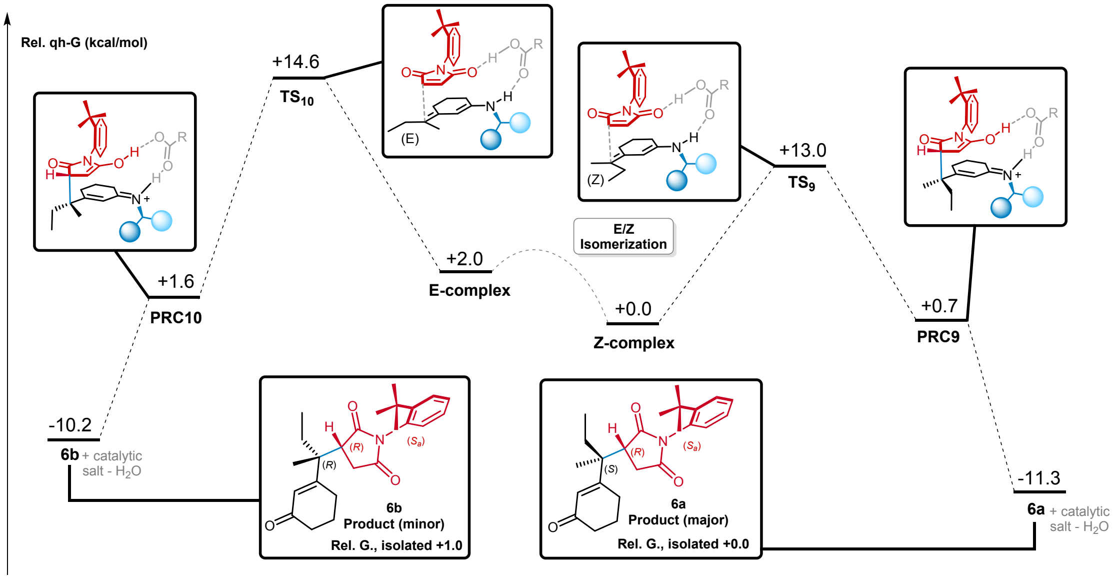

Figure 3S: IRC diagram for the reaction of 3-(sec-butyl)-2-cyclohexone 5 with maleimide 2 and catalyst 9-ADEQ. The lack of epimerizable centers results in a complete kinetic control of the reaction.

4. Geometries and Energies of Calculated Stationary Points

Below are reported all the structures cited in this work. When an energy value is specified, the computational level is ωB97X-D/6-311G(d,p), CPCM(Toluene). Species are labeled as in IRC schemes.

1. 3a
Imaginary Frequencies = 0 (Equilibrium Geometry)
Energy (Hartree) = -1135.00945815

C	-5.996782	-0.529731	-0.704021
C	-4.867122	-1.547736	-0.834933
C	-3.903671	-1.438280	0.343741
C	-3.440727	-0.021576	0.570317
C	-4.167919	1.022271	0.146785
C	-5.471607	0.880582	-0.529322
O	-6.099735	1.852783	-0.897328
C	-2.137848	0.190823	1.306899
C	-0.955608	-0.365695	0.483960
C	-0.960103	-0.020000	-1.007081
C	0.471064	0.317809	-1.352322
N	1.193668	0.363217	-0.161371
C	0.400846	0.135065	0.963810
O	0.774217	0.295965	2.095833
O	0.927976	0.539055	-2.442502
C	2.536547	0.859613	-0.064642
C	2.643694	2.241956	0.048099
C	3.879806	2.853254	0.164151
C	5.012725	2.055640	0.170581
C	4.897125	0.676863	0.059295
C	3.665269	0.022993	-0.062847
C	-2.197158	-0.430046	2.708574
C	3.619501	-1.508726	-0.239146
C	2.563402	-2.177413	0.663931
C	4.971959	-2.153166	0.113091
C	3.320372	-1.831207	-1.714710
H	-0.917029	-1.454876	0.607948
H	-6.597230	-0.755070	0.187393
H	-6.672596	-0.541365	-1.560570
H	-4.317464	-1.362090	-1.764274
H	-5.272970	-2.559804	-0.898209
H	-3.042679	-2.096303	0.193727
H	-4.392158	-1.791136	1.261589
H	-3.831042	2.042428	0.306315
H	-1.989458	1.271063	1.409427
H	-1.321916	-0.819889	-1.653125
H	-1.566407	0.866189	-1.215453
H	1.734602	2.832152	0.045632
H	3.952746	3.930451	0.250842
H	5.996395	2.501438	0.263571
H	5.808435	0.096863	0.071202
H	-3.048707	-0.030612	3.264247
H	-2.305666	-1.517151	2.650762
H	-1.282260	-0.209157	3.256489
H	1.553233	-2.057368	0.273874
H	2.756896	-3.252021	0.711910
H	2.593431	-1.779145	1.680546
H	5.772806	-1.834215	-0.557759
H	5.269418	-1.935095	1.142783
H	4.883295	-3.237435	0.011416
H	4.101627	-1.424431	-2.362736
H	3.287512	-2.915870	-1.857781
H	2.369261	-1.412246	-2.043468

2. 3b
Imaginary Frequencies = 0 (Equilibrium Geometry)
Energy (Hartree) = -1135.01096464

C	5.988381	-0.971796	0.391758
C	5.390034	-1.493261	-0.911470
C	3.871971	-1.338122	-0.901780
C	3.442113	0.065394	-0.549807
C	4.240987	0.870497	0.165516
C	5.558635	0.451052	0.682477
O	6.244534	1.209710	1.337613
C	2.070312	0.483997	-1.026883
C	1.005830	-0.437011	-0.376339
C	0.867777	-0.300768	1.141016
C	-0.522662	0.245150	1.373688
N	-1.196966	0.230976	0.147015
C	-0.372386	-0.098340	-0.920348
O	-1.001868	0.618883	2.409048
O	-0.701697	-0.091480	-2.078917
C	-2.522015	0.758086	-0.007770
C	-2.584835	2.135072	-0.195464
C	-3.800796	2.777540	-0.349011
C	-4.958660	2.017561	-0.307286
C	-4.887169	0.644505	-0.116336
C	-3.676426	-0.040675	0.040809
C	-3.672557	-1.575318	0.196499
C	-2.733934	-2.054118	1.322569
C	-3.246937	-2.210764	-1.139855
C	-5.076537	-2.105476	0.537873
C	1.742976	1.963570	-0.834543
H	1.208402	-1.469748	-0.668410
H	7.078821	-1.009493	0.398171
H	5.637405	-1.584553	1.232894
H	5.661125	-2.540465	-1.062710
H	5.802531	-0.927781	-1.753700
H	3.438189	-2.033189	-0.169740
H	3.449728	-1.615049	-1.872665
H	3.954482	1.884308	0.421360
H	2.027435	0.267596	-2.101128

H	0.943451	-1.260047	1.657546
H	1.601122	0.367225	1.595956
H	-1.657728	2.696541	-0.214353
H	-3.839793	3.850094	-0.494787
H	-5.928002	2.489154	-0.421071
H	-5.816991	0.095201	-0.087231
H	-2.843595	-1.445920	2.223068
H	-2.974207	-3.089362	1.577455
H	-1.686876	-2.039581	1.021217
H	-3.946398	-1.938346	-1.935062
H	-2.253045	-1.888641	-1.451116
H	-3.242485	-3.301321	-1.046715
H	-5.796413	-1.926582	-0.263929
H	-5.020306	-3.187326	0.680206
H	-5.463020	-1.664325	1.461051
H	2.507581	2.590038	-1.297138
H	0.790434	2.203224	-1.312349
H	1.674537	2.243957	0.220236

3. 3b_atropoTS1

Imaginary Frequencies = 1 (Transition State)

Energy (Hartree) = -1134.95788719

C	6.292166	-0.084203	-0.644580
C	5.383769	0.854941	-1.433656
C	4.336379	1.482323	-0.519361
C	3.607566	0.460624	0.315405
C	4.158860	-0.730683	0.589123
C	5.502850	-1.124015	0.123845
O	5.959299	-2.220557	0.378315
C	2.241283	0.868021	0.817137
C	1.216704	0.736684	-0.338912
C	0.856059	-0.684087	-0.726231
C	-0.509462	-0.868749	-0.125379
N	-1.160323	0.410105	-0.105506
C	-0.111046	1.353586	0.066209
O	-0.921356	-1.898277	0.317889
O	-0.187653	2.468399	0.513006
C	-2.581382	0.786388	-0.079686
C	-2.787318	2.173047	-0.178546
C	-4.029660	2.772018	-0.098087
C	-5.143680	1.981167	0.087439
C	-4.971489	0.610272	0.083764
C	-3.741703	-0.051029	-0.052034
C	-3.839777	-1.587335	-0.196485
C	-5.273975	-2.072655	-0.522190
C	-3.510499	-2.250396	1.161320
C	-3.020676	-2.062887	-1.427065
C	1.779662	0.144348	2.081953
H	1.568237	1.320645	-1.192672
H	6.862608	0.489413	0.098089
H	7.014468	-0.600337	-1.278905
H	4.876156	0.290135	-2.223245
H	5.974147	1.632630	-1.923272
H	3.620261	2.069578	-1.101650
H	4.811651	2.194003	0.169722
H	3.651796	-1.470908	1.198648
H	2.288877	1.940047	1.039531
H	0.763177	-0.800783	-1.809033
H	1.535015	-1.450684	-0.357246
H	-1.944835	2.826595	-0.295664
H	-4.102946	3.850553	-0.171562
H	-6.134136	2.407040	0.195663
H	-5.862363	0.008082	0.166469
H	-5.686639	-1.582321	-1.407739
H	-5.216351	-3.144718	-0.731957
H	-5.972842	-1.950684	0.308065
H	-2.614463	-1.858237	1.626677
H	-4.351469	-2.083749	1.841407
H	-3.383264	-3.330057	1.037851
H	-2.188993	-1.415561	-1.692094
H	-2.626651	-3.068913	-1.271120
H	-3.672747	-2.084639	-2.304405
H	2.530982	0.237752	2.867936
H	0.851291	0.584927	2.453458
H	1.601191	-0.921350	1.918030

4. 3b_atropoTS2

Imaginary Frequencies = 1 (Transition State)

Energy (Hartree) = -1134.95753155

C	-6.227763	-0.162608	-0.387810
C	-5.280383	-0.329656	-1.572902
C	-4.057320	-1.148250	-1.171732
C	-3.404774	-0.636440	0.086577
C	-4.094103	0.086625	0.980820
C	-5.524227	0.413784	0.823587
O	-6.108830	1.086720	1.648815
C	-1.943414	-0.980484	0.266948
C	-1.107273	-0.028794	-0.621536
C	-1.143439	1.444298	-0.287469
C	0.281374	1.879436	-0.478275
N	1.144820	0.743404	-0.268408
C	0.367066	-0.392714	-0.586354
O	0.607802	2.987737	-0.797114
O	0.748441	-1.498965	-0.846974
C	2.592122	0.894395	-0.040500
C	2.973886	2.220313	0.228827
C	4.287266	2.639986	0.312733
C	5.295845	1.719512	0.118884
C	4.938593	0.391139	-0.006401
C	3.623508	-0.099271	-0.009353
C	3.515919	-1.639416	0.122389
C	2.456497	-2.004796	1.194266
C	4.812403	-2.275402	0.687629
C	3.348118	-2.286276	-1.270511
C	-1.467755	-1.003202	1.718456
H	-1.408180	-0.173111	-1.663425

H	-6.620724	-1.142820	-0.086531
H	-7.084425	0.471208	-0.621978
H	-4.951866	0.657322	-1.916578
H	-5.797450	-0.805570	-2.409051
H	-3.328452	-1.174320	-1.987119
H	-4.341875	-2.194721	-0.995090
H	-3.638381	0.453848	1.894078
H	-1.793083	-1.982052	-0.151410
H	-1.799063	2.042420	-0.916464
H	-1.410103	1.635012	0.756412
H	2.222151	2.976168	0.345754
H	4.498198	3.684354	0.509122
H	6.340227	2.008030	0.116133
H	5.742328	-0.326109	-0.066984
H	1.654271	-1.281437	1.289818
H	2.009349	-2.978769	0.984529
H	2.940112	-2.056707	2.173962
H	5.645249	-2.271658	-0.018592
H	5.134801	-1.798167	1.616606
H	4.593037	-3.324061	0.908834
H	4.294237	-2.197098	-1.813620
H	3.119088	-3.351584	-1.166909
H	2.568077	-1.829840	-1.866929
H	-2.125738	-1.630316	2.322755
H	-0.461020	-1.420492	1.779557
H	-1.446683	-0.010360	2.174524

5. 6a

Imaginary Frequencies = 0 (Equilibrium Geometry)

Energy (Hartree) = -1213.63575515

C	-5.744405	-1.307312	-0.364692
C	-4.636684	-2.020577	0.402797
C	-3.747703	-1.011209	1.122915
C	-1.955031	0.796889	0.612594
C	-0.799134	-0.261229	0.631311
C	-0.660519	-1.078608	-0.657938
C	3.945868	-1.423722	0.564882
C	3.760387	-2.487552	-0.532967
C	2.897087	-1.635139	1.675218
C	5.323099	-1.654723	1.212766
C	-1.581482	1.926479	-0.356488
H	-0.936781	-0.912900	1.497069
H	-6.401740	-0.778671	0.338532
H	-6.369106	-1.992526	-0.939793
H	-4.025719	-2.602856	-0.295757
H	-5.062743	-2.724180	1.121474
H	-2.915904	-1.527713	1.607037
H	-4.313177	-0.532857	1.933793
H	-0.700912	-2.154975	-0.477286
H	-1.419128	-0.851113	-1.408129
H	4.544611	-2.399108	-1.289966
H	3.820118	-3.488312	-0.093647
H	2.800216	-2.390467	-1.040439
H	2.840114	-0.773207	2.343434
H	1.902495	-1.827748	1.273954
H	3.172707	-2.510671	2.268427
H	5.545279	-0.898861	1.971444
H	5.324391	-2.632314	1.700738
H	6.133329	-1.664115	0.480498
H	-0.716249	2.472321	0.026997
H	-1.333775	1.550763	-1.353389
C	-2.143588	1.366474	2.042636
H	-1.204855	1.833767	2.344222
H	-2.300891	0.533495	2.735597
C	-3.289228	2.365500	2.183791
C	2.662371	1.838332	-1.117408
H	1.708228	2.266580	-1.402846
C	3.843168	2.511768	-1.375894
H	3.828025	3.476444	-1.868185
C	2.668973	0.597898	-0.487217
C	3.860575	-0.025913	-0.082542
C	5.033902	0.686544	-0.359200
C	5.036574	1.923489	-0.988664
C	-5.183962	-0.264382	-1.306169
O	-5.757848	0.059802	-2.326451
C	0.700696	-0.746608	-1.219908
C	0.572241	0.380355	0.810299
N	1.370571	0.026606	-0.274797
O	1.156623	-1.082229	-2.281018
O	0.945956	1.074451	1.720381
C	-3.235653	0.072113	0.201495
C	-3.913472	0.378322	-0.915093
H	-3.576956	1.146707	-1.600467
H	5.988668	0.268966	-0.074248
H	5.979857	2.424595	-1.173327
H	-3.390367	2.673059	3.226915
H	-3.115148	3.267485	1.592641
H	-4.245473	1.939712	1.866102
H	-2.395443	2.643136	-0.470264

6. 6b

Imaginary Frequencies = 0 (Equilibrium Geometry)

Energy (Hartree) = -1213.63499263

C	3.707561	2.587171	-0.237173
C	2.268819	2.266250	-0.624322
C	2.156201	0.829190	-1.123623
C	2.208062	-1.602964	-0.180613
C	0.756718	-1.671248	0.416757
C	0.612834	-1.046123	1.810408
C	-3.929065	-0.886814	0.323536
C	-4.137869	-1.766050	-0.923261
C	-5.289923	-0.643187	1.009121
C	-3.079546	-1.669389	1.341395
C	2.209476	-2.117632	-1.641566
H	0.497656	-2.733822	0.436442
H	3.819784	3.589313	0.179298

H	4.352761	2.527556	-1.123734
H	1.914753	2.958900	-1.391478
H	1.625351	2.396183	0.253665
H	2.702813	0.724678	-2.070396
H	1.119440	0.595509	-1.367854
H	0.219237	-1.751624	2.545329
H	1.537641	-0.632290	2.212317
H	-3.187463	-1.946326	-1.430546
H	-4.570180	-2.729465	-0.635071
H	-4.817990	-1.285903	-1.632121
H	-5.184302	0.036421	1.859279
H	-6.041727	-0.233139	0.333727
H	-5.681603	-1.593965	1.379611
H	-2.769718	-1.042361	2.181194
H	-3.686365	-2.483721	1.744872
H	-2.202434	-2.132862	0.890671
H	1.741454	-3.107660	-1.646887
H	1.567203	-1.488101	-2.257202
C	3.596055	-2.204338	-2.276879
H	3.506540	-2.471478	-3.332056
H	4.221822	-2.960695	-1.798354
H	4.127479	-1.249566	-2.219491
C	3.053427	-2.556595	0.679127
H	2.708097	-3.582379	0.525542
H	2.975957	-2.337313	1.746088
H	4.108756	-2.511880	0.407757
C	-1.549083	2.079282	-0.559921
H	-0.495891	2.320157	-0.486164
C	-2.439498	2.987599	-1.099700
H	-2.093939	3.952120	-1.451047
C	-1.978332	0.835483	-0.100930
C	-3.327651	0.457323	-0.140597
C	-4.195731	1.393415	-0.722012
C	-3.776469	2.627425	-1.190825
C	4.245823	1.588660	0.763132
O	5.123415	1.873852	1.552749
C	-0.395229	0.068762	1.663660
C	-0.350720	-1.009994	-0.395460
N	-0.937971	-0.021130	0.383098
O	-0.719149	0.882229	2.487089
O	-0.714468	-1.293738	-1.509608
C	2.727892	-0.166831	-0.139236
C	3.681358	0.224365	0.720677
H	4.126571	-0.457289	1.435322
H	-5.245196	1.149901	-0.816987
H	-4.499756	3.307714	-1.625419

C	1.876487	0.744203	-0.197457
C	2.152669	-0.619854	-0.445178
C	3.267032	-1.229804	0.081469
H	5.046470	-0.959606	1.309675
H	4.599377	1.418104	1.743861
C	0.735651	1.438357	-0.708981
H	1.496269	-1.226471	-1.054678
C	0.615692	2.769994	-0.411375
C	1.581060	3.409225	0.394412
H	-0.219354	3.330848	-0.812206
H	1.467773	4.465168	0.628111
N	2.629117	2.808115	0.896336
O	3.432186	-2.541539	-0.219130
C	4.563864	-3.219281	0.293634
H	4.563870	-3.230126	1.388580
H	4.489389	-4.241087	-0.073120
H	5.496703	-2.774902	-0.068796
C	-0.347191	0.757478	-1.525821
H	0.052734	-0.182534	-1.924922
N	-0.825825	1.639549	-2.585203
H	-0.140325	1.702452	-3.328520
H	-1.668656	1.220490	-2.965914
C	-1.552573	0.428193	-0.626329
H	-1.947372	1.399945	-0.309548
C	-1.231343	-0.422350	0.625436
C	-3.877359	-0.076425	-0.656577
C	-2.355071	-1.620205	-1.650567
C	-2.384719	-1.415057	0.831226
H	-0.304720	-0.988498	0.497388
H	-1.085621	0.219158	1.497439
C	-3.751251	-0.692318	0.776633
H	-4.144672	0.982333	-0.597629
H	-4.668388	-0.579160	-1.219104
C	-2.339412	-2.424323	-0.322919
H	-1.399591	-1.707376	-2.172077
H	-3.119268	-1.999118	-2.333542
H	-2.280308	-1.924724	1.791628
H	-4.531627	-1.447967	0.915487
H	-1.436144	-3.036456	-0.249958
H	-3.197423	-3.100812	-0.263440
N	-2.630526	-0.197282	-1.413983
C	-3.908716	0.338937	1.854715
H	-3.252084	1.206628	1.793797
C	-4.783902	0.267475	2.850487
H	-5.462748	-0.575792	2.945963
H	-4.854557	1.045457	3.602760

7. 9-ADEQ_anti-open_external_OMe
Imaginary Frequencies = 0 (Equilibrium Geometry)
Energy (Hartree) = -1016.55735806

C	4.166899	-0.492116	0.887585
C	3.923295	0.831465	1.133491
C	2.787790	1.487023	0.604744

8. 9-ADEQ_anti-open_internal_OMe
Imaginary Frequencies = 0 (Equilibrium Geometry)
Energy (Hartree) = -1016.55989343

C	4.181449	0.630684	-1.150441
C	4.042840	-0.720620	-1.218186
C	2.965043	-1.383637	-0.569789

C	2.021793	-0.611289	0.158934
C	2.193886	0.800376	0.223534
C	3.243767	1.405493	-0.419033
H	4.997740	1.144940	-1.643234
H	4.746949	-1.332975	-1.768509
C	0.950370	-1.309422	0.789618
H	1.487023	1.396312	0.780703
C	0.917534	-2.676142	0.664030
C	1.905671	-3.342115	-0.083016
H	0.133745	-3.236027	1.158515
H	1.866632	-4.424240	-0.180802
N	2.898203	-2.734927	-0.687849
O	3.483988	2.736783	-0.424115
C	2.576283	3.576771	0.261339
H	2.550809	3.346261	1.331972
H	2.940007	4.592354	0.120006
H	1.565755	3.491168	-0.153821
C	-0.156774	-0.607939	1.555415
H	0.188942	0.395919	1.831727
N	-0.548281	-1.386120	2.726132
H	-1.403978	-0.975746	3.086908
H	0.161459	-1.322709	3.446309
C	-1.405446	-0.462111	0.666579
H	-1.750516	-1.486438	0.487182
C	-1.176030	0.241008	-0.692778
C	-3.754424	-0.082082	0.722169
C	-2.294298	1.649345	1.468323
C	-2.386551	1.144265	-0.969114
H	-0.276462	0.862056	-0.676872
H	-1.029250	-0.496314	-1.485048
C	-3.710067	0.367426	-0.776115
H	-3.959286	-1.153712	0.795905
H	-4.556213	0.437944	1.252785
C	-2.355709	2.286406	0.054003
H	-1.334024	1.850790	1.947917
H	-3.063110	2.066428	2.123248
H	-2.341177	1.538836	-1.986590
H	-4.533811	1.062445	-0.970144
H	-1.484703	2.923882	-0.123975
H	-3.245588	2.913469	-0.055997
N	-2.493867	0.195745	1.412733
C	-3.848303	-0.789760	-1.720790
H	-3.144460	-1.610777	-1.585558
C	-4.758432	-0.878661	-2.683143
H	-5.484174	-0.086937	-2.848889
H	-4.812201	-1.742077	-3.337157

9. 9-ADEQ_syn-closed_internal_OMe
Imaginary Frequencies = 0 (Equilibrium Geometry)
Energy (Hartree) = -1016.55278372

C	5.007272	0.289166	0.532118
C	4.453732	1.524843	0.399374
C	3.066915	1.684130	0.133492

C	2.253334	0.528500	0.012614
C	2.853396	-0.753808	0.156005
C	4.197801	-0.869724	0.409020
H	6.063422	0.156685	0.733566
H	5.051765	2.423617	0.490275
C	0.862513	0.720881	-0.255359
H	2.243282	-1.641754	0.073622
C	0.415500	2.013035	-0.383919
C	1.313907	3.089411	-0.242639
H	-0.625946	2.201003	-0.614290
H	0.946187	4.107654	-0.344550
N	2.592401	2.952003	0.007217
O	4.860216	-2.038576	0.561548
C	4.123336	-3.240834	0.454080
H	3.670002	-3.341809	-0.538104
H	4.838202	-4.046502	0.607250
H	3.341911	-3.298380	1.219573
C	-0.057784	-0.476404	-0.453776
H	0.265715	-1.258524	0.240881
N	0.069492	-1.045519	-1.801690
H	0.025704	-0.292370	-2.480838
H	0.989184	-1.458281	-1.911341
C	-1.518658	-0.225727	-0.039792
H	-1.442133	0.324065	0.904264
C	-2.287432	-1.537947	0.230219
C	-3.420584	1.179485	-0.126173
C	-2.887455	-0.088966	-2.067167
C	-3.772509	-1.292349	-0.080074
H	-1.911174	-2.332508	-0.422501
H	-2.136046	-1.861706	1.263690
C	-4.253814	0.037529	0.548935
H	-3.013178	1.857172	0.630390
H	-4.059483	1.775210	-0.783216
C	-3.912139	-1.155754	-1.600118
H	-2.078809	-0.565095	-2.614704
H	-3.353999	0.643362	-2.731008
H	-4.385223	-2.115945	0.293415
H	-5.309363	0.163251	0.285902
H	-3.713856	-2.115536	-2.085052
H	-4.935555	-0.864898	-1.857303
N	-2.313374	0.648035	-0.927525
C	-4.150554	0.053126	2.045634
H	-3.146315	0.005963	2.465948
C	-5.184974	0.128197	2.874655
H	-6.204325	0.183301	2.501958
H	-5.052162	0.138271	3.950993

10. 9-ADEQ_syn-open_internal_OMe
Imaginary Frequencies = 0 (Equilibrium Geometry)
Energy (Hartree) = -1016.56073193

C	-3.932795	1.237380	-0.764376
C	-3.969146	-0.036696	-1.241642
C	-3.005496	-0.999242	-0.836856

C	-1.989508	-0.610602	0.076113
C	-1.986310	0.717476	0.583254
C	-2.931716	1.619365	0.165707
H	-4.662805	1.978638	-1.067075
H	-4.731034	-0.355522	-1.942732
C	-1.008180	-1.589241	0.423706
H	-1.259754	0.964252	1.341945
C	-1.154703	-2.845915	-0.108741
C	-2.218865	-3.133330	-0.986401
H	-0.441266	-3.627514	0.129105
H	-2.318556	-4.133693	-1.400508
N	-3.111983	-2.251329	-1.357657
O	-3.009136	2.902783	0.587887
C	-2.031101	3.351695	1.506523
H	-1.023775	3.271275	1.082978
H	-2.260143	4.397112	1.703149
H	-2.074540	2.785823	2.443044
C	0.216969	-1.290276	1.269771
H	0.731253	-2.254500	1.405762
N	-0.114650	-0.661859	2.543308
H	0.756924	-0.361926	2.968721
H	-0.565893	-1.324436	3.161793
C	1.182857	-0.363253	0.509537
H	0.660309	0.586732	0.368955
C	1.619878	-0.904093	-0.869953
C	2.997056	1.147419	0.768225
C	3.327750	-1.145175	1.335870
C	3.098746	-0.541136	-1.069278
H	1.512448	-1.993462	-0.916254
H	0.987798	-0.494721	-1.661233
C	3.344354	0.952181	-0.745628
H	2.323948	1.999275	0.900857
H	3.902676	1.357545	1.343173
C	3.920410	-1.376944	-0.079561
H	2.829064	-2.044417	1.703697
H	4.111115	-0.903998	2.058471
H	3.407695	-0.746981	-2.096415
H	4.409375	1.154363	-0.900332
H	3.873695	-2.434983	-0.351879
H	4.971992	-1.076903	-0.119015
N	2.358901	-0.041117	1.337634
C	2.563968	1.877962	-1.632011
H	1.479343	1.849661	-1.527970
C	3.100022	2.718130	-2.509060
H	4.176532	2.784860	-2.642061
H	2.485683	3.367155	-3.123443

11. Bridge_N-Boc-L-Phenylglycine

N	-8.811343	0.284145	-4.728964
C	-8.217143	0.023945	-6.065164
C	-6.682243	-0.185055	-6.110364
O	-6.274143	-1.111455	-5.206464
H	-9.695043	-0.140855	-4.506064
H	-8.414143	0.927945	-6.645964
C	-8.931143	-1.128655	-6.750564
C	-10.250843	-3.202255	-8.081464
C	-9.010643	-1.139455	-8.149464
C	-9.507443	-2.186355	-6.028464
C	-10.172543	-3.214655	-6.691464
C	-9.668143	-2.171555	-8.813764
H	-8.563043	-0.343055	-8.727664
H	-9.433743	-2.225255	-4.951664
H	-10.620843	-4.022655	-6.130664
H	-9.730443	-2.169855	-9.892864
H	-10.766443	-4.000455	-8.597164
O	-5.950843	0.391145	-6.884964
C	-8.381143	1.298345	-3.894664
O	-9.217843	1.344745	-2.839364
O	-7.437043	2.060845	-4.092364
C	-9.222943	2.367645	-1.837164
C	-10.411743	1.994345	-0.938464
H	-11.346143	1.981945	-1.501064
H	-10.533143	2.700045	-0.116264
H	-10.288043	1.001245	-0.505264
C	-7.929243	2.346945	-1.000464
H	-7.977343	3.060645	-0.178064
H	-7.056243	2.598045	-1.600864
H	-7.758743	1.359245	-0.573364
C	-9.471743	3.765445	-2.441864
H	-10.361043	3.767945	-3.072564
H	-8.638443	4.096045	-3.061464
H	-9.616443	4.516845	-1.665464
H	-5.319043	-1.221355	-5.335764

12. Complete_Catalytic_Salt
Imaginary Frequencies = 0 (Equilibrium Geometry)
Energy (Hartree) = -2739.2008769

C	6.416223	-3.800768	0.198133
C	5.665467	-4.782614	0.762500
C	4.300268	-4.562554	1.092254
C	3.727166	-3.287125	0.834168
C	4.533675	-2.268180	0.251610
C	5.845229	-2.526686	-0.068593
H	7.455501	-3.958503	-0.063704
H	6.078936	-5.761340	0.973161
C	2.347892	-3.120202	1.161728
H	4.117837	-1.291569	0.037056
C	1.685295	-4.184988	1.724171
C	2.356902	-5.401789	1.942719
H	0.643736	-4.100371	2.009534
H	1.822226	-6.238576	2.384058
N	3.615609	-5.599043	1.639343
O	6.685779	-1.648158	-0.642743
C	6.181050	-0.366467	-0.995816
H	5.828006	0.176654	-0.114444
H	7.017161	0.165617	-1.444412
H	5.360105	-0.440887	-1.714858

C	1.625621	-1.806922	0.939645
H	2.361986	-1.004543	0.874179
N	0.721327	-1.535589	2.078869
H	-0.895727	-2.017255	2.112709
C	0.756352	-1.697580	-0.343784
H	0.137191	-0.813763	-0.171317
C	-0.147785	-2.879046	-0.728398
C	0.663610	-0.647855	-2.536346
C	2.216670	-2.501173	-2.218460
C	-0.233374	-2.958129	-2.260498
H	0.254017	-3.821461	-0.351286
H	-1.133820	-2.751155	-0.284588
C	-0.534664	-1.570600	-2.869763
H	0.345076	0.289045	-2.080340
H	1.274603	-0.405805	-3.403613
C	1.126834	-3.422295	-2.794549
H	2.830737	-3.005275	-1.478499
H	2.878428	-2.086648	-2.978105
H	-1.015836	-3.659395	-2.552696
H	-0.584247	-1.692502	-3.955177
H	1.319775	-4.453644	-2.493943
H	1.131777	-3.390227	-3.886313
N	1.566251	-1.332230	-1.555349
C	-1.838488	-0.986868	-2.403599
H	-1.880031	-0.650653	-1.369628
C	-2.910495	-0.841038	-3.170750
H	-2.905988	-1.142658	-4.214783
H	-3.821048	-0.400349	-2.780981
N	-4.287304	-0.239636	0.253293
C	-3.927290	-1.172049	1.287835
C	-2.417183	-1.086397	1.469221
O	-1.776809	-0.121244	1.101075
H	-3.628819	0.484332	0.011758
H	-4.178044	-2.186742	0.970166
C	-4.594568	-0.904048	2.633317
C	-5.746822	-0.419935	5.124708
C	-4.862871	0.399637	3.039043
C	-4.901965	-1.963793	3.480986
C	-5.477036	-1.723204	4.722460
C	-5.439306	0.640561	4.280040
H	-4.633556	1.226581	2.376289
H	-4.695807	-2.981049	3.165860
H	-5.721420	-2.555043	5.373108
H	-5.650425	1.658675	4.586629
H	-6.200069	-0.231870	6.091217
O	-1.900894	-2.139992	2.055783
C	-5.469168	-0.332795	-0.407896
O	-5.531905	0.628571	-1.343683
O	-6.311575	-1.171863	-0.176761
C	-6.716210	0.803139	-2.184461
C	-6.324026	1.980774	-3.068259
H	-6.110434	2.862325	-2.459811
H	-7.140474	2.219077	-3.753550
H	-5.435537	1.738689	-3.656509
C	-6.959489	-0.444304	-3.029140
H	-7.776176	-0.249086	-3.728679
H	-7.225827	-1.295591	-2.404981
H	-6.067507	-0.692568	-3.609941
C	-7.919565	1.153959	-1.315293
H	-7.691433	2.018777	-0.687176
H	-8.201211	0.315967	-0.678843
H	-8.766995	1.409369	-1.956250
H	0.492944	-0.544027	2.077866
H	2.299033	-0.633772	-1.239761
N	3.105969	3.221112	-0.099410
H	3.244900	2.484832	0.575319
C	2.715286	2.808717	-1.424393
C	3.136618	1.336793	-1.629839
O	3.383942	0.940653	-2.769656
O	3.077066	0.613905	-0.582131
C	1.209271	2.827719	-1.655022
C	0.328380	2.556884	-0.610118
C	-1.036852	2.445610	-0.846558
C	-1.536849	2.596610	-2.134851
C	-0.663037	2.871042	-3.181830
C	0.701489	2.985814	-2.942352
C	2.995608	4.505458	0.315520
O	2.715644	5.437660	-0.408709
O	3.255559	4.578014	1.634822
C	3.218240	5.852423	2.342666
C	3.569697	5.453683	3.771349
C	4.270146	6.803866	1.778684
C	1.813338	6.446779	2.283404
H	4.025530	7.095596	0.758515
H	4.319847	7.700233	2.402333
H	5.252863	6.325179	1.787018
H	4.558681	4.991114	3.806551
H	3.573992	6.336566	4.414710
H	2.837680	4.742130	4.160385
H	1.553774	6.735706	1.266167
H	1.081887	5.718836	2.644350
H	1.765196	7.329388	2.926477
H	3.207821	3.430969	-2.171863
H	1.383234	3.184734	-3.762214
H	-1.044987	2.993654	-4.189198
H	-2.599832	2.494350	-2.322022
H	-1.701322	2.216778	-0.022145
H	0.714675	2.428945	0.394695
H	1.214319	-1.720842	2.946764

13. E-dienamine_anti-open_external_OMe

Imaginary Frequencies = 0 (Equilibrium Geometry)

Energy (Hartree) = -1327.40192498

C	-1.400417	4.284186	0.194768
C	-1.298377	3.887989	1.500038
C	-0.859241	2.590026	1.848070
C	-0.504055	1.683910	0.809626
C	-0.625232	2.111155	-0.532108

C	-1.062729	3.378338	-0.839468
H	-1.743348	5.284429	-0.033773
H	-1.558031	4.560988	2.308294
C	-0.067882	0.381094	1.199990
H	-0.386911	1.454807	-1.358307
C	-0.047404	0.088710	2.536660
C	-0.421690	1.063988	3.483913
H	0.242667	-0.901941	2.866312
H	-0.394447	0.819406	4.542915
N	-0.807652	2.274349	3.171625
O	-1.142647	3.682633	-2.157251
C	-1.611064	4.964621	-2.532152
H	-0.954430	5.757324	-2.158745
H	-1.602384	4.977234	-3.620155
H	-2.632956	5.137455	-2.178986
C	0.393495	-0.672099	0.203623
H	0.059554	-0.384608	-0.801714
N	-0.116921	-1.985297	0.529014
H	0.570504	-2.695398	0.299961
C	1.932834	-0.745138	0.219276
H	2.221285	-0.946274	1.256782
C	2.619840	0.557234	-0.260333
C	3.816859	-2.132245	-0.223508
C	2.280189	-1.672710	-1.993723
C	3.769569	0.166350	-1.198959
H	1.922279	1.195633	-0.809271
H	2.972995	1.143108	0.590833
C	4.695759	-0.874987	-0.528578
H	3.885108	-2.400875	0.834512
H	4.166181	-2.993020	-0.798971
C	3.156394	-0.475269	-2.450625
H	1.226248	-1.505979	-2.226260
H	2.571275	-2.593535	-2.504440
H	4.352115	1.049680	-1.469534
H	5.471833	-1.142127	-1.253392
H	2.555843	0.262829	-2.989488
H	3.949012	-0.806806	-3.127721
N	2.408182	-1.906589	-0.549779
C	5.371590	-0.347987	0.702445
H	4.723849	-0.124488	1.550093
C	6.678654	-0.151161	0.825422
H	7.361605	-0.368196	0.008495
H	7.112356	0.231714	1.742690
C	-1.423017	-2.333151	0.221348
C	-3.173808	-4.110530	0.488082
C	-3.770814	-1.839696	-0.404318
C	-4.027749	-3.326544	-0.508475
C	-2.396568	-1.450611	-0.081875
C	-1.691563	-3.815344	0.284903
H	-3.360562	-5.182812	0.384736
H	-5.083138	-3.555630	-0.352003
H	-2.185328	-0.386772	-0.090654
H	-1.099858	-4.248404	1.097542
H	-3.459668	-3.827851	1.506973
H	-3.778104	-3.658135	-1.525408
H	-1.337883	-4.282096	-0.644202
C	-4.703209	-0.900538	-0.625622
H	-4.388884	0.138980	-0.539216
C	-6.140310	-1.099348	-1.002563
H	-6.805353	-0.613543	-0.279959
H	-6.425254	-2.150689	-1.064365
H	-6.352586	-0.644172	-1.976727

14. E-dienamine_anti-open_internal_OMe
Imaginary Frequencies = 0 (Equilibrium Geometry)
Energy (Hartree) = -1327.40475103

C	-1.269519	4.404236	-0.015274
C	-1.167620	4.105907	1.307567
C	-0.769922	2.811563	1.741330
C	-0.460616	1.825996	0.766448
C	-0.582290	2.158986	-0.611957
C	-0.979706	3.415712	-0.992148
H	-1.576457	5.385824	-0.355641
H	-1.391401	4.841433	2.070738
C	-0.066700	0.541804	1.239082
H	-0.369506	1.411550	-1.361097
C	-0.040184	0.338376	2.595354
C	-0.368022	1.385813	3.473906
H	0.219720	-0.637384	2.988417
H	-0.338501	1.216310	4.547252
N	-0.714542	2.587333	3.078980
O	-1.131057	3.824062	-2.271670
C	-0.877142	2.887605	-3.301109
H	-1.548505	2.025204	-3.227497
H	-1.062421	3.411284	-4.236503
H	0.162974	2.543818	-3.277275
C	0.348252	-0.594229	0.315275
H	0.030353	-0.359127	-0.708462
N	-0.226868	-1.858493	0.717553
H	0.430999	-2.613132	0.553996
C	1.882092	-0.734787	0.338264
H	2.165897	-0.881541	1.385937
C	2.620938	0.505540	-0.225234
C	3.710938	-2.210894	-0.046121
C	2.146363	-1.832202	-1.809714
C	3.710622	0.012518	-1.186190
H	1.938378	1.158957	-0.775498
H	3.038932	1.106759	0.584730
C	4.625837	-1.024019	-0.493454
H	3.799445	-2.378623	1.031050
H	4.012682	-3.136086	-0.543087
C	3.017181	-0.674972	-2.369888
H	1.088514	-1.681369	-2.035565
H	2.427150	-2.788933	-2.256174
H	4.313936	0.853717	-1.534556
H	5.343827	-1.380524	-1.239475
H	2.400668	0.051056	-2.908072
H	3.763294	-1.053192	-3.074545

N	2.302448	-1.962849	-0.355668
C	5.397879	-0.445941	0.655058
H	4.812169	-0.124856	1.516404
C	6.718294	-0.314172	0.692458
H	7.341000	-0.628949	-0.140628
H	7.223284	0.110489	1.553082
C	-1.539462	-2.166517	0.394492
C	-3.380229	-3.841260	0.711739
C	-3.841127	-1.606331	-0.338129
C	-4.165196	-3.083612	-0.359186
C	-2.460996	-1.260844	0.007714
C	-1.879966	-3.627744	0.540406
H	-3.614716	-4.908222	0.668578
H	-5.234564	-3.253724	-0.224108
H	-2.200418	-0.209821	-0.054866
H	-1.334771	-4.034960	1.397707
H	-3.683114	-3.481988	1.701104
H	-3.900941	-3.489895	-1.344879
H	-1.520607	-4.168244	-0.345571
C	-4.719479	-0.641335	-0.650710
H	-4.359159	0.386313	-0.616017
C	-6.151438	-0.797890	-1.065089
H	-6.815886	-0.241902	-0.394456
H	-6.482232	-1.837489	-1.076974
H	-6.311522	-0.391023	-2.070069

15. E-dienamine_syn-open_external_OMe
Imaginary Frequencies = 0 (Equilibrium Geometry)
Energy (Hartree) = -1327.40133736

C	0.210548	4.411661	-0.033241
C	0.411877	4.087388	1.280826
C	0.405824	2.744490	1.720046
C	0.184382	1.710165	0.767235
C	-0.013152	2.065834	-0.585449
C	-0.003584	3.383143	-0.981157
H	0.222427	5.450982	-0.333684
H	0.582514	4.856067	2.024871
C	0.168735	0.364414	1.254383
H	-0.139493	1.300860	-1.337447
C	0.386979	0.174091	2.592451
C	0.604279	1.274117	3.450450
H	0.398924	-0.827964	3.007852
H	0.775083	1.100851	4.510096
N	0.612089	2.516861	3.048420
O	-0.201709	3.613554	-2.302759
C	-0.184124	4.949076	-2.769055
H	0.784369	5.426299	-2.585876
H	-0.356081	4.891645	-3.842066
H	-0.979230	5.546151	-2.310019
C	-0.154692	-0.842483	0.386320
H	0.077576	-1.731326	0.995937
N	0.564726	-0.896500	-0.863057
H	-0.010569	-1.311874	-1.587450
C	-1.666697	-0.855672	0.069430
H	-1.904955	0.105557	-0.395467
C	-2.550044	-1.044446	1.325555
C	-3.322334	-1.601277	-1.468761
C	-1.944257	-3.221397	-0.379147
C	-3.672826	-2.028119	0.967159
H	-1.968264	-1.458427	2.155596
H	-2.944455	-0.085785	1.668540
C	-4.393155	-1.586445	-0.328810
H	-3.304059	-0.641101	-1.991963
H	-3.560665	-2.369190	-2.208758
C	-3.033244	-3.399345	0.712253
H	-0.944954	-3.399797	0.023962
H	-2.083691	-3.931522	-1.197525
H	-4.397091	-2.089206	1.782331
H	-5.165646	-2.330923	-0.547642
H	-2.596734	-3.785155	1.637616
H	-3.796820	-4.113537	0.390319
N	-1.979719	-1.869190	-0.950781
C	-5.060486	-0.249065	-0.198186
H	-4.401127	0.610164	-0.075864
C	-6.372506	-0.049850	-0.231637
H	-7.066163	-0.876452	-0.360119
H	-6.799698	0.942052	-0.133166
C	1.914499	-1.183162	-0.933764
C	3.759228	-2.093750	-2.369798
C	4.190329	-1.440984	0.016062
C	4.744574	-1.455666	-1.391027
C	2.742887	-1.251639	0.129003
C	2.421149	-1.363812	-2.342032
H	3.605424	-3.142631	-2.093645
H	4.935247	-0.420983	-1.705937
H	2.349765	-1.126228	1.131257
H	2.516853	-0.376381	-2.810757
H	4.170768	-2.082544	-3.382589
H	5.700824	-1.979709	-1.432441
H	1.675495	-1.912162	-2.928731
C	4.946212	-1.553407	1.119163
H	4.432546	-1.504333	2.078668
C	6.433990	-1.715771	1.205077
H	6.887265	-0.867378	1.730454
H	6.913898	-1.788123	0.227963
H	6.698378	-2.613070	1.775586

16. E-dienamine_syn-open_internal_OMe
Imaginary Frequencies = 0 (Equilibrium Geometry)
Energy (Hartree) = -1327.40481452

C	-0.249906	4.445187	0.393077
C	-0.439409	4.247581	-0.939752
C	-0.425684	2.940623	-1.497706
C	-0.207615	1.829883	-0.639613
C	-0.022779	2.058926	0.751477
C	-0.042386	3.335499	1.252498

H	-0.259798	5.437461	0.827959
H	-0.606417	5.077597	-1.615547
C	-0.179016	0.535551	-1.239098
H	0.089874	1.204787	1.400171
C	-0.387864	0.458914	-2.593127
C	-0.605054	1.625520	-3.350639
H	-0.389636	-0.503721	-3.093226
H	-0.768366	1.547602	-4.422536
N	-0.620583	2.830391	-2.838454
O	0.119042	3.650657	2.558275
C	0.320071	2.588789	3.470894
H	1.229574	2.026606	3.231738
H	0.426182	3.049742	4.450754
H	-0.535689	1.905053	3.479756
C	0.153872	-0.739686	-0.477792
H	-0.072284	-1.575314	-1.160318
N	-0.559664	-0.906664	0.765963
H	0.017007	-1.397494	1.440793
C	1.666445	-0.763683	-0.164188
H	1.892898	0.158339	0.379915
C	2.551528	-0.834679	-1.430537
C	3.330200	-1.609274	1.311538
C	1.983841	-3.154122	0.081196
C	3.694454	-1.820409	-1.150290
H	1.977210	-1.193888	-2.290748
H	2.925422	0.155978	-1.696839
C	4.402301	-1.472630	0.180836
H	3.293173	-0.699734	1.918014
H	3.582061	-2.435325	1.981121
C	3.083730	-3.221076	-1.011540
H	0.990666	-3.311071	-0.345368
H	2.127328	-3.930300	0.836425
H	4.421650	-1.798608	-1.964758
H	5.191690	-2.214626	0.339284
H	2.661825	-3.541134	-1.968151
H	3.860606	-3.942265	-0.741153
N	1.993609	-1.855288	0.767406
C	5.038508	-0.113833	0.166084
H	4.360344	0.737553	0.109115
C	6.345271	0.111832	0.226122
H	7.057297	-0.706507	0.291263
H	6.749665	1.117959	0.212810
C	-1.912439	-1.188773	0.818371
C	-3.760416	-2.196521	2.184080
C	-4.194420	-1.344509	-0.137501
C	-4.742663	-1.468940	1.266443
C	-2.745499	-1.160247	-0.241772
C	-2.414150	-1.481544	2.209449
H	-3.619462	-3.220537	1.821409
H	-4.920275	-0.461712	1.666489
H	-2.355158	-0.956270	-1.232172
H	-2.497137	-0.535534	2.759661
H	-4.167382	-2.264712	3.196462
H	-5.704636	-1.983976	1.269655
H	-1.671907	-2.085047	2.744220
C	-4.955307	-1.359312	-1.242615
H	-4.444809	-1.236381	-2.197128
C	-6.444745	-1.500377	-1.334663
H	-6.891803	-0.606306	-1.783993
H	-6.921318	-1.651198	-0.364921
H	-6.719459	-2.343482	-1.978146

17. E-iPr-EtMe-Dienamine
Imaginary Frequencies = 0 (Equilibrium Geometry)
Energy (Hartree) = -563.954683361

C	1.598632	1.589022	-0.395822
C	1.238932	0.119586	-0.316606
C	-0.196471	-0.159592	-0.228664
C	-1.140667	0.797697	-0.082504
C	-0.774378	2.250327	0.090014
C	0.683114	2.423860	0.498580
C	2.169805	-0.854107	-0.356979
C	3.647293	-0.575802	-0.531008
N	-2.500836	0.572501	-0.146926
C	-3.100773	-0.752704	-0.144866
C	-4.553696	-0.633847	-0.592854
C	-2.988839	-1.449310	1.214931
H	1.491189	1.935263	-1.433194
H	2.636865	1.759127	-0.109128
H	-0.513750	-1.188299	-0.337216
H	-1.435748	2.705609	0.836432
H	-0.967475	2.774362	-0.854515
H	0.810687	2.104036	1.538602
H	0.961411	3.480046	0.447981
C	1.836148	-2.321628	-0.233058
H	4.085173	-1.388616	-1.122375
H	3.815081	0.336260	-1.108809
C	4.394184	-0.478593	0.804859
H	-3.065698	1.282415	0.295396
H	-2.563600	-1.346907	-0.891670
H	-5.023251	-1.618948	-0.639060
H	-5.126111	-0.024538	0.115545
H	-4.618622	-0.167754	-1.578229
H	-3.378040	-2.469848	1.162295
H	-1.950166	-1.493702	1.548906
H	-3.567110	-0.901134	1.965770
H	2.652043	-2.853613	0.266527
H	0.928018	-2.510224	0.341310
H	1.713253	-2.788345	-1.218720
H	5.468822	-0.343199	0.652622
H	4.022635	0.365594	1.392758
H	4.250548	-1.384551	1.401128

18. E-iPr-HMe-Dienamine
Imaginary Frequencies = 0 (Equilibrium Geometry)
Energy (Hartree) = -485.3287553

C	-2.351243	1.029065	-0.191383
C	-1.770592	-0.365941	-0.126323
C	-0.309220	-0.448744	-0.128126
C	0.503476	0.630542	-0.101740
C	-0.055970	2.027783	0.008501
C	-1.475686	2.028300	0.563339
C	-2.515120	-1.482698	-0.100169
C	-4.009903	-1.596098	-0.127261
N	1.876195	0.588190	-0.238569
C	2.651990	-0.641742	-0.198070
C	2.721108	-1.249434	1.206399
C	4.044304	-0.362232	-0.754309
H	-3.367136	1.051964	0.206110
H	-2.414191	1.338244	-1.243357
H	0.115514	-1.446069	-0.175745
H	-0.036813	2.495396	-0.983887
H	0.599176	2.631376	0.647087
H	-1.902381	3.032683	0.496695
H	-1.450382	1.755977	1.624007
H	-1.982689	-2.432847	-0.073101
H	-4.342248	-2.145343	-1.015668
H	-4.377130	-2.155711	0.740325
H	-4.512870	-0.627961	-0.136323
H	2.359928	1.393471	0.130668
H	2.156240	-1.352778	-0.867189
H	3.244940	-2.209173	1.189663
H	3.262021	-0.576607	1.879805
H	1.721159	-1.410116	1.614376
H	4.640983	-1.276929	-0.773817
H	3.983174	0.038583	-1.768216
H	4.570480	0.365693	-0.126602

19. EG1-anti
Imaginary Frequencies = 0 (Equilibrium Geometry)
Energy (Hartree) = -3797.76846359

C	1.775110	7.161702	-1.120222
C	0.426195	7.312298	-1.169513
C	-0.440910	6.198869	-0.998247
C	0.125308	4.915994	-0.757525
C	1.542894	4.782721	-0.691573
C	2.345945	5.881146	-0.886018
H	2.447331	7.999620	-1.260105
H	-0.029872	8.278211	-1.349503
C	-0.782555	3.827309	-0.615261
H	1.995582	3.819268	-0.492089
C	-2.125257	4.085380	-0.748350
C	-2.575899	5.397116	-0.970880
H	-2.845003	3.276456	-0.701762
H	-3.641388	5.593627	-1.057893
N	-1.774884	6.428495	-1.088321
O	3.691542	5.868744	-0.883120
C	4.347290	4.630776	-0.648170
H	4.067598	3.888277	-1.402801
H	5.412261	4.841401	-0.722018
H	4.112598	4.246379	0.347586
C	-0.320975	2.397768	-0.377557
H	0.740196	2.328384	-0.605215
N	-1.007728	1.479291	-1.261108
H	-1.791561	0.962893	-0.879066
C	-0.432909	1.862628	1.072696
H	-0.322751	0.785052	0.953187
C	-1.691005	2.160334	1.896880
C	0.942183	1.224127	2.977014
C	0.618638	3.606076	2.530709
C	-1.293288	2.245753	3.378198
H	-2.144828	3.108594	1.600412
H	-2.426500	1.377024	1.719185
C	-0.378865	1.061234	3.768722
H	1.251485	0.299157	2.488616
H	1.767504	1.564813	3.600018
C	-0.501745	3.544728	3.584163
H	0.397368	4.313160	1.734569
H	1.597547	3.850947	2.939629
H	-2.185369	2.234079	4.005523
H	-0.150722	1.153693	4.834043
H	-1.157352	4.410132	3.472755
H	-0.084200	3.574806	4.593382
N	0.756090	2.256232	1.906611
C	-1.028772	-0.275520	3.541717
H	-1.167999	-0.582968	2.508969
C	-1.439428	-1.083976	4.509641
H	-1.313458	-0.827133	5.557765
H	-1.920571	-2.028567	4.283176
C	-0.397247	0.917046	-2.357006
C	-0.524401	-0.906457	-4.107006
C	1.466262	0.564155	-3.938399
C	0.493050	-0.081535	-4.892777
C	0.861161	1.195846	-2.773035
C	-1.289502	-0.040042	-3.109080
H	0.019836	-1.693933	-3.576966
H	-0.036453	0.697589	-5.456062
H	1.476413	1.886032	-2.211778
H	-2.049278	0.552799	-3.632409
H	-1.228461	-1.403516	-4.780174
H	1.020953	-0.709404	-5.614390
H	-1.839036	-0.660236	-2.391428
C	2.791512	0.511853	-4.149401
C	-0.805016	-4.754544	0.042157
C	-2.799163	-4.430654	-1.965261
C	-0.663834	-3.828279	-1.004300
C	-1.998785	-5.488394	0.037036
C	-2.977819	-5.336378	-0.931563
C	-1.637217	-3.683272	-1.990964
H	-2.179804	-6.206285	0.824217
H	-3.882917	-5.929766	-0.873389
H	-1.471238	-2.961687	-2.779208
H	-3.555103	-4.292506	-2.727293
C	0.183174	-4.966194	1.208694

C	1.658225	-4.739697	0.825736
H	1.915767	-5.264920	-0.097227
H	2.299867	-5.129647	1.619787
H	1.914746	-3.687409	0.710231
C	-0.216288	-4.016420	2.354384
H	-1.267906	-4.154655	2.619696
H	-0.071925	-2.968303	2.085426
H	0.386472	-4.223329	3.244109
C	0.094925	-6.413497	1.734737
H	0.249650	-7.139929	0.932383
H	-0.858511	-6.629445	2.219817
H	0.872504	-6.566825	2.486732
N	0.445113	-2.926327	-1.100972
C	0.507477	-1.715750	-0.458163
C	1.597666	-3.089114	-1.905587
C	1.784799	-1.064266	-0.836923
H	2.085044	-0.103518	-0.435627
C	2.426699	-1.870060	-1.678614
H	3.391427	-1.723920	-2.137828
O	1.821290	-4.039697	-2.600907
O	-0.338853	-1.282352	0.305468
N	4.882964	-0.029536	0.113531
C	4.752882	0.620179	1.393035
C	3.755901	1.798039	1.263944
O	2.897288	1.709510	0.328802
H	4.287753	0.303837	-0.627912
H	5.714999	1.020963	1.719979
C	4.184768	-0.265216	2.497139
C	2.936370	-1.696090	4.552391
C	3.486795	-1.435445	2.218495
C	4.278689	0.165559	3.820332
C	3.654758	-0.539917	4.840295
C	2.868043	-2.146787	3.240672
H	3.425689	-1.793144	1.197519
H	4.816777	1.080171	4.040851
H	3.730288	-0.186367	5.862675
H	2.331132	-3.057316	3.008877
H	2.445238	-2.248385	5.345418
O	3.797577	2.683531	2.121479
C	5.758191	-1.031828	-0.129899
O	5.634033	-1.434258	-1.414195
O	6.524083	-1.492637	0.687965
C	6.312831	-2.630816	-1.906509
C	5.845840	-2.719515	-3.355809
H	4.768557	-2.892129	-3.415686
H	6.347151	-3.552796	-3.853119
H	6.086492	-1.798911	-3.892522
C	7.825963	-2.445073	-1.846367
H	8.313567	-3.293141	-2.333907
H	8.172347	-2.380740	-0.816554
H	8.112578	-1.533282	-2.376697
C	5.842103	-3.849630	-1.117381
H	4.751927	-3.928105	-1.157893
H	6.158091	-3.789171	-0.076533
H	6.263570	-4.754651	-1.561793
N	-5.963017	-0.568866	0.140119
C	-5.032637	-1.223124	1.026950
C	-3.596267	-1.055344	0.523763
O	-3.227457	-0.074610	-0.085078
H	-6.365528	0.311678	0.414646
H	-5.241916	-2.293059	1.073646
C	-5.072916	-0.631911	2.423764
C	-5.010834	0.447381	4.996191
C	-4.956905	0.746357	2.607746
C	-5.150367	-1.463506	3.536381
C	-5.118200	-0.926224	4.817651
C	-4.933203	1.283419	3.887932
H	-4.867592	1.403147	1.748465
H	-5.232164	-2.536084	3.399363
H	-5.178740	-1.583155	5.677497
H	-4.848314	2.356215	4.018891
H	-4.987462	0.865986	5.995578
O	-2.823577	-2.048773	0.898221
H	-1.895219	-1.869338	0.614303
C	-5.889441	-0.837271	-1.195362
O	-6.587426	0.065342	-1.889084
O	-5.273172	-1.780028	-1.646288
C	-6.538090	0.091748	-3.353013
C	-7.381581	1.313659	-3.693446
H	-8.397906	1.195331	-3.311211
H	-7.429296	1.440857	-4.777156
H	-6.945428	2.214284	-3.255660
C	-5.096972	0.286008	-3.818449
H	-5.086568	0.460131	-4.897212
H	-4.488593	-0.589970	-3.597181
H	-4.655616	1.156433	-3.325649
C	-7.167373	-1.174918	-3.923701
H	-8.175939	-1.307343	-3.524100
H	-6.570568	-2.052878	-3.681614
H	-7.238485	-1.083826	-5.010521
H	1.603444	2.225593	1.289796
C	3.866158	1.080005	-3.269293
H	3.475611	1.474816	-2.327883
H	4.613944	0.316069	-3.027728
H	4.401800	1.894357	-3.770354
H	3.139193	0.012936	-5.051964

20. EG10-anti

Imaginary Frequencies = 0 (Equilibrium Geometry)

Energy (Hartree) = -3876.39184219

C	4.035836	-5.142040	-1.336595
C	2.926401	-5.810835	-0.925244
C	1.717305	-5.119380	-0.636934
C	1.678362	-3.708695	-0.802361
C	2.852869	-3.026768	-1.234524
C	4.005160	-3.729131	-1.486458
H	4.964436	-5.657736	-1.549554
H	2.930878	-6.886256	-0.795122

C	0.454443	-3.052197	-0.491802
H	2.846329	-1.950907	-1.355412
C	-0.587109	-3.816458	-0.026784
C	-0.441200	-5.208522	0.095230
H	-1.527219	-3.352071	0.251886
H	-1.274607	-5.807829	0.453086
N	0.662093	-5.852979	-0.202075
O	5.173107	-3.179608	-1.874526
C	5.235639	-1.766574	-1.991230
H	5.039865	-1.285840	-1.028897
H	6.250143	-1.536538	-2.310051
H	4.524470	-1.395386	-2.735645
C	0.285818	-1.543687	-0.612866
H	1.270747	-1.090207	-0.704642
N	-0.298442	-0.957098	0.577120
H	-1.308986	-0.923065	0.617736
C	-0.513185	-1.048897	-1.852466
H	-0.974952	-0.118484	-1.521815
C	-1.586721	-1.946612	-2.485228
C	-0.277618	0.405236	-3.789090
C	0.857951	-1.756542	-3.818119
C	-1.619030	-1.688710	-4.000494
H	-1.371378	-3.003471	-2.316859
H	-2.543364	-1.730731	-2.014324
C	-1.619794	-0.173722	-4.309661
H	-0.429392	1.280940	-3.156495
H	0.412064	0.674118	-4.587833
C	-0.347501	-2.292941	-4.608684
H	1.290146	-2.511761	-3.164898
H	1.653740	-1.362750	-4.450247
H	-2.509339	-2.144073	-4.436668
H	-1.649343	-0.057078	-5.396592
H	-0.375163	-3.382331	-4.545948
H	-0.269061	-2.024483	-5.664851
N	0.412962	-0.622290	-2.957636
C	-2.810652	0.528921	-3.725515
H	-2.839500	0.604615	-2.643271
C	-3.808577	1.041804	-4.431092
H	-3.819583	0.996856	-5.516682
H	-4.654755	1.512010	-3.943850
C	0.381452	-0.995471	1.781550
C	0.413972	-0.171622	4.153782
C	2.419607	-1.326901	3.161507
C	1.557688	-1.155333	4.393944
C	1.690811	-1.299907	1.894073
C	-0.447556	-0.612117	2.975500
H	0.828697	0.821008	3.956772
H	1.132366	-2.133620	4.657165
H	2.251660	-1.539638	1.002748
H	-1.072161	-1.466400	3.261163
H	-0.206011	-0.081609	5.049611
H	2.145300	-0.832389	5.253975
H	-1.137609	0.187535	2.689639
C	3.748835	-1.549425	3.211890
C	4.538616	-1.577089	4.501269

H	4.118748	-0.894337	5.243910
H	5.552013	-1.213171	4.295805
C	-3.146399	4.479943	1.526749
C	-4.673820	2.325444	2.594049
C	-2.600938	3.193853	1.701043
C	-4.481516	4.625496	1.917981
C	-5.234760	3.581022	2.438684
C	-3.351480	2.146903	2.225235
H	-4.964261	5.586366	1.815352
H	-6.267258	3.758640	2.717745
H	-2.899470	1.166955	2.318231
H	-5.241191	1.483545	2.967975
C	-2.378719	5.678264	0.933017
C	-1.089921	5.964017	1.726661
H	-1.281543	5.978483	2.801983
H	-0.693118	6.941373	1.437025
H	-0.308690	5.233339	1.526210
C	-2.038852	5.408833	-0.543921
H	-2.943177	5.184369	-1.116109
H	-1.343448	4.581054	-0.668386
H	-1.569874	6.296108	-0.980755
C	-3.222610	6.963498	0.974613
H	-3.495523	7.240857	1.996502
H	-4.134387	6.879654	0.377658
H	-2.634028	7.782952	0.555190
N	-1.250289	2.871198	1.342254
C	-0.870418	2.255935	0.168816
C	-0.159455	2.872851	2.242238
C	0.583829	1.957449	0.273590
H	1.156395	1.495755	-0.520013
C	0.998782	2.320774	1.481287
H	1.984176	2.233604	1.911525
O	-0.212732	3.234467	3.383638
O	-1.610906	2.007045	-0.766722
N	4.762706	1.526211	-0.853394
C	4.045481	2.155391	-1.938906
C	3.109173	1.121786	-2.590252
O	2.528272	0.345598	-1.757469
H	4.242940	0.868674	-0.291521
H	4.777641	2.485012	-2.676887
C	3.215720	3.353184	-1.473049
C	1.660140	5.515583	-0.611105
C	3.495043	3.991116	-0.268674
C	2.152159	3.818358	-2.248933
C	1.376861	4.886679	-1.818182
C	2.723582	5.066281	0.160230
H	4.312644	3.634437	0.346352
H	1.928685	3.334933	-3.193665
H	0.548250	5.229131	-2.428088
H	2.952717	5.549498	1.103666
H	1.054127	6.348641	-0.275593
O	2.931191	1.130362	-3.807962
C	6.114106	1.556767	-0.750365
O	6.507268	0.732492	0.239091
O	6.844450	2.231472	-1.445094

C	7.916158	0.610917	0.607324
C	7.886521	-0.398771	1.747892
H	7.250599	-0.041958	2.561443
H	8.897483	-0.549525	2.133222
H	7.498118	-1.358340	1.400995
C	8.722718	0.061777	-0.566035
H	9.745426	-0.140457	-0.237632
H	8.749586	0.771701	-1.391016
H	8.285320	-0.877433	-0.914654
C	8.445735	1.955876	1.096334
H	7.814234	2.337073	1.903112
H	8.470973	2.685391	0.288217
H	9.458265	1.825668	1.486568
N	-5.629812	-1.860251	0.479004
C	-5.584984	-0.645874	-0.292406
C	-4.189225	-0.014883	-0.258052
O	-3.187945	-0.627955	0.035112
H	-5.690909	-2.745873	0.006402
H	-6.277074	0.092480	0.119289
C	-5.934612	-0.912316	-1.746041
C	-6.513127	-1.354289	-4.439984
C	-5.320185	-1.955433	-2.438747
C	-6.833943	-0.088601	-2.415190
C	-7.121413	-0.308298	-3.756883
C	-5.612524	-2.178898	-3.777179
H	-4.603567	-2.597439	-1.936915
H	-7.310216	0.728532	-1.884882
H	-7.825439	0.338258	-4.267794
H	-5.131314	-2.995304	-4.303271
H	-6.739903	-1.525816	-5.485772
O	-4.216913	1.245204	-0.637286
H	-3.304106	1.618996	-0.629863
C	-5.284497	-1.869270	1.794604
O	-5.190631	-3.130795	2.226726
O	-5.108376	-0.863464	2.450367
C	-4.784292	-3.442357	3.598397
C	-4.786484	-4.965697	3.608653
H	-5.782567	-5.348957	3.376465
H	-4.493774	-5.330126	4.595727
H	-4.080885	-5.354282	2.870818
C	-3.380229	-2.905341	3.856401
H	-3.028763	-3.259603	4.828567
H	-3.369873	-1.816196	3.855274
H	-2.690937	-3.270589	3.090115
C	-5.806507	-2.893418	4.588195
H	-6.807702	-3.249273	4.332406
H	-5.805490	-1.804495	4.592102
H	-5.562509	-3.249564	5.592124
H	1.291236	-0.194223	-2.519222
C	4.577840	-1.841868	1.981696
H	4.031678	-2.389367	1.211563
H	4.972032	-0.924436	1.527960
H	5.446106	-2.450929	2.250519
C	4.627567	-2.984186	5.103855
H	5.050336	-3.691846	4.384475
H	5.256898	-2.996062	5.998364
H	3.633184	-3.345659	5.379739

21. EG3-anti

Imaginary Frequencies = 0 (Equilibrium Geometry)

Energy (Hartree) = -3797.76460561

C	4.646967	-4.961463	-0.262529
C	3.597924	-5.615182	0.302929
C	2.332199	-4.983502	0.448563
C	2.178858	-3.642466	0.003119
C	3.295634	-2.970042	-0.571516
C	4.494809	-3.623357	-0.714981
H	5.612915	-5.436094	-0.386360
H	3.690883	-6.635463	0.654837
C	0.895377	-3.046811	0.160410
H	3.198881	-1.945149	-0.905704
C	-0.091347	-3.796868	0.751506
C	0.168935	-5.116981	1.156458
H	-1.075572	-3.373937	0.920086
H	-0.621499	-5.704198	1.616903
N	1.332172	-5.705563	1.013747
O	5.599173	-3.091466	-1.279132
C	5.488721	-1.794189	-1.843163
H	5.200580	-1.065489	-1.082567
H	6.473290	-1.545418	-2.231629
H	4.753891	-1.775936	-2.653936
C	0.600977	-1.609711	-0.255981
H	1.542933	-1.074146	-0.352620
N	-0.162991	-0.901627	0.750475
H	-1.170637	-0.962895	0.688852
C	-0.129250	-1.421000	-1.615470
H	-0.629852	-0.457500	-1.510350
C	-1.150555	-2.470579	-2.075545
C	0.145091	-0.401481	-3.802934
C	1.341134	-2.476759	-3.338493
C	-1.128787	-2.539171	-3.610769
H	-0.909949	-3.458431	-1.676912
H	-2.132046	-2.194919	-1.694746
C	-1.161352	-1.122797	-4.230137
H	-0.052756	0.584002	-3.379037
H	0.853156	-0.284212	-4.622750
C	0.182496	-3.209560	-4.036926
H	1.768905	-3.064168	-2.529863
H	2.148949	-2.203521	-4.017527
H	-1.987484	-3.107188	-3.971128
H	-1.155288	-1.235879	-5.317788
H	0.184878	-4.262020	-3.747525
H	0.293661	-3.165142	-5.122928
N	0.833432	-1.203842	-2.750595
C	-2.391893	-0.355882	-3.840129
H	-2.454302	-0.045080	-2.801933
C	-3.383429	-0.048495	-4.664277
H	-3.359745	-0.330569	-5.713314

H	-4.257179	0.487286	-4.312754	O	2.722526	0.167422	-1.620443
C	0.372498	-0.634240	1.994400	H	4.083743	1.027631	0.112917
C	-0.053097	0.435830	4.236481	H	5.134671	2.179339	-2.377632
C	2.245990	-0.306718	3.579854	C	3.400139	3.208202	-1.607711
C	1.238294	-0.237847	4.703050	C	1.768598	5.464011	-1.310063
C	1.693245	-0.630703	2.264611	C	3.531317	4.036530	-0.496873
C	-0.653105	-0.298591	3.042493	C	2.448319	3.530191	-2.576633
H	0.162630	1.472350	3.962535	C	1.635566	4.646001	-2.425801
H	1.009252	-1.260193	5.032336	C	2.721704	5.157404	-0.347894
H	2.412245	-0.860954	1.484433	H	4.261868	3.790306	0.264439
H	-1.125933	-1.232597	3.369339	H	2.341259	2.898597	-3.451589
H	-0.781356	0.468188	5.051266	H	0.893697	4.875725	-3.182584
H	1.639701	0.292859	5.567322	H	2.833166	5.788304	0.527109
H	-1.449990	0.298237	2.586500	H	1.130909	6.331889	-1.191941
C	3.565934	-0.112547	3.726455	O	3.475494	0.700846	-3.652081
H	4.183885	-0.197460	2.833067	C	6.066713	1.195936	-0.261894
C	4.316251	0.198027	4.985978	O	6.179375	0.507885	0.888648
H	5.040212	-0.593558	5.211026	O	6.978079	1.503044	-0.999964
H	3.667812	0.306338	5.856497	C	7.476692	0.047580	1.381074
H	4.891110	1.125059	4.879976	C	7.115045	-0.642428	2.690056
C	-3.211647	4.602516	0.523708	H	6.666166	0.068455	3.386503
C	-4.829556	2.657535	1.830959	H	8.014146	-1.059654	3.149166
C	-2.692260	3.365516	0.951405	H	6.404337	-1.453265	2.513372
C	-4.569746	4.808556	0.787140	C	8.086667	-0.951974	0.403076
C	-5.367009	3.866000	1.424012	H	9.005781	-1.358592	0.832690
C	-3.486503	2.421002	1.591671	H	8.326440	-0.474854	-0.546405
H	-5.034970	5.736518	0.488227	H	7.397168	-1.781062	0.225527
H	-6.414807	4.085717	1.595551	C	8.394039	1.239008	1.640190
H	-3.050853	1.472875	1.883409	H	7.896046	1.961652	2.291920
H	-5.427924	1.891797	2.305842	H	8.671679	1.731568	0.709602
C	-2.389640	5.690966	-0.196097	H	9.300846	0.892956	2.142751
C	-1.185191	6.128775	0.658340	N	-5.452523	-1.943785	0.620857
H	-1.483879	6.328375	1.690005	C	-5.380631	-0.963887	-0.430745
H	-0.757514	7.045914	0.242940	C	-4.020112	-0.261478	-0.444581
H	-0.390841	5.384864	0.667738	O	-3.012155	-0.748614	0.013468
C	-1.906362	5.181702	-1.566034	H	-5.593605	-2.913295	0.394831
H	-2.747602	4.831638	-2.170372	H	-6.138914	-0.189099	-0.292169
H	-1.185469	4.370946	-1.482068	C	-5.563280	-1.615391	-1.790141
H	-1.413340	5.995950	-2.106171	C	-5.824984	-2.763838	-4.318692
C	-3.233173	6.950356	-0.457601	C	-4.836856	-2.758331	-2.124216
H	-3.597692	7.399249	0.470408	C	-6.415759	-1.049773	-2.732433
H	-4.087177	6.747785	-1.109215	C	-6.545397	-1.621484	-3.992315
H	-2.608452	7.692514	-0.960369	C	-4.971846	-3.332811	-3.380733
N	-1.321662	2.994937	0.754319	H	-4.159072	-3.200859	-1.401736
C	-0.850240	2.206848	-0.274845	H	-6.978099	-0.157401	-2.480741
C	-0.305276	3.151324	1.723041	H	-7.213522	-1.173907	-4.718918
C	0.595282	1.967257	-0.022774	H	-4.406590	-4.224216	-3.627638
H	1.229378	1.402937	-0.693782	H	-5.928754	-3.210045	-5.300890
C	0.911952	2.510160	1.147129	O	-4.081318	0.894744	-1.070933
H	1.858017	2.502108	1.665514	H	-3.188981	1.314809	-1.091678
O	-0.448811	3.683773	2.787835	C	-5.252163	-1.603995	1.920499
O	-1.517393	1.784331	-1.203244	O	-5.292454	-2.697465	2.687962
N	4.757671	1.501579	-0.470785	O	-5.072420	-0.463189	2.294975
C	4.267399	1.958146	-1.754268	C	-5.063602	-2.625055	4.132902
C	3.441682	0.845052	-2.432487	C	-5.202866	-4.079308	4.564946

H	-6.201356	-4.454672	4.330111
H	-5.043351	-4.162691	5.642162
H	-4.465500	-4.702721	4.054364
C	-3.652462	-2.115261	4.407003
H	-3.442483	-2.185294	5.477108
H	-3.539136	-1.077759	4.094874
H	-2.922447	-2.730558	3.874373
C	-6.131977	-1.761689	4.796189
H	-7.128169	-2.113275	4.515888
H	-6.029215	-0.716318	4.509662
H	-6.034143	-1.841053	5.881727
H	1.668339	-0.635105	-2.373074

22. EG9-anti
Imaginary Frequencies = 0 (Equilibrium Geometry)
Energy (Hartree) = -3876.39736224

C	3.415527	6.283956	-0.332158
C	2.194363	6.836288	-0.549700
C	1.011227	6.055060	-0.455541
C	1.118234	4.673784	-0.127580
C	2.408311	4.121254	0.128618
C	3.527576	4.911741	0.015571
H	4.326375	6.865205	-0.408728
H	2.082560	7.882348	-0.807626
C	-0.094301	3.922229	-0.112953
H	2.512574	3.079795	0.407107
C	-1.262867	4.581338	-0.407983
C	-1.255780	5.959809	-0.675972
H	-2.192775	4.029924	-0.472931
H	-2.190098	6.473494	-0.887293
N	-0.164316	6.684469	-0.706008
O	4.798769	4.500506	0.201072
C	5.019742	3.189653	0.691987
H	4.618800	2.433333	0.011382
H	6.099349	3.074446	0.763522
H	4.568854	3.066511	1.680551
C	-0.150566	2.415849	0.119497
H	0.855740	2.004478	0.011882
N	-0.996868	1.789224	-0.874704
H	-1.803448	1.277458	-0.536890
C	-0.623013	1.921713	1.505649
H	-0.778399	0.853588	1.348903
C	-1.877575	2.536271	2.138565
C	0.212968	0.916930	3.550117
C	0.598097	3.301880	3.189794
C	-1.714276	2.492580	3.666604
H	-2.008563	3.575463	1.829480
H	-2.753152	1.979109	1.804986
C	-1.214103	1.102593	4.126059
H	0.336053	-0.045811	3.052236
H	0.994992	1.012766	4.301906
C	-0.653219	3.531967	4.057863
H	0.700217	4.051195	2.407129
H	1.526913	3.272622	3.758057
H	-2.665394	2.713528	4.152958
H	-1.143957	1.116866	5.217090
H	-1.033450	4.541928	3.894819
H	-0.409150	3.438814	5.118807
N	0.476259	1.972852	2.525376
C	-2.136708	-0.015028	3.728358
H	-2.248470	-0.197460	2.662726
C	-2.806608	-0.779809	4.578927
H	-2.724675	-0.642467	5.653546
H	-3.468584	-1.561690	4.225651
C	-0.453434	1.312985	-2.048180
C	-0.725390	-0.288869	-3.967114
C	1.402568	0.977314	-3.654744
C	0.391385	0.487912	-4.666153
C	0.824247	1.527464	-2.431745
C	-1.428446	0.562070	-2.914854
H	-0.282939	-1.172083	-3.495977
H	-0.046390	1.348028	-5.190079
H	1.471623	2.122928	-1.803362
H	-2.078507	1.297080	-3.403927
H	-1.454073	-0.653073	-4.696894
H	0.853702	-0.149941	-5.419544
H	-2.088791	-0.046581	-2.287376
C	2.736891	0.897951	-3.829944
C	-1.336263	-4.709447	-0.379292
C	-3.376326	-3.801568	-2.147641
C	-1.175643	-3.563275	-1.178700
C	-2.561063	-5.371568	-0.518938
C	-3.562547	-4.935419	-1.375584
C	-2.173323	-3.126613	-2.043946
H	-2.753533	-6.261503	0.062409
H	-4.493001	-5.489010	-1.430794
H	-1.998535	-2.235303	-2.631286
H	-4.154456	-3.425664	-2.797920
C	-0.303715	-5.218372	0.647481
C	1.132460	-5.227870	0.085652
H	1.160851	-5.617232	-0.934310
H	1.763229	-5.869545	0.705907
H	1.589713	-4.238067	0.091136
C	-0.375954	-4.333719	1.906834
H	-1.396917	-4.304448	2.297623
H	-0.061002	-3.308348	1.712461
H	0.276006	-4.736959	2.686810
C	-0.617080	-6.662341	1.080439
H	-0.645194	-7.344543	0.226177
H	-1.562586	-6.739591	1.621763
H	0.167317	-7.004781	1.759452
N	0.000470	-2.745634	-1.126463
C	0.146962	-1.644396	-0.319673
C	1.119649	-2.844687	-1.991810
C	1.473417	-1.046668	-0.613105
H	1.844023	-0.203855	-0.044155
C	2.049005	-1.751216	-1.582306
H	3.018745	-1.601545	-2.032335

O	1.245535	-3.660004	-2.860512
O	-0.665143	-1.246692	0.498671
N	4.697667	-0.421377	0.609796
C	4.542019	-0.227505	2.031532
C	3.455033	0.841831	2.284555
O	2.638567	1.041812	1.325742
H	3.983823	-0.029619	0.017478
H	5.475526	0.140784	2.462944
C	4.122798	-1.483058	2.788837
C	3.307078	-3.722713	4.250859
C	3.511120	-2.556741	2.149719
C	4.317272	-1.537914	4.168070
C	3.909576	-2.647535	4.895763
C	3.113365	-3.673297	2.876551
H	3.356938	-2.530211	1.076658
H	4.782507	-0.697458	4.670301
H	4.070313	-2.676414	5.967723
H	2.655491	-4.509303	2.362727
H	2.997667	-4.595484	4.814736
O	3.419861	1.393801	3.383955
C	5.603968	-1.279464	0.082773
O	5.395286	-1.403132	-1.245718
O	6.463877	-1.844278	0.721741
C	6.108218	-2.414539	-2.023728
C	5.554221	-2.225206	-3.430610
H	4.480664	-2.426449	-3.463805
H	6.047397	-2.917352	-4.116692
H	5.732039	-1.205700	-3.779418
C	7.609908	-2.143512	-2.009211
H	8.105475	-2.825047	-2.705395
H	8.025629	-2.287166	-1.013954
H	7.807910	-1.118640	-2.333901
C	5.757683	-3.802779	-1.494711
H	4.672419	-3.939676	-1.488561
H	6.140978	-3.947038	-0.485220
H	6.193272	-4.562262	-2.148665
N	-6.122714	-0.025311	-0.316277
C	-5.413396	-0.815859	0.658831
C	-3.900687	-0.705603	0.455916
O	-3.367079	0.302847	0.047765
H	-6.604117	0.809029	-0.026351
H	-5.680381	-1.869914	0.561344
C	-5.718140	-0.338163	2.066571
C	-6.216897	0.542285	4.664948
C	-5.494319	0.994592	2.414612
C	-6.188330	-1.225471	3.028497
C	-6.436165	-0.786464	4.324323
C	-5.746790	1.433149	3.706464
H	-5.111371	1.687169	1.672234
H	-6.360628	-2.263030	2.763958
H	-6.803119	-1.485057	5.067317
H	-5.570086	2.470487	3.966643
H	-6.410934	0.883282	5.675274
O	-3.268216	-1.789424	0.844908
H	-2.296981	-1.675190	0.703869
C	-5.884197	-0.207840	-1.642887
O	-6.502903	0.736216	-2.357299
O	-5.207893	-1.118590	-2.075921
C	-6.369535	0.794949	-3.814579
C	-7.186729	2.029338	-4.174213
H	-8.224054	1.907989	-3.854482
H	-7.170661	2.182450	-5.255488
H	-6.772835	2.916750	-3.690295
C	-4.905321	0.989599	-4.197259
H	-4.835347	1.187947	-5.269624
H	-4.314235	0.105321	-3.962985
H	-4.488461	1.846553	-3.661790
C	-6.973590	-0.454544	-4.447414
H	-8.002333	-0.591180	-4.104662
H	-6.394031	-1.342028	-4.197698
H	-6.987299	-0.337134	-5.533872
H	1.390895	1.712731	2.049457
C	3.717549	1.351704	-2.768694
H	3.300920	1.204008	-1.766809
H	4.610191	0.719140	-2.815470
C	3.384748	0.400438	-5.098575
H	3.665535	-0.655826	-5.016027
H	2.750050	0.509255	-5.978485
H	4.308061	0.955920	-5.292472
C	4.121507	2.821586	-2.934560
H	4.882069	3.117321	-2.207419
H	4.526252	3.003166	-3.934466
H	3.256124	3.476457	-2.799647

23. Endo-iPr-HMe-Dienamine
Imaginary Frequencies = 0 (Equilibrium Geometry)
Energy (Hartree) = -485.327468605

C	-2.463175	1.099901	-0.118771
C	-1.905229	-0.090086	-0.380994
C	-0.448292	-0.229241	-0.455622
C	0.365743	0.738618	0.024634
C	-0.251417	1.949732	0.685776
C	-1.592662	2.316742	0.053040
C	-2.725555	-1.336100	-0.597754
C	-2.536238	-2.371579	0.515234
N	1.737858	0.717331	0.000338
C	2.515150	-0.475270	-0.301709
C	2.431258	-1.524660	0.810246
C	3.956588	-0.064918	-0.580755
H	-3.542469	1.210867	-0.081557
H	-0.045571	-1.148885	-0.865151
H	0.436939	2.798410	0.634869
H	-0.394929	1.712371	1.749319
H	-1.412040	2.777388	-0.930188
H	-2.090961	3.074205	0.663391
H	-2.443157	-1.786744	-1.557484
H	-3.783077	-1.067046	-0.674019
H	-3.131454	-3.268416	0.323957

H	-1.488592	-2.673383	0.599730
H	-2.842523	-1.956559	1.479484
H	2.189035	1.374784	0.618556
H	2.099395	-0.900850	-1.221197
H	2.954216	-2.440045	0.520249
H	2.893751	-1.140845	1.725155
H	1.391751	-1.776924	1.030461
H	4.557839	-0.935932	-0.850029
H	4.003174	0.658765	-1.397257
H	4.406647	0.388693	0.309398

24. H2O
Imaginary Frequencies = 0 (Equilibrium Geometry)
Energy (Hartree) = -76.427561292

O	0.000000	0.000000	0.118108
H	-0.000000	0.754002	-0.472432
H	-0.000000	-0.754002	-0.472432

25. Iso-iPr-HMe-Dienamine
Imaginary Frequencies = 0 (Equilibrium Geometry)
Energy (Hartree) = -485.323240972

C	-2.004110	1.573687	0.304223
C	-2.047762	0.081481	0.071836
C	-0.905709	-0.560800	-0.190829
C	0.386292	0.146343	-0.266696
C	0.404582	1.487986	-0.382919
C	-0.896181	2.240678	-0.507947
C	-3.387793	-0.584501	0.215752
C	-3.406450	-2.103171	0.098409
N	1.497122	-0.692118	-0.279692
C	2.850690	-0.178202	-0.133794
C	3.835063	-1.279030	-0.515132
C	3.136438	0.365204	1.270039
H	-1.832858	1.743740	1.377753
H	-2.975311	2.020004	0.069019
H	-0.889077	-1.636294	-0.340708
H	1.333799	2.038369	-0.465763
H	-1.206192	2.289924	-1.563490
H	-0.770597	3.274958	-0.176757
H	-4.064162	-0.148823	-0.530910
H	-3.805252	-0.282692	1.186031
H	-4.422189	-2.483417	0.227022
H	-3.048981	-2.430453	-0.881735
H	-2.774324	-2.568116	0.859987
H	1.348333	-1.558925	0.218670
H	2.958124	0.637703	-0.856475
H	4.863317	-0.914417	-0.457592
H	3.743967	-2.129655	0.169732
H	3.643859	-1.634018	-1.529893
H	4.132010	0.815068	1.318957
H	2.402007	1.121586	1.553859
H	3.093324	-0.447765	2.002489

26. N-Boc-L-Phenylglycine
Imaginary Frequencies = 0 (Equilibrium Geometry)
Energy (Hartree) = -861.286878851

N	-0.231009	0.983676	0.190897
C	1.073161	0.998499	-0.413675
C	1.816554	2.210771	0.126267
O	1.475384	2.842877	1.091328
H	-0.391733	1.552305	1.007084
H	0.977816	1.114159	-1.496494
C	1.895754	-0.255673	-0.133792
C	3.432014	-2.519655	0.395799
C	1.891706	-0.823228	1.137410
C	2.671751	-0.825156	-1.137145
C	3.437592	-1.954525	-0.874058
C	2.656605	-1.952066	1.401132
H	1.278066	-0.387412	1.918453
H	2.670008	-0.389320	-2.130415
H	4.032874	-2.397640	-1.664197
H	2.645840	-2.390354	2.392535
H	4.025807	-3.403117	0.600554
O	2.911081	2.473264	-0.585057
H	3.365007	3.216113	-0.168158
C	-1.237578	0.241169	-0.337836
O	-2.338510	0.372793	0.414960
O	-1.124146	-0.426593	-1.343558
C	-3.577352	-0.325691	0.076377
C	-4.522334	0.112020	1.188827
H	-4.131157	-0.188888	2.163274
H	-5.501703	-0.350739	1.047869
H	-4.644868	1.197409	1.181734
C	-4.092356	0.143407	-1.281257
H	-5.082692	-0.283593	-1.458635
H	-3.425458	-0.168877	-2.083511
H	-4.181719	1.232728	-1.294148
C	-3.357507	-1.835052	0.120861
H	-2.935660	-2.125688	1.086430
H	-2.684598	-2.157037	-0.672427
H	-4.317308	-2.343417	-0.001070

27. OPEN-TS1-anti
Imaginary Frequencies = 1 (Transition State)
Energy (Hartree) = -3797.74420813

C	3.866214	6.731108	-1.762147
C	2.615536	7.248601	-1.869267
C	1.468005	6.414733	-1.777728
C	1.643725	5.017562	-1.579261
C	2.963581	4.499009	-1.447858
C	4.048654	5.338638	-1.542442
H	4.749198	7.354710	-1.830817

H	2.451428	8.307522	-2.026723
C	0.461386	4.219766	-1.515918
H	3.127454	3.448610	-1.243955
C	-0.749121	4.855642	-1.653946
C	-0.805286	6.249865	-1.829353
H	-1.685813	4.311921	-1.647141
H	-1.770320	6.739564	-1.925583
N	0.256024	7.013267	-1.892104
O	5.331716	4.956381	-1.442837
C	5.602575	3.590711	-1.149885
H	5.218720	2.937174	-1.939765
H	6.686483	3.508658	-1.106914
H	5.167523	3.306903	-0.188273
C	0.536421	2.708795	-1.376416
H	1.561385	2.404824	-1.579645
N	-0.282936	2.088589	-2.408130
H	-1.237830	2.404524	-2.486582
C	0.159599	2.070185	-0.010743
H	0.156779	0.998320	-0.209962
C	-1.184438	2.439338	0.644907
C	0.968203	1.307516	2.148825
C	1.377324	3.631538	1.560363
C	-0.948059	2.901995	2.087120
H	-1.694681	3.236312	0.107171
H	-1.847734	1.579096	0.619502
C	-0.283778	1.784328	2.920689
H	0.817561	0.317427	1.721828
H	1.856972	1.287317	2.777410
C	0.002091	4.105345	2.057544
H	1.795898	4.269330	0.788759
H	2.121129	3.556854	2.352129
H	-1.903309	3.180323	2.533665
H	0.036292	2.220827	3.871975
H	-0.397702	4.874825	1.392593
H	0.101252	4.545353	3.051520
N	1.245491	2.245620	1.012583
C	-1.184671	0.621308	3.216661
H	-1.654640	0.146160	2.359107
C	-1.390820	0.130982	4.431475
H	-0.919771	0.566758	5.307993
H	-2.034093	-0.727690	4.589185
C	0.001215	0.953326	-3.052932
C	-1.092586	-1.031774	-4.021182
C	1.381144	-1.013309	-3.497428
C	0.276291	-1.637811	-4.313337
C	1.172648	0.232268	-2.881605
C	-1.020587	0.488069	-4.049515
H	-1.432361	-1.320961	-3.024189
H	0.519671	-1.487832	-5.373720
H	1.937987	0.605175	-2.215710
H	-0.708361	0.842494	-5.039369
H	-1.832658	-1.390340	-4.740284
H	0.276910	-2.720783	-4.155675
H	-1.993483	0.922306	-3.822272
C	2.537294	-1.745319	-3.228573
C	-1.834577	-4.212919	0.386315
C	-3.395222	-4.313150	-1.990558
C	-1.380847	-3.646478	-0.819718
C	-3.139179	-4.727847	0.357748
C	-3.911558	-4.778190	-0.790395
C	-2.138085	-3.741086	-1.987235
H	-3.567081	-5.125307	1.267760
H	-4.909212	-5.200846	-0.745466
H	-1.715873	-3.353865	-2.903649
H	-3.969095	-4.369052	-2.907405
C	-1.046947	-4.334481	1.712062
C	0.482329	-4.341650	1.529764
H	0.786828	-5.038794	0.744067
H	0.949462	-4.672005	2.461398
H	0.894286	-3.359110	1.306438
C	-1.449132	-3.190057	2.658611
H	-2.531460	-3.180277	2.819174
H	-1.153356	-2.221167	2.256047
H	-0.965376	-3.322360	3.632031
C	-1.391753	-5.675960	2.396391
H	-1.216660	-6.520213	1.723851
H	-2.423523	-5.723566	2.748109
H	-0.753087	-5.802004	3.274222
N	-0.145431	-2.941498	-0.936838
C	0.139956	-1.706485	-0.307058
C	1.013511	-3.448045	-1.514502
C	1.525224	-1.448816	-0.448702
H	2.032383	-0.599550	-0.021758
C	2.079198	-2.420556	-1.268089
H	3.106216	-2.751485	-1.216385
O	1.110257	-4.513106	-2.077898
O	-0.727869	-1.056000	0.293346
C	3.861033	-1.080253	-2.933669
H	4.274136	-0.639141	-3.846647
H	3.771447	-0.276659	-2.197494
H	4.585388	-1.804052	-2.554316
H	2.611432	-2.711409	-3.723813
N	-5.731170	0.480101	0.310045
C	-5.057784	0.466027	-0.965301
C	-3.572765	0.108594	-0.856892
O	-3.301378	-0.777606	0.072799
H	-6.529797	-0.110312	0.467854
H	-5.074189	1.464100	-1.409530
C	-5.727734	-0.509554	-1.912972
C	-6.951185	-2.297907	-3.669497
C	-6.177405	-0.076305	-3.157305
C	-5.884039	-1.848749	-1.556134
C	-6.500382	-2.735250	-2.429117
C	-6.784060	-0.967412	-4.034575
H	-6.055002	0.963728	-3.439606
H	-5.503298	-2.202238	-0.604015
H	-6.619751	-3.773947	-2.143091
H	-7.131459	-0.619907	-5.000660
H	-7.429214	-2.992820	-4.350255
O	-2.759954	0.594384	-1.612375

C	-5.334716	1.333938	1.286680
O	-6.132809	1.217122	2.352440
O	-4.386778	2.086099	1.166712
C	-5.935548	2.035193	3.550444
C	-7.060855	1.565296	4.463630
H	-8.032712	1.744137	3.998163
H	-7.021775	2.109353	5.409938
H	-6.963563	0.497405	4.671286
C	-4.576443	1.732823	4.169127
H	-4.492274	2.243836	5.131735
H	-3.764216	2.062802	3.524560
H	-4.465965	0.659403	4.339646
C	-6.100424	3.513050	3.209316
H	-7.058350	3.681492	2.710585
H	-5.295949	3.859199	2.561969
H	-6.087336	4.098730	4.131914
H	-2.321289	-0.974260	0.088493
H	2.153666	1.941472	0.572259
N	4.851106	-1.082660	0.578436
H	4.343903	-0.860012	-0.263607
C	4.792901	-0.102398	1.632483
C	4.180492	1.210264	1.072944
O	4.347412	2.241093	1.727686
O	3.468687	1.102696	0.021890
C	3.918199	-0.507296	2.816210
C	2.954416	-1.505211	2.710486
C	2.080197	-1.756003	3.761625
C	2.165701	-1.019751	4.936177
C	3.146203	-0.040305	5.059536
C	4.017933	0.210953	4.007706
C	5.377024	-2.317676	0.742355
O	5.933713	-2.704020	1.746579
O	5.187429	-3.043662	-0.383684
C	5.359284	-4.494725	-0.387683
C	4.938127	-4.890175	-1.799696
C	6.821222	-4.857796	-0.146405
C	4.422819	-5.119618	0.643312
H	4.762066	0.994325	4.091910
H	3.231719	0.531883	5.976781
H	1.476766	-1.210781	5.751011
H	1.322818	-2.523600	3.654583
H	2.863288	-2.073469	1.792816
H	5.797256	0.123992	1.997298
H	5.534641	-4.351385	-2.539949
H	5.097122	-5.961564	-1.941972
H	3.879701	-4.681700	-1.974686
H	4.709826	-4.838998	1.656465
H	3.393416	-4.797722	0.460969
H	4.458866	-6.208246	0.555827
H	7.133083	-4.577658	0.857891
H	6.951933	-5.935515	-0.274324
H	7.458210	-4.346246	-0.872658

28. PRC1

Imaginary Frequencies = 0 (Equilibrium Geometry)

Energy (Hartree) = -3797.78317238

C	-6.400359	-4.915759	-0.203084
C	-5.501831	-5.932663	-0.245449
C	-4.104452	-5.673158	-0.274542
C	-3.653925	-4.323781	-0.258918
C	-4.612394	-3.271731	-0.194704
C	-5.955897	-3.565473	-0.176340
H	-7.467776	-5.098879	-0.184491
H	-5.817877	-6.968462	-0.260968
C	-2.244659	-4.121833	-0.312927
H	-4.298566	-2.236468	-0.142055
C	-1.426229	-5.224191	-0.377123
C	-1.984763	-6.514226	-0.371067
H	-0.347945	-5.124117	-0.437806
H	-1.332490	-7.381854	-0.412159
N	-3.273824	-6.743593	-0.323754
O	-6.945661	-2.659018	-0.135504
C	-6.593597	-1.283082	-0.066952
H	-6.010608	-0.983632	-0.943819
H	-7.535314	-0.738532	-0.053702
H	-6.026272	-1.067488	0.841993
C	-1.643299	-2.729309	-0.360756
H	-2.428285	-2.022907	-0.614028
N	-0.656596	-2.687288	-1.440544
H	0.244414	-3.197019	-1.270255
C	-0.969079	-2.189425	0.934478
H	-0.308736	-1.394091	0.586848
C	-0.171338	-3.163259	1.815770
C	-1.225179	-0.506018	2.668289
C	-2.725438	-2.448557	2.694287
C	-0.321907	-2.740725	3.286949
H	-0.547173	-4.182584	1.715473
H	0.867385	-3.174910	1.491061
C	-0.098162	-1.223890	3.455170
H	-0.833034	0.261311	2.003029
H	-1.962285	-0.051604	3.329172
C	-1.758236	-3.058862	3.721984
H	-3.169252	-3.202726	2.048055
H	-3.533880	-1.866576	3.132885
H	0.392975	-3.288408	3.902004
H	-0.225952	-0.977748	4.512930
H	-1.909971	-4.138481	3.772775
H	-1.948504	-2.651500	4.717480
N	-1.964984	-1.499090	1.819934
C	1.274400	-0.775369	3.036621
H	1.613451	-1.076839	2.048461
C	2.076433	-0.025990	3.781367
H	1.782848	0.312266	4.771502
H	3.049016	0.279223	3.411512
C	-0.652534	-1.877559	-2.458926
C	0.116727	-1.431969	-4.787138
C	-1.356912	0.170893	-3.528292
C	-0.394989	-0.002801	-4.660450

C	-1.583934	-0.802218	-2.616728
C	0.490326	-1.991407	-3.420511
H	0.984053	-1.467797	-5.448019
H	-0.898373	0.317461	-5.578808
H	-2.322593	-0.633738	-1.846592
H	0.817158	-3.029755	-3.482437
H	-0.659430	-2.060069	-5.235157
H	0.427439	0.712282	-4.530273
H	1.314051	-1.422985	-2.971749
C	-1.975548	1.530999	-3.332338
C	3.302152	2.635011	-0.770620
C	4.399244	0.757896	-2.607916
C	2.493974	1.920405	-1.666669
C	4.666561	2.300123	-0.786628
C	5.211415	1.388998	-1.674280
C	3.044645	1.018667	-2.579742
H	5.331509	2.772183	-0.074892
H	6.270972	1.164854	-1.633795
H	2.375028	0.505558	-3.258650
H	4.808949	0.041578	-3.309799
C	2.855973	3.727727	0.224299
C	1.443622	4.291857	-0.007483
H	1.306653	4.625370	-1.039792
H	1.308026	5.161646	0.641537
H	0.653573	3.585971	0.243900
C	2.938401	3.157542	1.651003
H	3.946242	2.803494	1.876970
H	2.249282	2.319489	1.771746
H	2.682736	3.933951	2.379960
C	3.815917	4.931205	0.101068
H	3.814962	5.326495	-0.918587
H	4.844190	4.684689	0.369698
H	3.487380	5.726698	0.775379
N	1.069351	1.991687	-1.657503
C	0.258326	1.411174	-0.645512
C	0.270872	2.536447	-2.631503
C	-1.036570	1.560058	-0.906822
H	-1.838850	1.185514	-0.291109
C	-1.177722	2.276431	-2.212495
H	-1.670455	3.244707	-2.082681
O	0.648955	3.102366	-3.636437
O	0.847631	0.881075	0.423289
N	4.220686	-1.230294	0.549114
C	3.898961	-2.385218	-0.258945
C	2.391155	-2.402843	-0.553963
O	1.780750	-1.307775	-0.519246
H	3.881476	-0.328402	0.250222
H	4.135484	-3.269742	0.335688
C	4.724780	-2.432094	-1.550025
C	6.279815	-2.418860	-3.883741
C	6.061089	-2.033542	-1.515049
C	4.188981	-2.854521	-2.764299
C	4.957286	-2.840045	-3.924111
C	6.832231	-2.025967	-2.668903
H	6.503473	-1.717134	-0.578153
H	3.165881	-3.205298	-2.805399
H	4.516844	-3.161673	-4.861420
H	7.867917	-1.708784	-2.619602
H	6.878501	-2.404635	-4.787301
O	1.869736	-3.505012	-0.842663
C	5.077443	-1.290007	1.595415
O	5.203206	-0.070660	2.152222
O	5.640298	-2.299802	1.967124
C	6.199098	0.179769	3.192213
C	6.019862	1.664476	3.483265
H	6.201321	2.257068	2.583295
H	6.726337	1.978884	4.254623
H	5.006550	1.868555	3.837092
C	5.898985	-0.647247	4.439621
H	6.576920	-0.344274	5.241842
H	6.030793	-1.709940	4.245906
H	4.873659	-0.472674	4.774576
C	7.596623	-0.092113	2.642530
H	7.752117	0.470456	1.717920
H	7.740441	-1.153375	2.443261
H	8.343357	0.232624	3.371291
H	1.393534	0.105763	0.138148
H	-2.644776	-0.946581	1.233784
N	-3.932690	2.854189	0.318954
H	-3.700814	2.230987	-0.436477
C	-4.107620	2.257178	1.619249
C	-4.160915	0.712885	1.477507
O	-4.686815	0.080410	2.394923
O	-3.571371	0.209805	0.464453
C	-2.975314	2.545351	2.602335
C	-1.717498	2.958046	2.176215
C	-0.669846	3.081654	3.081537
C	-0.869997	2.801352	4.427097
C	-2.133605	2.415720	4.865084
C	-3.178905	2.292576	3.958963
C	-3.864528	4.191049	0.120159
O	-4.050406	5.025595	0.977946
O	-3.561660	4.441114	-1.174324
C	-3.216265	5.788174	-1.622574
C	-2.911412	5.596161	-3.105162
C	-4.403236	6.731259	-1.448024
C	-1.973461	6.267719	-0.877410
H	-4.632773	6.882288	-0.395178
H	-4.167428	7.696147	-1.904210
H	-5.283836	6.321190	-1.949338
H	-3.766310	5.146467	-3.615747
H	-2.704528	6.565036	-3.565008
H	-2.036015	4.959896	-3.256306
H	-2.182558	6.411864	0.182112
H	-1.164343	5.539260	-0.983974
H	-1.635878	7.216211	-1.302356
H	-5.050417	2.584282	2.062503
H	-4.156017	1.965073	4.295371
H	-2.305878	2.209146	5.915639
H	-0.050844	2.892456	5.131421

H	0.307022	3.389467	2.728147
H	-1.541983	3.161605	1.126931
C	-3.469260	1.492011	-3.003738
H	-3.842125	2.510035	-2.873345
H	-4.029000	1.015174	-3.811211
H	-3.672678	0.937159	-2.083220
H	-1.829896	2.115215	-4.245647

29. PRC10

Imaginary Frequencies = 0 (Equilibrium Geometry)

Energy (Hartree) = -3876.40708753

C	6.176663	-5.126952	0.097878
C	5.255278	-6.116870	0.217595
C	3.866222	-5.819971	0.272890
C	3.448571	-4.461894	0.199456
C	4.429645	-3.439661	0.050518
C	5.764675	-3.768801	0.012475
H	7.238364	-5.338166	0.059909
H	5.546272	-7.158328	0.278258
C	2.047344	-4.220951	0.289027
H	4.139378	-2.401121	-0.051208
C	1.204844	-5.296850	0.436452
C	1.730877	-6.599729	0.478504
H	0.132343	-5.165084	0.528941
H	1.059287	-7.446884	0.584693
N	3.011760	-6.864583	0.403130
O	6.775198	-2.891920	-0.100952
C	6.455458	-1.512777	-0.232879
H	5.898251	-1.154191	0.638615
H	7.409541	-0.993571	-0.293347
H	5.874183	-1.330909	-1.140159
C	1.480806	-2.813248	0.287493
H	2.287812	-2.112216	0.477465
N	0.526195	-2.693067	1.390098
H	-0.404815	-3.158245	1.252988
C	0.776534	-2.319331	-1.008846
H	0.158899	-1.483370	-0.678964
C	-0.087500	-3.307617	-1.807050
C	1.019466	-0.730823	-2.828870
C	2.444154	-2.729837	-2.824000
C	0.007796	-2.953967	-3.300969
H	0.264861	-4.332194	-1.677234
H	-1.110009	-3.274135	-1.436036
C	-0.171906	-1.437983	-3.524794
H	0.692678	0.083528	-2.184641
H	1.741425	-0.340607	-3.545299
C	1.411436	-3.340965	-3.785548
H	2.880562	-3.474007	-2.160960
H	3.258300	-2.203552	-3.319041
H	-0.753006	-3.501256	-3.858536
H	-0.091664	-1.241773	-4.597551
H	1.522614	-4.426556	-3.801689
H	1.571710	-2.977473	-4.803151
N	1.758722	-1.711415	-1.966132
C	-1.503180	-0.919473	-3.055811
H	-1.796225	-1.157468	-2.036076
C	-2.320452	-0.179612	-3.793709
H	-2.072163	0.097090	-4.814899
H	-3.260281	0.178866	-3.388289
C	0.595345	-1.877238	2.401591
C	-0.132386	-1.306901	4.708752
C	1.462096	0.151005	3.410818
C	0.457032	0.085084	4.524539
C	1.611603	-0.878164	2.543226
C	-0.543847	-1.900808	3.371371
H	-0.996043	-1.263205	5.374280
H	0.932581	0.413673	5.450879
H	2.373634	-0.812604	1.782927
H	-0.917344	-2.920070	3.474118
H	0.609908	-1.957096	5.182211
H	-0.328474	0.826805	4.337795
H	-1.344679	-1.314247	2.905064
C	2.193089	1.458411	3.135989
C	-3.188546	2.705803	0.705562
C	-4.298934	0.957462	2.657997
C	-2.379019	1.995130	1.603365
C	-4.565122	2.434228	0.784589
C	-5.117020	1.586622	1.728325
C	-2.936823	1.157148	2.571626
H	-5.234185	2.906926	0.077103
H	-6.186206	1.409809	1.734446
H	-2.267921	0.641834	3.248859
H	-4.712591	0.289497	3.403868
C	-2.738940	3.734502	-0.353159
C	-1.288217	4.226733	-0.220557
H	-1.078919	4.599897	0.785731
H	-1.139363	5.055732	-0.918368
H	-0.553185	3.465217	-0.475252
C	-2.930967	3.115901	-1.748953
H	-3.966397	2.807666	-1.907020
H	-2.293579	2.237960	-1.871985
H	-2.676387	3.848717	-2.522008
C	-3.624859	4.993123	-0.226285
H	-3.543101	5.425860	0.774707
H	-4.678770	4.793472	-0.425588
H	-3.294424	5.743175	-0.949993
N	-0.952671	2.002961	1.550031
C	-0.194921	1.359663	0.539109
C	-0.107111	2.566197	2.475467
C	1.110908	1.464697	0.763265
H	1.870407	1.037633	0.127784
C	1.325246	2.214061	2.043439
H	1.854652	3.155432	1.861294
O	-0.458758	3.226707	3.431469
O	-0.829400	0.829768	-0.504051
N	-4.387017	-1.135156	-0.448892
C	-4.070575	-2.274206	0.382533
C	-2.551134	-2.335878	0.604343

O	-1.906911	-1.265493	0.497622
H	-3.969419	-0.246581	-0.217079
H	-4.367615	-3.169484	-0.167367
C	-4.831534	-2.245618	1.713807
C	-6.260715	-2.095400	4.122376
C	-6.143218	-1.771850	1.737115
C	-4.258870	-2.674231	2.909103
C	-4.963829	-2.591455	4.105594
C	-6.852081	-1.696636	2.928044
H	-6.614845	-1.448468	0.817134
H	-3.256976	-3.083410	2.906045
H	-4.494378	-2.919173	5.026598
H	-7.869378	-1.321398	2.923575
H	-6.810041	-2.027998	5.054452
O	-2.052168	-3.443896	0.909472
C	-5.294040	-1.190679	-1.451110
O	-5.379062	0.009958	-2.055324
O	-5.929080	-2.181358	-1.751280
C	-6.401439	0.271157	-3.066096
C	-6.152026	1.728977	-3.432021
H	-6.267683	2.370679	-2.555175
H	-6.868121	2.047954	-4.192560
H	-5.142613	1.859442	-3.829244
C	-6.198916	-0.625872	-4.284456
H	-6.892447	-0.321603	-5.072711
H	-6.379655	-1.669835	-4.035753
H	-5.180542	-0.523750	-4.667250
C	-7.788083	0.103931	-2.451060
H	-7.874530	0.715590	-1.548879
H	-7.981222	-0.937422	-2.196198
H	-8.545215	0.437043	-3.165208
H	-1.427458	0.108066	-0.188336
H	2.482927	-1.155845	-1.437473
C	2.335384	2.347918	4.388741
H	1.354500	2.534662	4.825336
H	2.691612	3.325650	4.052724
N	3.946271	2.646690	-0.782261
H	3.666568	2.091546	0.008770
C	4.036079	1.970166	-2.052953
C	4.044230	0.435504	-1.836267
O	4.516481	-0.259146	-2.737559
O	3.471981	0.003226	-0.781422
C	2.863129	2.247023	-2.990185
C	1.635680	2.694068	-2.512698
C	0.547082	2.816081	-3.368186
C	0.673999	2.494124	-4.713419
C	1.905457	2.067818	-5.202234
C	2.992961	1.949346	-4.346332
C	3.905598	3.997008	-0.683826
O	4.123323	4.756648	-1.601694
O	3.592312	4.356447	0.581257
C	3.324325	5.755486	0.912104
C	2.978999	5.707515	2.396287
C	4.573061	6.604741	0.691271
C	2.127117	6.251177	0.105476
H	4.834004	6.651241	-0.364056
H	4.389301	7.617794	1.058172
H	5.414792	6.185781	1.249001
H	3.786454	5.235001	2.961132
H	2.841571	6.723039	2.773989
H	2.052411	5.156076	2.573101
H	2.364064	6.311883	-0.955848
H	1.278345	5.575397	0.243092
H	1.835149	7.242969	0.459850
H	4.964441	2.241531	-2.558614
H	3.945916	1.594127	-4.721791
H	2.019888	1.827142	-6.253417
H	-0.177493	2.583457	-5.378533
H	-0.404351	3.153587	-2.975688
H	1.517352	2.927128	-1.460875
C	3.298764	1.809404	5.447293
H	3.220342	2.401396	6.361778
H	3.089063	0.767386	5.709045
H	4.337151	1.857792	5.113020
C	3.588630	1.235397	2.527085
H	4.075203	2.205910	2.395334
H	4.210189	0.626998	3.186579
H	3.564422	0.740496	1.555244

30. PRC3

Imaginary Frequencies = 0 (Equilibrium Geometry)

Energy (Hartree) = -3797.7797206

C	-6.489789	-4.894820	-0.286303
C	-5.595165	-5.915027	-0.330010
C	-4.196673	-5.660862	-0.352621
C	-3.740859	-4.313318	-0.328214
C	-4.695566	-3.257700	-0.264887
C	-6.040293	-3.546257	-0.252861
H	-7.557984	-5.073785	-0.272406
H	-5.915159	-6.949510	-0.351530
C	-2.330563	-4.116949	-0.372341
H	-4.377496	-2.223865	-0.208270
C	-1.516212	-5.222328	-0.438520
C	-2.080000	-6.509987	-0.443998
H	-0.436969	-5.126577	-0.489757
H	-1.431045	-7.379999	-0.487140
N	-3.370229	-6.734364	-0.404087
O	-7.026884	-2.636636	-0.212527
C	-6.670854	-1.261474	-0.144831
H	-6.088681	-0.964466	-1.023100
H	-7.611226	-0.714575	-0.131348
H	-6.101655	-1.045144	0.762972
C	-1.722583	-2.727014	-0.403567
H	-2.501502	-2.013405	-0.656155
N	-0.728286	-2.679238	-1.475493
H	0.160392	-3.209757	-1.309416
C	-1.051337	-2.204937	0.901182
H	-0.367494	-1.425442	0.562997

C	-0.287850	-3.198100	1.791209
C	-1.289283	-0.518739	2.633706
C	-2.838178	-2.423778	2.634448
C	-0.451216	-2.776631	3.261221
H	-0.684129	-4.208711	1.682264
H	0.755089	-3.231262	1.481442
C	-0.194634	-1.266143	3.438665
H	-0.865172	0.236491	1.974321
H	-2.023705	-0.043002	3.282488
C	-1.901108	-3.062369	3.672859
H	-3.292570	-3.163880	1.979324
H	-3.637925	-1.823374	3.063702
H	0.240929	-3.342916	3.885379
H	-0.335573	-1.020341	4.494859
H	-2.079258	-4.138308	3.715529
H	-2.096500	-2.655617	4.667610
N	-2.041825	-1.491543	1.773726
C	1.195426	-0.849324	3.046157
H	1.541250	-1.148523	2.059651
C	2.005298	-0.129640	3.811438
H	1.705562	0.204827	4.801012
H	2.990830	0.154818	3.459546
C	-0.696147	-1.840422	-2.469129
C	0.110574	-1.352565	-4.774538
C	-1.352227	0.245283	-3.501402
C	-0.373390	0.083847	-4.621805
C	-1.606220	-0.744466	-2.613739
C	0.456826	-1.951482	-3.418671
H	0.984507	-1.389368	-5.426648
H	-0.845979	0.431376	-5.545024
H	-2.360305	-0.573454	-1.856127
H	0.764974	-2.994047	-3.502254
H	-0.672497	-1.953721	-5.246855
H	0.464107	0.774051	-4.460180
H	1.284534	-1.409448	-2.944977
C	-1.978983	1.591969	-3.250247
C	3.349743	2.576272	-0.689990
C	4.438388	0.697310	-2.530899
C	2.542029	1.888103	-1.607438
C	4.706987	2.213760	-0.687956
C	5.247704	1.301518	-1.577303
C	3.088745	0.984797	-2.521161
H	5.369679	2.663353	0.040205
H	6.301718	1.055334	-1.522168
H	2.419728	0.490322	-3.214585
H	4.844686	-0.019760	-3.233956
C	2.908192	3.665894	0.310941
C	1.514822	4.266745	0.057315
H	1.404409	4.610872	-0.974771
H	1.389371	5.135220	0.710159
H	0.702583	3.579475	0.290503
C	2.945092	3.076575	1.731839
H	3.938794	2.695002	1.974832
H	2.233256	2.254442	1.827347
H	2.692284	3.850645	2.464304
C	3.898677	4.847706	0.224829
H	3.929979	5.255974	-0.789223
H	4.914546	4.573445	0.513076
H	3.573982	5.642047	0.902317
N	1.119042	1.983282	-1.614637
C	0.292366	1.412636	-0.611467
C	0.339925	2.573189	-2.579916
C	-0.995324	1.608395	-0.871369
H	-1.812512	1.228445	-0.279168
C	-1.119358	2.339940	-2.170062
H	-1.593776	3.317267	-2.037106
O	0.752038	3.167438	-3.555567
O	0.862926	0.845103	0.447714
N	4.154322	-1.332250	0.582691
C	3.821901	-2.473559	-0.240806
C	2.317285	-2.463955	-0.551431
O	1.725272	-1.358465	-0.514379
H	3.843565	-0.420207	0.283515
H	4.038672	-3.367724	0.346816
C	4.660215	-2.519279	-1.523739
C	6.241225	-2.503119	-3.839851
C	6.006915	-2.159864	-1.466151
C	4.126364	-2.901018	-2.752115
C	4.907796	-2.885109	-3.903177
C	6.790907	-2.151008	-2.611256
H	6.447773	-1.876510	-0.517977
H	3.094347	-3.221702	-2.811679
H	4.468950	-3.174719	-4.851599
H	7.834628	-1.864953	-2.544359
H	6.850266	-2.487798	-4.736441
O	1.779861	-3.553534	-0.856263
C	5.003152	-1.416948	1.633968
O	5.149036	-0.204879	2.201398
O	5.543325	-2.440528	2.001531
C	6.148565	0.019096	3.244012
C	5.996540	1.504436	3.546785
H	6.187573	2.100690	2.651207
H	6.709473	1.800011	4.319636
H	4.987515	1.723820	3.903642
C	5.832557	-0.811961	4.484767
H	6.515075	-0.527293	5.289810
H	5.945279	-1.875331	4.282974
H	4.810218	-0.621421	4.820242
C	7.541107	-0.274023	2.692595
H	7.706986	0.292354	1.772112
H	7.665938	-1.336255	2.485732
H	8.293457	0.031753	3.423767
H	1.381996	0.054761	0.151718
H	-2.696960	-0.923667	1.175208
C	-2.212447	2.443921	-4.494803
H	-1.272909	2.754755	-4.953005
H	-2.807434	1.898106	-5.231382
H	-2.762823	3.344163	-4.217715
H	-2.943558	1.414257	-2.763095
N	-3.900797	2.919449	0.274165

H	-3.612865	2.326887	-0.487087
C	-4.127053	2.280777	1.548413
C	-4.198280	0.742564	1.368201
O	-4.765812	0.100360	2.254584
O	-3.579070	0.250783	0.368728
C	-3.023037	2.527961	2.575977
C	-1.745453	2.931815	2.202304
C	-0.726258	3.018833	3.143624
C	-0.975452	2.712260	4.475290
C	-2.258898	2.337245	4.862025
C	-3.275096	2.249156	3.919195
C	-3.794858	4.265301	0.158533
O	-4.036947	5.044209	1.054210
O	-3.385462	4.597942	-1.085652
C	-2.964226	5.968785	-1.381190
C	-2.536437	5.903348	-2.842945
C	-4.133349	6.937080	-1.222694
C	-1.775068	6.333876	-0.496480
H	-4.442946	7.013349	-0.182695
H	-3.832103	7.925260	-1.579860
H	-4.982167	6.600085	-1.823463
H	-3.361209	5.551074	-3.466974
H	-2.249035	6.900405	-3.183775
H	-1.678738	5.240949	-2.981679
H	-2.066915	6.394551	0.551309
H	-0.984287	5.585894	-0.603501
H	-1.373117	7.301531	-0.806791
H	-5.078247	2.611550	1.969552
H	-4.267513	1.929897	4.216207
H	-2.469510	2.111067	5.901495
H	-0.179336	2.775032	5.208507
H	0.266614	3.318539	2.828581
H	-1.530170	3.156699	1.164937

31. PRC9

Imaginary Frequencies = 0 (Equilibrium Geometry)

Energy (Hartree) = -3876.40919589

C	-6.166992	-5.065269	-0.019083
C	-5.252389	-6.066503	-0.086941
C	-3.859895	-5.783406	-0.125040
C	-3.431599	-4.427300	-0.087858
C	-4.405825	-3.392553	0.010170
C	-5.744260	-3.708739	0.030219
H	-7.231114	-5.266225	0.003922
H	-5.551776	-7.106861	-0.119043
C	-2.027112	-4.200611	-0.163470
H	-4.108248	-2.354007	0.086833
C	-1.191453	-5.287076	-0.263512
C	-1.727998	-6.586500	-0.269849
H	-0.116920	-5.166235	-0.347266
H	-1.062174	-7.442133	-0.337310
N	-3.012125	-6.838501	-0.206254
O	-6.748461	-2.819047	0.091959
C	-6.417446	-1.439752	0.186339
H	-5.830934	-1.116418	-0.679869
H	-7.366902	-0.908827	0.201835
H	-5.860161	-1.234162	1.103474
C	-1.452351	-2.797220	-0.204786
H	-2.257125	-2.100582	-0.417388
N	-0.500143	-2.715964	-1.312624
H	0.428535	-3.184020	-1.167304
C	-0.748914	-2.263173	1.074869
H	-0.132332	-1.437065	0.719751
C	0.115102	-3.224696	1.904940
C	-0.998748	-0.618702	2.844404
C	-2.419417	-2.620303	2.897137
C	0.016527	-2.823997	3.386703
H	-0.235747	-4.253286	1.807184
H	1.138257	-3.201402	1.534806
C	0.193911	-1.301301	3.562059
H	-0.673627	0.176992	2.176329
H	-1.722967	-0.209576	3.547897
C	-1.387676	-3.197891	3.880362
H	-2.851288	-3.385936	2.255598
H	-3.236885	-2.081799	3.373090
H	0.777068	-3.351992	3.962911
H	0.114290	-1.071239	4.628119
H	-1.496645	-4.282623	3.931538
H	-1.551190	-2.801891	4.885218
N	-1.733894	-1.626970	2.010679
C	1.524047	-0.796304	3.075311
H	1.811627	-1.058170	2.059829
C	2.347100	-0.042576	3.792472
H	2.104944	0.257297	4.808566
H	3.286143	0.303507	3.374601
C	-0.569942	-1.922973	-2.341941
C	0.101235	-1.464177	-4.688934
C	-1.450190	0.064879	-3.419257
C	-0.499499	-0.068893	-4.570456
C	-1.574106	-0.914942	-2.493232
C	0.550883	-1.983931	-3.331194
H	0.947732	-1.451203	-5.377321
H	-1.033006	0.191475	-5.488594
H	-2.310284	-0.804228	-1.712855
H	0.932318	-3.003441	-3.392950
H	-0.645203	-2.147132	-5.106398
H	0.281765	0.695550	-4.475600
H	1.355609	-1.368102	-2.911806
C	-2.173584	1.385286	-3.201269
C	3.211014	2.665927	-0.818706
C	4.302127	0.870083	-2.739001
C	2.393553	1.940006	-1.697012
C	4.585240	2.382084	-0.896258
C	5.127609	1.511364	-1.824178
C	2.941822	1.080887	-2.652092
H	5.260259	2.864185	-0.200999
H	6.195367	1.326483	-1.829857
H	2.265720	0.560936	-3.318853

H	4.709053	0.184931	-3.472921
C	2.772605	3.725841	0.214113
C	1.330901	4.238701	0.059590
H	1.132506	4.584626	-0.958645
H	1.193147	5.091127	0.730906
H	0.580926	3.498876	0.333816
C	2.947833	3.138621	1.625437
H	3.975141	2.808314	1.791966
H	2.288836	2.280486	1.770549
H	2.711241	3.897143	2.379249
C	3.679457	4.966980	0.062936
H	3.614458	5.374663	-0.949742
H	4.728067	4.756838	0.278624
H	3.353672	5.740649	0.763467
N	0.967738	1.958310	-1.639519
C	0.204307	1.365478	-0.603069
C	0.127230	2.497697	-2.583073
C	-1.100912	1.486417	-0.824186
H	-1.862109	1.088818	-0.172304
C	-1.308852	2.181850	-2.135057
H	-1.830423	3.134911	-1.999577
O	0.486754	3.116435	-3.563971
O	0.833517	0.857769	0.453994
N	4.414653	-1.107964	0.459460
C	4.093215	-2.273207	-0.332622
C	2.573058	-2.336801	-0.549226
O	1.933038	-1.261248	-0.477007
H	3.987132	-0.229753	0.207233
H	4.388106	-3.150939	0.245989
C	4.850903	-2.292894	-1.666003
C	6.275044	-2.235393	-4.081537
C	6.162124	-1.819702	-1.710302
C	4.275759	-2.767093	-2.842829
C	4.978316	-2.730658	-4.043010
C	6.868454	-1.790181	-2.904797
H	6.635242	-1.460752	-0.804391
H	3.273656	-3.175334	-2.821909
H	4.507089	-3.093799	-4.949717
H	7.885567	-1.414569	-2.916959
H	6.822663	-2.204210	-5.016546
O	2.070983	-3.453623	-0.815600
C	5.322382	-1.131543	1.461953
O	5.403749	0.086280	2.031895
O	5.961628	-2.110880	1.789407
C	6.429371	0.380841	3.030043
C	6.176447	1.847876	3.354624
H	6.284906	2.464390	2.458980
H	6.895504	2.191308	4.101607
H	5.168823	1.986490	3.753613
C	6.235203	-0.480701	4.275086
H	6.929495	-0.149727	5.051826
H	6.420463	-1.530659	4.057032
H	5.217408	-0.372269	4.657760
C	7.814121	0.200835	2.414297
H	7.895092	0.787398	1.495093
H	8.009630	-0.846594	2.187711
H	8.572961	0.556075	3.115852
H	1.435935	0.131337	0.158125
H	-2.457604	-1.089525	1.462837
N	-3.943832	2.681761	0.676951
H	-3.685982	2.097251	-0.100404
C	-4.024608	2.048458	1.970318
C	-4.023508	0.507383	1.803750
O	-4.494946	-0.160974	2.725050
O	-3.445964	0.045017	0.764616
C	-2.850903	2.362719	2.894583
C	-1.626133	2.799917	2.401205
C	-0.537546	2.955929	3.251180
C	-0.661567	2.678435	4.606603
C	-1.890357	2.262404	5.110537
C	-2.977873	2.109926	4.260050
C	-3.902394	4.027299	0.529130
O	-4.098843	4.820797	1.422838
O	-3.614953	4.339464	-0.754677
C	-3.339653	5.722620	-1.140720
C	-3.032599	5.619432	-2.630679
C	-4.572367	6.594908	-0.919138
C	-2.117429	6.229199	-0.379680
H	-4.807153	6.680049	0.139893
H	-4.385501	7.592530	-1.324701
H	-5.432271	6.168057	-1.442076
H	-3.858974	5.136308	-3.157880
H	-2.895099	6.619822	-3.046830
H	-2.116520	5.052553	-2.812525
H	-2.328579	6.324488	0.684706
H	-1.279375	5.539675	-0.516203
H	-1.823320	7.206332	-0.770958
H	-4.952958	2.330040	2.470624
H	-3.928806	1.763105	4.648321
H	-2.002852	2.056509	6.169292
H	0.189966	2.794482	5.267525
H	0.411796	3.285690	2.846995
H	-1.509723	2.999152	1.342306
C	-3.584136	1.202073	-2.589379
H	-3.979886	2.207396	-2.410008
H	-3.520262	0.722805	-1.609474
C	-2.295611	2.215234	-4.486628
H	-2.839892	1.666125	-5.258028
H	-2.853261	3.129121	-4.270501
H	-1.325247	2.509582	-4.882016
C	-4.567690	0.411915	-3.448988
H	-5.521130	0.311379	-2.925369
H	-4.768298	0.905041	-4.402482
H	-4.197381	-0.595452	-3.659269

32. Salt_Bridge_N-Boc-L-Phenylglycine

N	3.129000	-2.915000	2.804000
H	2.877000	-2.663000	1.855000
C	3.246000	-1.814000	3.734000

C	3.798000	-0.565000	3.013000
O	4.808000	-0.001000	3.558000
O	3.263000	-0.219000	1.949000
C	1.906000	-1.442000	4.372000
C	0.744000	-2.131000	4.004000
C	-0.473000	-1.839000	4.611000
C	-0.552000	-0.846000	5.586000
C	0.594000	-0.138000	5.934000
C	1.817000	-0.438000	5.335000
C	3.820000	-4.079000	2.954000
O	4.769000	-4.240000	3.698000
O	3.273000	-5.016000	2.152000
C	2.561000	-6.153000	2.717000
C	1.648000	-6.578000	1.573000
C	3.538000	-7.274000	3.062000
C	1.738000	-5.714000	3.923000
H	4.438000	-6.853000	3.460000
H	3.095000	-7.922000	3.789000
H	3.766000	-7.833000	2.178000
H	2.134000	-7.333000	0.990000
H	0.735000	-6.968000	1.972000
H	1.434000	-5.732000	0.954000
H	2.281000	-4.976000	4.476000
H	0.810000	-5.299000	3.589000
H	1.545000	-6.559000	4.550000
H	3.929000	-2.092000	4.509000
H	2.694000	0.107000	5.617000
H	0.537000	0.641000	6.665000
H	-1.485000	-0.630000	6.062000
H	-1.352000	-2.379000	4.328000
H	0.793000	-2.888000	3.250000

33. TS1-anti

Imaginary Frequencies = 1 (Transition State)

Energy (Hartree) = -3797.75137305

C	4.224142	6.035208	-2.393089
C	3.032483	6.499882	-2.847847
C	1.844694	5.733927	-2.698742
C	1.917906	4.463621	-2.060891
C	3.174595	4.003716	-1.570152
C	4.302159	4.772326	-1.745292
H	5.138511	6.605112	-2.504817
H	2.948319	7.461157	-3.339759
C	0.702527	3.724518	-1.966586
H	3.252800	3.056739	-1.049612
C	-0.437751	4.281890	-2.493491
C	-0.396473	5.556329	-3.083613
H	-1.380058	3.747142	-2.478480
H	-1.306669	5.992884	-3.485473
N	0.698646	6.267305	-3.190078
O	5.541863	4.436417	-1.349354
C	5.713726	3.219029	-0.637424
H	5.388709	2.362154	-1.235627
H	6.780583	3.141441	-0.438487
H	5.159334	3.237160	0.303845
C	0.639168	2.333965	-1.355427
H	1.642277	1.915828	-1.310089
N	-0.152641	1.452307	-2.200880
H	-1.163916	1.459899	-2.077950
C	0.081286	2.214504	0.086081
H	-0.099373	1.144378	0.200438
C	-1.191095	2.989688	0.456687
C	0.763194	1.780443	2.372387
C	1.242223	3.984638	1.423932
C	-1.116869	3.357014	1.947620
H	-1.279190	3.912450	-0.120418
H	-2.068353	2.383514	0.229038
C	-0.681538	2.141364	2.797519
H	0.876224	0.717601	2.157947
H	1.503128	2.052534	3.123390
C	-0.052754	4.451511	2.112209
H	1.417379	4.500521	0.482538
H	2.134064	4.090399	2.039738
H	-2.087242	3.716815	2.289756
H	-0.657584	2.458002	3.843883
H	-0.395048	5.383168	1.658455
H	0.123710	4.646315	3.172551
N	1.121716	2.526131	1.122355
C	-1.612419	0.966714	2.685666
H	-1.691343	0.493334	1.710398
C	-2.312316	0.469629	3.697113
H	-2.249839	0.895151	4.695143
H	-2.968309	-0.382573	3.560627
C	0.324442	0.442732	-2.930788
C	-0.097713	-1.132152	-4.824219
C	2.038530	-1.186089	-3.479198
C	1.110431	-1.956363	-4.386371
C	1.660167	0.070687	-2.985300
C	-0.711765	-0.359711	-3.662315
H	-0.852348	-1.777539	-5.280498
H	1.677623	-2.276290	-5.265408
H	2.392931	0.645068	-2.439254
H	-1.501646	0.307593	-4.012874
H	0.213900	-0.416569	-5.591222
H	0.802611	-2.882839	-3.891446
H	-1.181956	-1.029795	-2.935473
C	3.169321	-1.817193	-2.957223
C	-1.898614	-3.899949	-0.041397
C	-3.098714	-3.865728	-2.617937
C	-1.203243	-3.430682	-1.169512
C	-3.241263	-4.244946	-0.256625
C	-3.843325	-4.218910	-1.502459
C	-1.788570	-3.466924	-2.436212
H	-3.846260	-4.547628	0.587636
H	-4.889764	-4.481282	-1.603007
H	-1.195119	-3.162147	-3.286785
H	-3.539503	-3.871300	-3.606997
C	-1.344600	-4.062259	1.392037

C	0.189477	-4.158032	1.473686
H	0.583196	-4.887484	0.760939
H	0.469386	-4.494064	2.475783
H	0.689387	-3.203953	1.313873
C	-1.830436	-2.888460	2.259804
H	-2.920335	-2.802046	2.227052
H	-1.402594	-1.944449	1.919650
H	-1.536136	-3.044211	3.302776
C	-1.878329	-5.379848	1.996503
H	-1.624867	-6.233461	1.361930
H	-2.958588	-5.374343	2.148173
H	-1.419964	-5.535907	2.976453
N	0.088849	-2.825672	-1.090244
C	0.322992	-1.567186	-0.493093
C	1.306937	-3.408819	-1.428201
C	1.715835	-1.354284	-0.420353
H	2.161398	-0.487997	0.043644
C	2.359034	-2.399892	-1.070401
H	3.349603	-2.752651	-0.824033
O	1.446961	-4.509153	-1.908523
O	-0.606782	-0.850464	-0.082213
N	-5.557530	0.282172	0.470717
C	-5.136433	0.298511	-0.910021
C	-3.614077	0.202730	-1.001923
O	-3.142634	-0.747503	-0.238775
H	-5.308115	-0.526880	1.019519
H	-5.419836	1.267247	-1.321117
C	-5.753667	-0.817835	-1.742452
C	-6.837535	-2.846485	-3.329639
C	-6.700113	-1.690366	-1.217619
C	-5.358039	-0.964257	-3.072625
C	-5.896025	-1.970915	-3.860849
C	-7.238890	-2.700495	-2.008625
H	-7.031424	-1.575712	-0.192932
H	-4.623453	-0.285134	-3.493118
H	-5.580362	-2.071504	-4.893094
H	-7.977041	-3.373454	-1.587160
H	-7.257997	-3.634105	-3.944253
O	-2.955016	0.937231	-1.714107
C	-5.670884	1.460035	1.161208
O	-5.770661	1.199549	2.467817
O	-5.694568	2.553929	0.640877
C	-5.985747	2.269345	3.445067
C	-6.000027	1.512203	4.766906
H	-6.796840	0.765220	4.772469
H	-6.168593	2.208456	5.591426
H	-5.044645	1.007163	4.926196
C	-4.829511	3.264488	3.413464
H	-4.942905	3.970176	4.240421
H	-4.811985	3.818669	2.476099
H	-3.881769	2.735794	3.536165
C	-7.331914	2.937899	3.186569
H	-8.128574	2.189892	3.170325
H	-7.327275	3.474930	2.238679
H	-7.543518	3.647158	3.990552
H	-2.142122	-0.840177	-0.280356
H	2.051804	2.144696	0.800491
N	4.727470	-0.944612	1.275136
H	4.369905	-0.741356	0.355255
C	4.473617	0.040168	2.296899
C	3.952641	1.341805	1.636590
O	4.042744	2.388992	2.280450
O	3.377127	1.207718	0.507956
C	3.400326	-0.361775	3.304438
C	2.454782	-1.341011	3.016460
C	1.406788	-1.588272	3.895984
C	1.293409	-0.862604	5.075027
C	2.249607	0.101078	5.379403
C	3.297318	0.345938	4.501211
C	5.222391	-2.174448	1.546649
O	5.592189	-2.541306	2.640284
O	5.242835	-2.916813	0.416298
C	5.466145	-4.360919	0.463490
C	5.309854	-4.786074	-0.993541
C	6.877793	-4.663734	0.956472
C	4.390885	-5.014433	1.327784
H	7.003311	-4.359975	1.993958
H	7.067158	-5.737025	0.872794
H	7.610576	-4.136935	0.339703
H	5.996661	-4.223833	-1.630880
H	5.541281	-5.849198	-1.090422
H	4.287728	-4.632568	-1.348362
H	4.492355	-4.719762	2.371862
H	3.396577	-4.730487	0.970861
H	4.479464	-6.101362	1.258109
H	5.394391	0.275928	2.834185
H	4.027216	1.115602	4.725005
H	2.179738	0.664518	6.303360
H	0.470908	-1.052121	5.755363
H	0.672778	-2.346404	3.652143
H	2.521630	-1.899485	2.090149
C	4.363104	-1.056637	-2.430524
H	4.916334	-0.593575	-3.253765
H	4.077672	-0.258436	-1.738919
H	5.044526	-1.726407	-1.902062
H	3.397660	-2.798672	-3.368641

34. TS1-anti_pre-docking

Imaginary Frequencies = 1 (Transition State)

Energy (Hartree) = -3797.7446376

C	5.069726	4.709989	-2.133857
C	4.075404	5.242151	-2.889226
C	2.763470	4.697131	-2.859464
C	2.502653	3.564507	-2.036824
C	3.557149	3.030556	-1.238989
C	4.809674	3.598560	-1.287996
H	6.073313	5.117493	-2.142622
H	4.245875	6.097032	-3.532029

C	1.177453	3.037311	-2.075931
H	3.377595	2.185836	-0.584532
C	0.257771	3.663896	-2.882694
C	0.621128	4.794539	-3.632926
H	-0.754075	3.287479	-2.970914
H	-0.119054	5.286027	-4.258405
N	1.829140	5.300365	-3.635833
O	5.878668	3.194660	-0.575519
C	5.680029	2.214581	0.430428
H	5.339225	1.269141	-0.001192
H	6.645511	2.067964	0.908885
H	4.951652	2.559873	1.169046
C	0.755495	1.790334	-1.309948
H	1.636163	1.208994	-1.040362
N	-0.079763	0.948102	-2.156181
H	-1.082913	1.119783	-2.145555
C	-0.027495	1.993848	0.015048
H	-0.414766	0.995643	0.222565
C	-1.172458	3.016545	0.050937
C	0.190241	1.789009	2.415826
C	1.213156	3.721192	1.327862
C	-1.272715	3.576592	1.479817
H	-0.986492	3.848098	-0.632803
H	-2.102138	2.543748	-0.265648
C	-1.209812	2.444584	2.531431
H	0.132265	0.702330	2.337437
H	0.848389	2.036616	3.246618
C	-0.063022	4.493899	1.709516
H	1.631398	4.060655	0.382595
H	1.996085	3.767620	2.083756
H	-2.202174	4.134663	1.595465
H	-1.288679	2.907560	3.519224
H	-0.147609	5.392599	1.096017
H	-0.024509	4.810827	2.754384
N	0.872446	2.277400	1.177871
C	-2.310236	1.430505	2.403481
H	-2.364060	0.875383	1.471530
C	-3.185213	1.150889	3.359965
H	-3.164161	1.664455	4.317536
H	-3.953298	0.399688	3.219304
C	0.287813	-0.190225	-2.745525
C	-0.235173	-1.865345	-4.525969
C	1.755271	-2.108405	-2.991229
C	0.784999	-2.815485	-3.905912
C	1.545032	-0.765822	-2.637916
C	-0.803429	-0.895503	-3.497446
H	-1.047001	-2.430486	-4.990463
H	1.357648	-3.310220	-4.695834
H	2.314367	-0.249339	-2.084157
H	-1.449293	-0.155549	-3.974039
H	0.246725	-1.288096	-5.321089
H	0.293639	-3.626201	-3.358548
H	-1.427405	-1.411319	-2.760787
C	2.738009	-2.851376	-2.336375
C	-2.754049	-3.738756	0.386229
C	-3.762822	-3.837866	-2.269466
C	-1.917573	-3.534140	-0.725775
C	-4.119632	-3.882427	0.098670
C	-4.627191	-3.919149	-1.187915
C	-2.418461	-3.635668	-2.024941
H	-4.820750	-3.970127	0.917896
H	-5.694644	-4.017516	-1.345767
H	-1.728304	-3.542502	-2.851742
H	-4.133370	-3.899603	-3.285192
C	-2.328875	-3.817353	1.869817
C	-0.843297	-4.150171	2.089673
H	-0.526635	-5.001180	1.480247
H	-0.699505	-4.422891	3.137738
H	-0.174534	-3.311651	1.902287
C	-2.657995	-2.488671	2.572985
H	-3.722612	-2.251714	2.486794
H	-2.089379	-1.663626	2.142547
H	-2.408612	-2.558495	3.636684
C	-3.117535	-4.951749	2.561596
H	-2.963982	-5.905147	2.048395
H	-4.190179	-4.759680	2.613173
H	-2.763133	-5.057855	3.590198
N	-0.545903	-3.152580	-0.608478
C	-0.120874	-1.906575	-0.097098
C	0.560203	-3.969266	-0.820355
C	1.284757	-1.945816	0.073988
H	1.846209	-1.136972	0.518230
C	1.758094	-3.135609	-0.461495
H	2.651453	-3.653856	-0.141270
O	0.522136	-5.109486	-1.216630
O	-0.933238	-1.003382	0.170054
N	-5.684795	0.942481	0.200869
C	-5.220225	0.677808	-1.137594
C	-3.733460	0.328850	-1.134848
O	-3.434380	-0.556638	-0.220980
H	-5.613578	0.208871	0.888395
H	-5.319379	1.609520	-1.694644
C	-5.998017	-0.424365	-1.846830
C	-7.389024	-2.424083	-3.215935
C	-7.099993	-1.038769	-1.263330
C	-5.599863	-0.815087	-3.125686
C	-6.290382	-1.807470	-3.805788
C	-7.791813	-2.034918	-1.945453
H	-7.432747	-0.730987	-0.279679
H	-4.743418	-0.338495	-3.591522
H	-5.970934	-2.099401	-4.799774
H	-8.650436	-2.504889	-1.479405
H	-7.929163	-3.199854	-3.746293
O	-2.948454	0.840322	-1.910864
C	-5.765881	2.225525	0.667431
O	-6.061045	2.207435	1.969839
O	-5.612416	3.210411	-0.022542
C	-6.273914	3.446506	2.720664
C	-6.541775	2.937729	4.131379
H	-7.413221	2.279413	4.142250

H	-6.732905	3.780354	4.799544
H	-5.679378	2.382905	4.507003
C	-5.020127	4.316184	2.695040
H	-5.162602	5.166490	3.366814
H	-4.820930	4.689467	1.691502
H	-4.158678	3.741212	3.041017
C	-7.493526	4.177795	2.168642
H	-8.360392	3.512288	2.158677
H	-7.309821	4.539273	1.157555
H	-7.724066	5.031722	2.810448
H	-2.458476	-0.794967	-0.185603
H	1.766253	1.694007	1.081507
C	4.004798	-2.227968	-1.807397
H	4.677909	-1.959191	-2.627005
H	3.808971	-1.313032	-1.242804
H	4.549711	-2.913419	-1.155217
H	2.832002	-3.890091	-2.647314
N	4.919099	-1.102705	1.533723
C	4.247550	-0.499010	2.660921
C	3.426422	0.727921	2.210044
O	2.921491	0.648556	1.041815
H	4.356701	-1.219664	0.704460
H	5.014963	-0.157044	3.355893
C	3.311911	-1.473936	3.370592
C	1.555429	-3.218428	4.677177
C	2.509490	-1.020300	4.419881
C	3.226108	-2.809221	2.989262
C	2.352571	-3.676122	3.637099
C	1.637958	-1.885657	5.067053
H	2.571570	0.019056	4.721778
H	3.849119	-3.173824	2.181701
H	2.291434	-4.711335	3.320103
H	1.022489	-1.518047	5.880697
H	0.872730	-3.893755	5.180473
O	3.278663	1.668778	2.995209
C	6.255065	-0.946244	1.324754
O	6.543793	-1.300706	0.060460
O	7.049407	-0.556042	2.153138
C	7.914334	-1.312862	-0.442693
C	7.737993	-1.793670	-1.877981
H	7.275162	-2.782921	-1.896951
H	8.710196	-1.852145	-2.372504
H	7.103574	-1.101330	-2.436288
C	8.502599	0.094926	-0.421000
H	9.477884	0.084335	-0.914290
H	8.630533	0.450242	0.600613
H	7.854024	0.787754	-0.963160
C	8.755916	-2.302063	0.357779
H	8.279209	-3.285706	0.360767
H	8.883635	-1.965524	1.385760
H	9.740345	-2.399326	-0.106803

35. TS1-syn

Imaginary Frequencies = 1 (Transition State)

Energy (Hartree) = -3797.73811248

C	-0.394899	-1.607751	-1.635082
C	0.005360	-1.356319	-0.135615
C	0.079296	-2.917331	-2.250688
C	1.273088	-2.028786	0.448432
N	-1.138588	-1.732719	0.769527
H	0.083250	-0.273282	-0.034154
N	0.026804	-0.522725	-2.510174
H	-1.489188	-1.634948	-1.665376
C	1.438429	-3.173761	-2.630014
C	-0.848690	-3.893458	-2.500334
C	0.911205	-2.889425	1.662630
H	1.762569	-2.669725	-0.276682
H	1.999624	-1.266468	0.727939
C	-1.408247	-3.202975	0.826686
C	-0.879069	-1.235876	2.154235
C	-0.542820	0.667713	-2.723809
H	0.887234	-0.672395	-3.010030
C	2.504590	-2.253462	-2.480935
C	1.727278	-4.453725	-3.178119
C	-0.448510	-5.127116	-3.059802
H	-1.896651	-3.717074	-2.280675
C	-0.117391	-3.934097	1.217831
C	0.297098	-2.022293	2.781086
H	1.810389	-3.382197	2.030980
H	-1.810478	-3.511074	-0.133296
H	-2.200105	-3.333601	1.565434
H	-0.659887	-0.171425	2.073299
H	-1.806543	-1.368296	2.712278
C	0.233011	1.529730	-3.686103
C	-1.727509	1.104237	-2.155246
C	3.786604	-2.580450	-2.850160
H	2.369195	-1.278911	-2.026315
C	3.063094	-4.766052	-3.528438
N	0.785196	-5.415721	-3.380385
H	-1.194730	-5.895642	-3.242739
H	0.275828	-4.495487	0.366320
H	-0.320362	-4.646205	2.019984
C	1.298397	-1.096819	3.412064
H	-0.087691	-2.694782	3.554139
C	-0.637012	2.588928	-4.348498
H	1.055812	1.979364	-3.119987
H	0.696960	0.890803	-4.443234
C	-2.253413	2.386041	-2.440472
H	-2.283016	0.441153	-1.502050
C	4.077095	-3.862970	-3.371340
O	4.715792	-1.609186	-2.695115
H	3.252654	-5.753894	-3.930443
C	1.684674	-1.163375	4.679959
H	1.724458	-0.330164	2.767428
C	-1.467709	3.337137	-3.312457
H	-0.009964	3.286937	-4.907716
H	-1.306374	2.104994	-5.066911

C	-3.441765	2.834840	-1.871395	H	4.371971	-0.482959	-0.781745
H	5.088069	-4.131255	-3.648445	H	4.533824	0.960723	-2.769466
C	6.088047	-1.968524	-2.641507	C	5.039405	-1.309976	1.457373
H	1.277203	-1.905889	5.360638	H	4.616040	0.607969	1.941000
H	2.420691	-0.475361	5.080870	O	2.584641	1.404593	1.055438
H	-0.815926	3.960058	-2.689860	O	2.202879	0.865274	-1.083914
H	-2.154272	4.028321	-3.804749	O	5.224149	-1.435158	2.777198
H	6.256102	-2.736626	-1.882553	O	5.073845	-2.205820	0.639054
H	6.451766	-2.304943	-3.616941	H	1.612610	1.656492	1.071269
H	6.615156	-1.058828	-2.357656	C	5.542246	-2.723878	3.391164
C	-2.757218	3.362191	0.105341	C	5.677995	-2.363976	4.865450
C	-2.228002	2.279362	0.784448	C	6.861347	-3.255509	2.839642
C	-1.626849	4.310743	-0.184866	C	4.390387	-3.702030	3.187037
H	-3.749484	3.769238	0.235881	H	4.742163	-1.943175	5.240303
C	-0.815161	2.417920	0.811954	H	5.916206	-3.258211	5.445705
H	-2.751820	1.425218	1.182451	H	6.475049	-1.631227	5.009575
N	-0.477243	3.658321	0.241235	H	7.645828	-2.502060	2.947086
O	-1.670179	5.412616	-0.676621	H	7.157662	-4.141915	3.405967
O	0.048384	1.671713	1.302301	H	6.767764	-3.524847	1.788490
C	0.854308	4.175326	0.211130	H	3.464947	-3.267029	3.572339
C	1.544781	3.978175	-0.982093	H	4.595845	-4.622267	3.739756
C	1.473063	4.782139	1.318935	H	4.264754	-3.946400	2.132651
C	2.869926	4.342793	-1.124565	O	-3.445470	-0.800437	0.037828
H	1.018755	3.492018	-1.790248	C	-4.054390	-1.741834	-0.564929
C	0.838671	5.032516	2.704725	H	-2.027827	-1.260749	0.418665
C	2.822584	5.121044	1.145656	C	-5.425415	-2.094876	0.054612
C	3.514083	4.910977	-0.036661	O	-3.618094	-2.421494	-1.496305
H	3.398011	4.160230	-2.052503	N	-6.040027	-0.895275	0.577524
C	1.208409	3.852941	3.623821	C	-5.099519	-3.131965	1.117153
C	-0.691798	5.197461	2.688367	H	-6.078404	-2.521282	-0.705380
C	1.399862	6.331909	3.319794	C	-7.220332	-0.433035	0.080648
H	3.365047	5.561958	1.970456	H	-5.382077	-0.192772	0.885421
H	4.560225	5.184886	-0.104658	C	-4.890614	-2.764725	2.444143
H	2.294237	3.750287	3.707740	C	-4.858570	-4.451033	0.734235
H	0.801710	4.018991	4.626553	O	-7.379286	0.850408	0.450009
H	0.805494	2.914556	3.237861	O	-8.003177	-1.086968	-0.574162
H	-1.010378	5.911298	1.924888	C	-4.438890	-3.697974	3.371620
H	-1.011949	5.583894	3.659517	H	-5.086953	-1.745019	2.753558
H	-1.222122	4.257698	2.537801	C	-4.407988	-5.384324	1.658987
H	0.878806	6.537537	4.258226	H	-5.006831	-4.738143	-0.300999
H	1.245780	7.183642	2.651476	C	-8.545214	1.623280	0.036218
H	2.462858	6.268139	3.557539	C	-4.189257	-5.008922	2.981015
C	6.776003	3.318320	-0.693814	H	-4.282020	-3.398959	4.402211
C	6.046509	2.315914	-0.063953	H	-4.226065	-6.406783	1.347273
C	6.691597	3.485658	-2.070312	C	-8.276040	2.991032	0.652888
H	7.409396	3.971930	-0.104593	C	-9.817014	1.013270	0.618601
C	5.230954	1.470453	-0.807274	C	-8.597613	1.724720	-1.485829
H	6.123184	2.191324	1.009625	H	-3.836071	-5.736017	3.703441
C	5.883589	2.635092	-2.818423	H	-7.350911	3.415769	0.255767
H	7.259211	4.268756	-2.559857	H	-9.098049	3.672241	0.421490
C	4.368028	0.404414	-0.147228	H	-8.184388	2.909702	1.738263
C	5.156838	1.632623	-2.190458	H	-9.721898	0.909226	1.702545
H	5.822675	2.750310	-3.894796	H	-10.663724	1.672294	0.409940
N	4.817604	0.002326	1.161316	H	-10.015516	0.034835	0.183828
C	2.928316	0.915966	-0.114836	H	-7.651440	2.114256	-1.870234

H	-9.395306	2.413767	-1.774995
H	-8.790718	0.752726	-1.937113
C	-4.551305	1.914270	-1.431711
H	-5.048938	1.477214	-2.304713
H	-4.189256	1.090942	-0.815829
H	-5.310229	2.455821	-0.861318
H	-3.761226	3.823918	-2.192058

36. TS10-anti

Imaginary Frequencies = 1 (Transition State)

Energy (Hartree) = -3876.38136305

C	3.954374	6.109436	-2.547058
C	2.773002	6.481022	-3.102968
C	1.611291	5.675783	-2.955995
C	1.699471	4.466093	-2.210822
C	2.942679	4.106400	-1.613813
C	4.046281	4.908383	-1.792336
H	4.849580	6.709468	-2.656255
H	2.678125	7.394486	-3.677150
C	0.513067	3.680302	-2.125563
H	3.028869	3.210708	-1.010314
C	-0.615605	4.138600	-2.761721
C	-0.593084	5.362505	-3.451381
H	-1.532601	3.561423	-2.758292
H	-1.494954	5.722004	-3.939163
N	0.475350	6.113510	-3.553748
O	5.274004	4.662097	-1.304027
C	5.452904	3.518341	-0.480316
H	5.196291	2.599930	-1.017427
H	6.508898	3.505130	-0.218652
H	4.845204	3.593299	0.424440
C	0.471713	2.339958	-1.409531
H	1.486607	1.978293	-1.260799
N	-0.214924	1.356288	-2.234145
H	-1.230919	1.308153	-2.170693
C	-0.181503	2.299011	-0.005313
H	-0.308042	1.231148	0.182182
C	-1.515933	3.024478	0.216936
C	0.365534	2.086057	2.347190
C	0.782976	4.234684	1.254181
C	-1.567329	3.504086	1.676681
H	-1.609727	3.895737	-0.434569
H	-2.342137	2.354294	-0.021392
C	-1.125012	2.382355	2.643958
H	0.558382	1.019905	2.229706
H	1.031989	2.465970	3.119863
C	-0.578859	4.670075	1.824515
H	0.987235	4.685783	0.285553
H	1.626008	4.444286	1.910688
H	-2.577924	3.828354	1.925540
H	-1.196024	2.772856	3.662922
H	-0.941663	5.544792	1.282067
H	-0.483835	4.950209	2.876155
N	0.766359	2.752552	1.066144
C	-1.972605	1.144043	2.557811
H	-1.962853	0.604807	1.614212
C	-2.698650	0.664270	3.558907
H	-2.723754	1.154157	4.528666
H	-3.289944	-0.236754	3.442077
C	0.363641	0.342403	-2.874043
C	0.168796	-1.336007	-4.701822
C	2.223257	-1.183474	-3.236318
C	1.398912	-2.054347	-4.154085
C	1.724013	0.059735	-2.832336
C	-0.570814	-0.565266	-3.615919
H	-0.506262	-2.054460	-5.173116
H	2.026233	-2.386104	-4.986464
H	2.378861	0.723039	-2.290537
H	-1.386326	0.024003	-4.039637
H	0.478625	-0.631796	-5.480085
H	1.112306	-2.972075	-3.630741
H	-1.029896	-1.237239	-2.884098
C	3.384829	-1.691462	-2.616577
C	-1.733563	-3.909844	0.163163
C	-2.783536	-4.130972	-2.468235
C	-0.999349	-3.485079	-0.957423
C	-3.043276	-4.341301	-0.096553
C	-3.573487	-4.439882	-1.370495
C	-1.508396	-3.648254	-2.247630
H	-3.680125	-4.611324	0.735624
H	-4.598826	-4.762585	-1.506796
H	-0.883527	-3.374058	-3.086430
H	-3.165977	-4.233375	-3.476254
C	-1.258853	-3.946443	1.633103
C	0.267987	-3.897413	1.815234
H	0.769753	-4.637616	1.186160
H	0.506410	-4.134873	2.855738
H	0.694429	-2.916445	1.611836
C	-1.900472	-2.779164	2.403126
H	-2.989067	-2.789851	2.298323
H	-1.530749	-1.819607	2.038318
H	-1.665144	-2.857977	3.469485
C	-1.712843	-5.277066	2.273581
H	-1.336648	-6.132251	1.705458
H	-2.797303	-5.367485	2.349331
H	-1.314424	-5.341124	3.289550
N	0.250538	-2.801296	-0.857596
C	0.388299	-1.500178	-0.318860
C	1.511132	-3.334316	-1.102486
C	1.757660	-1.205635	-0.204948
H	2.126424	-0.287739	0.225722
C	2.493129	-2.242531	-0.784510
H	3.480678	-2.534832	-0.453939
O	1.726808	-4.462822	-1.482843
O	-0.602295	-0.821764	0.017874
N	-5.623355	0.024185	0.284192
C	-5.152020	-0.028554	-1.078677
C	-3.624485	-0.033979	-1.114438

O	-3.121291	-0.887605	-0.264549
H	-5.342830	-0.721733	0.902637
H	-5.477543	0.890727	-1.565421
C	-5.673262	-1.233354	-1.852076
C	-6.590426	-3.419579	-3.331617
C	-6.596715	-2.115586	-1.302955
C	-5.215788	-1.450756	-3.152280
C	-5.670907	-2.535742	-3.887008
C	-7.052695	-3.204031	-2.040285
H	-6.974529	-1.947656	-0.301945
H	-4.498159	-0.765438	-3.591490
H	-5.307767	-2.691035	-4.896617
H	-7.773980	-3.883529	-1.600525
H	-6.946395	-4.268033	-3.904606
O	-2.989368	0.685500	-1.863813
C	-5.877969	1.233137	0.874570
O	-6.030510	1.059781	2.190576
O	-5.967025	2.279898	0.270649
C	-6.403306	2.170446	3.069414
C	-6.429077	1.511287	4.442647
H	-7.150808	0.691722	4.460862
H	-6.714020	2.244181	5.200704
H	-5.442583	1.115077	4.693314
C	-5.346204	3.269982	3.025801
H	-5.572633	4.012832	3.794957
H	-5.328010	3.762487	2.054807
H	-4.361012	2.846469	3.232086
C	-7.787740	2.684214	2.687925
H	-8.508866	1.862960	2.687626
H	-7.775901	3.149001	1.702684
H	-8.112835	3.425992	3.421626
H	-2.113063	-0.917643	-0.260142
H	1.734880	2.403566	0.835623
C	4.010088	-2.967083	-3.146453
H	3.243865	-3.706328	-3.384101
H	4.615790	-3.412417	-2.352559
N	4.561339	-0.473245	1.758977
H	4.210893	-0.425076	0.815629
C	4.163073	0.587292	2.652905
C	3.619530	1.787172	1.840298
O	3.609658	2.892225	2.387184
O	3.125309	1.517656	0.697889
C	3.036284	0.204219	3.608550
C	2.163341	-0.842279	3.325925
C	1.067613	-1.086013	4.145713
C	0.831032	-0.287016	5.257362
C	1.711862	0.747568	5.556029
C	2.808878	0.987779	4.738480
C	5.056993	-1.644706	2.226581
O	5.378038	-1.835679	3.379121
O	5.142108	-2.548216	1.225724
C	5.380965	-3.961348	1.522903
C	5.312827	-4.628742	0.154137
C	6.766783	-4.152326	2.132766
C	4.264522	-4.485830	2.422280
H	6.832596	-3.684610	3.112837
H	6.972090	-5.221397	2.231851
H	7.526491	-3.716931	1.478279
H	6.027934	-4.166235	-0.530855
H	5.565173	-5.687079	0.251280
H	4.310706	-4.560821	-0.275615
H	4.306108	-4.030486	3.411058
H	3.290970	-4.273100	1.971141
H	4.362018	-5.569086	2.528559
H	5.020416	0.930212	3.234465
H	3.481511	1.809585	4.956664
H	1.544876	1.369738	6.428437
H	-0.029150	-0.473911	5.890144
H	0.392213	-1.897873	3.905924
H	2.324048	-1.458289	2.448238
C	4.900962	-2.723111	-4.369649
H	5.315298	-3.665309	-4.736599
H	4.339720	-2.260336	-5.186609
H	5.737337	-2.062820	-4.126723
C	4.396889	-0.727912	-2.018958
H	4.902496	-0.160967	-2.807341
H	3.952799	-0.004660	-1.331992
H	5.159986	-1.284139	-1.467466

37. TS3-anti

Imaginary Frequencies = 1 (Transition State)

Energy (Hartree) = -3797.7512489399996

C	4.212311	6.051264	-2.500493
C	3.025871	6.484111	-2.997623
C	1.845476	5.706517	-2.851274
C	1.920696	4.456831	-2.173353
C	3.171700	4.030386	-1.639387
C	4.291973	4.810990	-1.810570
H	5.121223	6.630604	-2.608072
H	2.939903	7.428328	-3.521208
C	0.712523	3.704884	-2.083889
H	3.250216	3.100523	-1.088620
C	-0.422596	4.233665	-2.650398
C	-0.384255	5.490130	-3.277639
H	-1.359372	3.689643	-2.638879
H	-1.291039	5.903113	-3.710919
N	0.704448	6.210672	-3.383233
O	5.526322	4.509433	-1.373086
C	5.699282	3.326480	-0.605580
H	5.404989	2.439037	-1.173937
H	6.761644	3.276617	-0.375967
H	5.119754	3.374111	0.319396
C	0.648053	2.331700	-1.432119
H	1.651028	1.914317	-1.367672
N	-0.143370	1.428655	-2.255406
H	-1.154729	1.442298	-2.135435
C	0.071994	2.254098	0.004958
H	-0.092993	1.185187	0.150910

C	-1.216931	3.024104	0.325725	C	-1.711964	-5.357745	2.003257
C	0.716636	1.902866	2.313588	H	-1.408875	-6.197818	1.372367
C	1.178479	4.084288	1.307040	H	-2.793604	-5.403524	2.136967
C	-1.179536	3.433694	1.806937	H	-1.261327	-5.492813	2.989995
H	-1.301853	3.929238	-0.279363	N	0.192012	-2.813765	-1.114524
H	-2.082732	2.402709	0.096727	C	0.429131	-1.551011	-0.525809
C	-0.742965	2.248685	2.698086	C	1.406904	-3.402936	-1.450038
H	0.853070	0.836821	2.133746	C	1.819470	-1.331048	-0.483395
H	1.436073	2.212063	3.070085	H	2.260973	-0.449787	-0.043397
C	-0.134966	4.548417	1.961809	C	2.461915	-2.378719	-1.141763
H	1.358895	4.575321	0.353451	H	3.458313	-2.723579	-0.903143
H	2.058544	4.224124	1.932780	O	1.538701	-4.523226	-1.887916
H	-2.162318	3.787352	2.118972	O	-0.500703	-0.840855	-0.097274
H	-0.747100	2.592398	3.736208	N	-5.444539	0.245056	0.548245
H	-0.483088	5.463064	1.478850	C	-5.068358	0.231115	-0.844589
H	0.020366	4.772602	3.019712	C	-3.549452	0.149304	-0.987248
N	1.087744	2.615760	1.048112	O	-3.037110	-0.769694	-0.213684
C	-1.649294	1.053898	2.597757	H	-5.167411	-0.541959	1.114990
H	-1.713688	0.564175	1.629781	H	-5.375226	1.187491	-1.267748
C	-2.342968	0.554618	3.612102	C	-5.704145	-0.907876	-1.631991
H	-2.294941	0.994519	4.604687	C	-6.833215	-2.971969	-3.139796
H	-2.979417	-0.313439	3.484158	C	-6.628745	-1.772616	-1.057656
C	0.325859	0.412657	-2.977953	C	-5.352249	-1.080880	-2.971149
C	-0.111359	-1.121542	-4.903222	C	-5.912676	-2.105240	-3.719935
C	2.023892	-1.220760	-3.567592	C	-7.190241	-2.800399	-1.809092
C	1.102688	-1.956297	-4.502716	H	-6.925250	-1.637130	-0.024817
C	1.655701	0.017701	-3.025913	H	-4.634552	-0.408413	-3.429732
C	-0.714587	-0.380211	-3.715071	H	-5.631348	-2.226366	-4.759874
H	-0.870663	-1.756097	-5.366531	H	-7.911318	-3.466751	-1.349339
H	1.667319	-2.245647	-5.395156	H	-7.271632	-3.772972	-3.723823
H	2.404338	0.559394	-2.463670	O	-2.927754	0.870243	-1.745895
H	-1.514289	0.287721	-4.040432	C	-5.604993	1.436185	1.204028
H	0.190751	-0.385958	-5.654932	O	-5.694545	1.210187	2.517701
H	0.800692	-2.902457	-4.039980	O	-5.674067	2.512808	0.652056
H	-1.169391	-1.070794	-2.997824	C	-5.958089	2.297285	3.463038
C	3.166025	-1.843546	-3.053899	C	-5.946673	1.578259	4.806011
C	-1.784492	-3.880655	-0.031086	H	-6.712678	0.800095	4.830811
C	-2.996695	-3.905193	-2.601683	H	-6.146380	2.290330	5.609803
C	-1.096618	-3.428963	-1.170379	H	-4.972582	1.116715	4.982286
C	-3.126299	-4.238046	-0.233725	C	-4.846025	3.340574	3.407031
C	-3.734495	-4.241147	-1.476090	H	-4.994854	4.064336	4.212449
C	-1.688783	-3.494595	-2.433307	H	-4.848225	3.867892	2.454195
H	-3.725775	-4.525511	0.620014	H	-3.876377	2.858710	3.548556
H	-4.780255	-4.509976	-1.566026	C	-7.330459	2.900644	3.181939
H	-1.101822	-3.201183	-3.292279	H	-8.094545	2.119206	3.184425
H	-3.441618	-3.932467	-3.588654	H	-7.344864	3.410695	2.219342
C	-1.231182	-4.016864	1.404933	H	-7.575283	3.622307	3.965190
C	0.303775	-4.045973	1.501864	H	-2.034699	-0.849530	-0.280137
H	0.734498	-4.772790	0.808001	H	2.028523	2.236319	0.754879
H	0.587390	-4.349854	2.513323	C	3.752890	-3.089263	-3.659115
H	0.765031	-3.075448	1.326820	H	3.007297	-3.877342	-3.780511
C	-1.773792	-2.863926	2.267144	H	4.185991	-2.872827	-4.641967
H	-2.865460	-2.818106	2.221246	H	4.548773	-3.484604	-3.025862
H	-1.376871	-1.904262	1.932345	H	3.884687	-1.172199	-2.590562
H	-1.487872	-3.010397	3.313739	N	4.731379	-0.822124	1.386716

H	4.347036	-0.691579	0.464396
C	4.435580	0.203146	2.358622
C	3.911448	1.471597	1.643078
O	3.977417	2.541644	2.252995
O	3.355697	1.291805	0.511941
C	3.342405	-0.179743	3.352036
C	2.399435	-1.159506	3.056100
C	1.339006	-1.400350	3.921552
C	1.207005	-0.663968	5.092195
C	2.156414	0.304606	5.401468
C	3.218773	0.541035	4.538279
C	5.178907	-2.045413	1.761606
O	5.535715	-2.322774	2.885975
O	5.171551	-2.889707	0.707549
C	5.358820	-4.327328	0.909317
C	5.204154	-4.906529	-0.492679
C	6.759113	-4.613759	1.444691
C	4.259219	-4.858418	1.825334
H	6.886547	-4.208871	2.446510
H	6.919843	-5.694640	1.471938
H	7.509748	-4.172470	0.783709
H	5.906847	-4.429213	-1.180196
H	5.418294	-5.977424	-0.470518
H	4.188048	-4.775821	-0.872106
H	4.365215	-4.469101	2.837230
H	3.278120	-4.574480	1.433898
H	4.310114	-5.949524	1.860021
H	5.338226	0.471466	2.910260
H	3.945790	1.311941	4.767392
H	2.070925	0.877407	6.318344
H	0.374606	-0.848511	5.761755
H	0.609250	-2.160520	3.672415
H	2.479363	-1.724550	2.134085

38. TS3-syn
Imaginary Frequencies = 1 (Transition State)
Energy (Hartree) = -3797.7412923000006

C	-0.126263	-2.058427	-1.232395
C	0.287540	-1.588109	0.207217
C	0.471881	-3.369732	-1.722273
C	1.662215	-2.011563	0.783659
N	-0.753280	-2.006265	1.213455
H	0.221367	-0.499892	0.185842
N	0.140156	-1.043608	-2.241320
H	-1.210359	-2.209271	-1.207691
C	1.833947	-3.516203	-2.147150
C	-0.347187	-4.463898	-1.812052
C	1.475901	-2.803882	2.079984
H	2.219008	-2.632101	0.090751
H	2.271718	-1.127435	0.967435
C	-0.824178	-3.481623	1.427105
C	-0.479474	-1.337380	2.520984
C	-0.566896	0.051635	-2.532783
H	0.972609	-1.172097	-2.791313
C	2.789718	-2.470886	-2.171535
C	2.245823	-4.812563	-2.562173
C	0.169248	-5.703614	-2.249025
H	-1.398685	-4.379165	-1.557582
C	0.573938	-4.006256	1.781105
C	0.805514	-1.922419	3.154066
H	2.448154	-3.143527	2.435307
H	-1.256552	-3.928046	0.536601
H	-1.539310	-3.628176	2.237331
H	-0.378861	-0.271407	2.317180
H	-1.357622	-1.496586	3.147132
C	0.020760	0.875155	-3.647524
C	-1.741703	0.425613	-1.909270
C	4.081981	-2.693820	-2.580219
H	2.561775	-1.468737	-1.827544
C	3.591920	-5.013711	-2.953057
N	1.414345	-5.889953	-2.600299
H	-0.489543	-6.566088	-2.302991
H	0.990082	-4.577760	0.947373
H	0.517263	-4.672954	2.643565
C	1.710456	-0.832010	3.654322
H	0.537771	-2.564409	3.999113
C	-1.036663	1.693870	-4.377168
H	0.794933	1.509944	-3.204671
H	0.534657	0.213871	-4.351102
C	-2.476601	1.561371	-2.320060
H	-2.157942	-0.188326	-1.119474
C	4.497968	-3.990294	-2.964274
O	4.892935	-1.611249	-2.600164
H	3.878182	-6.015700	-3.248772
C	2.167700	-0.749194	4.897411
H	1.999247	-0.071533	2.930749
C	-1.941446	2.452475	-3.410217
H	-0.555060	2.390145	-5.067187
H	-1.651912	1.017062	-4.978654
C	-3.668166	1.872870	-1.671580
H	5.519321	-4.175797	-3.269668
C	6.299604	-1.801918	-2.602901
H	1.895056	-1.479998	5.653870
H	2.828781	0.054743	5.201490
H	-1.407251	3.296516	-2.956074
H	-2.771536	2.898521	-3.961196
C	-4.625031	2.920797	-2.170380
H	-4.119163	1.056329	-1.118907
H	6.603004	-2.445977	-1.773885
H	6.645173	-2.212739	-3.556232
H	6.726869	-0.809844	-2.464615
H	-5.445536	3.033792	-1.460623
H	-5.063046	2.631238	-3.132037
H	-4.141924	3.893108	-2.288968
C	-3.008299	2.663426	0.198868
C	-2.272576	1.760413	0.947185
C	-2.056309	3.728042	-0.279370
H	-4.045553	2.919393	0.365834

C	-0.901713	2.102835	0.844424
H	-2.631835	0.884866	1.462908
N	-0.795724	3.295551	0.107558
O	-2.293838	4.757981	-0.866454
O	0.095797	1.554311	1.344659
C	0.445044	3.967063	-0.114078
C	1.041451	3.718411	-1.347486
C	1.084295	4.756127	0.857562
C	2.296462	4.203587	-1.659902
H	0.501029	3.095293	-2.044113
C	0.547717	5.104843	2.262738
C	2.367443	5.209505	0.518127
C	2.970205	4.944905	-0.701177
H	2.754216	3.980726	-2.615941
C	1.180660	4.133584	3.276655
C	-0.983899	5.044152	2.400740
C	0.950490	6.547512	2.635046
H	2.928536	5.787758	1.239630
H	3.970310	5.312039	-0.899188
H	2.272286	4.197543	3.251026
H	0.847123	4.380952	4.289553
H	0.892678	3.103041	3.059227
H	-1.481566	5.600370	1.602637
H	-1.266025	5.503508	3.351815
H	-1.372615	4.026441	2.418639
H	0.503331	6.807816	3.597887
H	0.590118	7.260730	1.888534
H	2.028492	6.677132	2.741730
C	6.448193	3.740327	-1.236564
C	5.876626	2.730314	-0.470429
C	6.274524	3.756647	-2.614858
H	7.027903	4.518288	-0.752550
C	5.132222	1.725823	-1.078475
H	6.022153	2.724030	0.603228
C	5.536962	2.748267	-3.227391
H	6.718740	4.545782	-3.210717
C	4.436646	0.640827	-0.269073
C	4.969288	1.737502	-2.463430
H	5.408054	2.746382	-4.303941
N	4.991348	0.432536	1.044747
C	2.949703	0.987226	-0.200095
H	4.516550	-0.301348	-0.812970
H	4.402660	0.943312	-2.936165
C	5.380786	-0.807439	1.456767
H	4.745563	1.086596	1.770979
O	2.614752	1.569126	0.929134
O	2.185240	0.741182	-1.107055
O	5.631563	-0.772090	2.771422
O	5.490543	-1.773737	0.730473
H	1.620714	1.704698	0.987799
C	6.134233	-1.941539	3.491390
C	6.287300	-1.415005	4.912972
C	7.484710	-2.366741	2.923647
C	5.105554	-3.066352	3.449256
H	5.323684	-1.070351	5.294997
H	6.658019	-2.208856	5.565251
H	6.993624	-0.582300	4.939592
H	8.171319	-1.516471	2.907678
H	7.915310	-3.142803	3.561284
H	7.380872	-2.759729	1.913264
H	4.151461	-2.707657	3.843180
H	5.448465	-3.893626	4.075810
H	4.964164	-3.431189	2.432299
O	-3.195634	-1.342699	0.634631
C	-3.783926	-2.141606	-0.158908
H	-1.734135	-1.691891	0.896003
C	-5.302147	-1.895008	-0.329750
O	-3.266450	-3.077092	-0.775708
N	-5.591828	-0.553617	0.111531
C	-6.051734	-2.974513	0.437488
H	-5.536118	-1.992194	-1.392109
C	-6.740354	0.096786	-0.179476
H	-4.884590	-0.113817	0.681056
C	-6.493166	-2.757160	1.739016
C	-6.253556	-4.222552	-0.148000
O	-6.673860	1.358022	0.300357
O	-7.669803	-0.382732	-0.792368
C	-7.134631	-3.769664	2.443640
H	-6.343578	-1.786520	2.198381
C	-6.892565	-5.235408	0.554995
H	-5.903144	-4.398030	-1.159050
C	-7.865820	2.195694	0.387274
C	-7.336254	-5.011965	1.854425
H	-7.480041	-3.585404	3.454977
H	-7.049591	-6.200241	0.085610
C	-7.335211	3.468018	1.037938
C	-8.897686	1.520822	1.287011
C	-8.431633	2.497676	-0.998265
H	-7.840316	-5.800466	2.401836
H	-6.579337	3.940180	0.405255
H	-8.151093	4.178963	1.186564
H	-6.886153	3.242992	2.007913
H	-8.456008	1.288389	2.259583
H	-9.742165	2.196137	1.445117
H	-9.267098	0.600560	0.834681
H	-7.661606	2.927424	-1.642925
H	-9.240025	3.227443	-0.902221
H	-8.819789	1.595710	-1.467083

39. TS5-anti

Imaginary Frequencies = 1 (Transition State)

Energy (Hartree) = -3797.7419948099996

C	-1.632003	0.496693	-3.939341
C	-2.611104	1.274608	-3.404078
C	-2.434513	1.903281	-2.143224
C	-1.211651	1.710628	-1.440856
C	-0.184042	0.951186	-2.052445
C	-0.400440	0.328519	-3.254737

H	-1.763002	-0.007689	-4.889122
H	-3.557276	1.424781	-3.909933
C	-1.122449	2.255620	-0.125044
H	0.752799	0.811906	-1.541905
C	-2.202737	2.969191	0.338604
C	-3.340917	3.150386	-0.464926
H	-2.229556	3.368484	1.342975
H	-4.188334	3.710823	-0.079390
N	-3.471937	2.633034	-1.659103
O	0.486441	-0.480442	-3.870811
C	1.729744	-0.712213	-3.224391
H	1.583071	-1.164956	-2.238960
H	2.284859	-1.398295	-3.858545
H	2.286819	0.223684	-3.119870
C	0.051207	1.954944	0.801182
H	0.692575	1.205779	0.334669
N	-0.533021	1.347419	1.999912
H	-1.543442	1.277625	1.973635
C	0.969585	3.140040	1.207724
H	1.804382	2.661706	1.720933
C	0.367181	4.201171	2.160629
C	2.736214	4.614147	0.558196
C	0.708404	4.698391	-0.707486
C	0.705483	5.596121	1.622928
H	-0.713957	4.109864	2.249061
H	0.765767	4.059498	3.167260
C	2.234480	5.764164	1.475746
H	3.408543	3.940365	1.092784
H	3.284095	5.007534	-0.300625
C	0.068286	5.741384	0.235592
H	-0.040700	4.105208	-1.225486
H	1.319972	5.177880	-1.474452
H	0.319511	6.361257	2.299591
H	2.412506	6.724427	0.981213
H	-1.010515	5.578668	0.307753
H	0.221144	6.752435	-0.149613
N	1.612996	3.798364	0.042298
C	2.957020	5.777313	2.790706
H	2.941588	4.847814	3.359156
C	3.605940	6.823932	3.285169
H	3.655679	7.765129	2.744592
H	4.111281	6.777817	4.243405
C	0.044448	0.554096	2.904197
C	-0.228116	-0.625298	5.113887
C	1.848413	-0.867339	3.716094
C	0.912243	-1.543327	4.688669
C	1.389443	0.209129	2.932709
C	-0.925439	-0.000711	3.912650
H	0.176716	0.173658	5.743146
H	0.525341	-2.466945	4.243232
H	2.066268	0.662205	2.221399
H	-1.564440	-0.717197	3.387066
H	-0.953561	-1.174352	5.718204
H	1.481389	-1.861303	5.565356
H	-1.586638	0.808666	4.234251
C	3.092472	-1.454833	3.469534
C	-1.303068	-4.329435	0.211346
C	-2.961971	-3.596497	2.404520
C	-0.816698	-3.546454	1.279164
C	-2.655446	-4.676600	0.284136
C	-3.479228	-4.312257	1.340091
C	-1.630682	-3.220458	2.356750
H	-3.105203	-5.242717	-0.517827
H	-4.526047	-4.590162	1.316576
H	-1.197383	-2.648132	3.163071
H	-3.582015	-3.315019	3.247213
C	-0.437441	-4.860506	-0.950608
C	0.293165	-3.746016	-1.722717
H	-0.385940	-2.936435	-1.999378
H	0.716734	-4.164126	-2.640464
H	1.124287	-3.323166	-1.161646
C	0.586653	-5.858257	-0.378342
H	0.076142	-6.704312	0.090382
H	1.231969	-5.405313	0.373849
H	1.221341	-6.242416	-1.182969
C	-1.286672	-5.619060	-1.984941
H	-2.036870	-4.971275	-2.447909
H	-1.790533	-6.487547	-1.553599
H	-0.629307	-5.985224	-2.777384
N	0.492506	-2.970849	1.286362
C	1.614450	-3.413534	1.969316
C	0.798733	-1.769837	0.594422
C	2.707371	-2.427877	1.645738
H	3.730040	-2.775030	1.667637
C	2.195078	-1.552461	0.700589
H	2.707296	-0.747960	0.205466
O	-0.061635	-1.122424	-0.005838
O	1.676544	-4.394049	2.675096
C	4.297333	-0.664197	3.018949
H	5.098973	-1.335189	2.696307
H	4.070987	0.019590	2.199415
H	4.687165	-0.066396	3.849263
H	3.328032	-2.309765	4.100748
N	5.167257	-0.466068	-0.755364
C	4.810865	0.719757	-1.488616
C	3.762557	1.513760	-0.708578
O	3.719483	1.488284	0.508227
H	5.255713	-0.404025	0.247142
H	4.377633	0.396519	-2.434889
C	5.985100	1.649342	-1.783014
C	8.083234	3.412570	-2.315160
C	7.187957	1.536415	-1.094838
C	5.838707	2.650766	-2.742785
C	6.881987	3.527224	-3.007588
C	8.233252	2.414700	-1.360916
H	7.317251	0.750552	-0.360467
H	4.901940	2.741973	-3.281439
H	6.759324	4.297453	-3.760549
H	9.167971	2.314956	-0.820886
H	8.899311	4.094972	-2.523303

O	2.965214	2.215562	-1.464169
H	2.340688	2.823802	-0.858549
C	5.041289	-1.703402	-1.314025
O	5.314638	-2.641236	-0.398856
O	4.741382	-1.897418	-2.473164
C	5.305345	-4.066065	-0.746916
C	5.658130	-4.743774	0.572155
H	6.601327	-4.355242	0.962337
H	5.763363	-5.819278	0.413924
H	4.875399	-4.588912	1.318150
C	3.916028	-4.491438	-1.208516
H	3.888525	-5.579046	-1.312295
H	3.654865	-4.037488	-2.163066
H	3.169527	-4.201933	-0.467365
C	6.378796	-4.346403	-1.793870
H	7.351858	-3.995164	-1.440962
H	6.143620	-3.857362	-2.737964
H	6.443714	-5.424026	-1.963048
N	-5.455119	0.576746	0.058015
C	-4.729123	-0.493708	-0.576401
C	-3.351836	-0.587187	0.079083
O	-3.092952	-0.044393	1.136261
H	-5.008589	0.996589	0.859976
H	-4.562696	-0.242116	-1.627659
C	-5.433407	-1.843382	-0.518868
C	-6.701361	-4.327831	-0.429788
C	-6.338765	-2.132226	0.496052
C	-5.160650	-2.807356	-1.485885
C	-5.790768	-4.043599	-1.442367
C	-6.972903	-3.368873	0.539259
H	-6.560369	-1.376018	1.240200
H	-4.450076	-2.586374	-2.274765
H	-5.575797	-4.784261	-2.204621
H	-7.683726	-3.581592	1.329971
H	-7.199862	-5.290121	-0.399447
O	-2.527166	-1.294359	-0.640326
H	-1.597637	-1.298982	-0.263562
C	-6.296074	1.347723	-0.685744
O	-6.581763	2.476932	-0.018178
O	-6.720794	1.031166	-1.774644
C	-7.459844	3.492286	-0.593620
C	-7.422414	4.597414	0.455461
H	-7.756756	4.217681	1.423519
H	-8.078825	5.417502	0.155957
H	-6.407880	4.988816	0.565012
C	-6.901120	3.996992	-1.921632
H	-7.463785	4.880953	-2.233242
H	-6.977629	3.235008	-2.694857
H	-5.849393	4.272483	-1.813478
C	-8.872282	2.933932	-0.737639
H	-9.229014	2.555068	0.223607
H	-8.898711	2.128193	-1.470189
H	-9.546318	3.729544	-1.065428

40. TS5-syn
Imaginary Frequencies = 1 (Transition State)
Energy (Hartree) = -3797.73493845

C	0.681729	4.452786	-0.095750
C	2.707684	3.742898	1.773787
C	0.451683	3.505048	0.924152
C	1.979075	4.972089	-0.154940
C	2.979724	4.622425	0.741749
C	1.439997	3.191582	1.848533
H	2.237973	5.674957	-0.933018
H	3.970377	5.043510	0.622525
H	1.195734	2.489349	2.631868
H	3.468789	3.470372	2.495048
C	-0.405323	5.000827	-1.045166
C	-1.199494	3.903788	-1.777965
H	-0.535041	3.169051	-2.237170
H	-1.797098	4.365581	-2.568698
H	-1.896590	3.377659	-1.127866
C	-1.367432	5.870410	-0.213115
H	-0.834467	6.719098	0.225279
H	-1.826218	5.312876	0.603582
H	-2.165512	6.259273	-0.853458
C	0.201073	5.899622	-2.137038
H	0.914230	5.353797	-2.761825
H	0.698722	6.780318	-1.724233
H	-0.603517	6.257812	-2.783803
N	-0.761864	2.756132	1.027426
C	-1.853603	3.014898	1.837766
C	-1.009045	1.594402	0.243099
C	-2.862707	1.940829	1.531632
H	-3.908571	2.187147	1.634855
C	-2.347695	1.191906	0.481074
H	-2.837551	0.410305	-0.071522
O	-0.162203	1.125750	-0.520137
O	-1.954136	3.928277	2.626905
C	4.545966	-2.630880	-2.092772
C	3.696237	-2.196396	-3.060555
C	2.324012	-1.947744	-2.782948
C	1.831233	-2.165152	-1.465890
C	2.736833	-2.648949	-0.479749
C	4.057226	-2.857563	-0.781646
H	5.598823	-2.792257	-2.287637
H	4.039498	-2.010511	-4.070847
C	0.459148	-1.851914	-1.225438
H	2.381330	-2.855299	0.517497
C	-0.283556	-1.401867	-2.288438
C	0.297342	-1.258355	-3.559310
H	-1.327264	-1.142877	-2.162628
H	-0.310017	-0.906255	-4.388339
N	1.558791	-1.505197	-3.811250
O	4.986168	-3.273351	0.107167
C	4.639029	-3.270540	1.476990
H	3.893771	-4.040895	1.703504
H	5.556170	-3.483900	2.021427

H	4.254723	-2.289841	1.777672
C	-0.245576	-1.918905	0.121015
N	0.572079	-1.343557	1.185920
H	1.525409	-1.073097	0.964533
C	-0.782952	-3.298215	0.590226
H	-1.020168	-3.134572	1.644109
C	0.167894	-4.507517	0.502789
C	-2.781424	-4.563446	0.902400
C	-1.915856	-4.339692	-1.326441
C	-0.687181	-5.768043	0.298598
H	0.849713	-4.418430	-0.345198
H	0.780630	-4.570698	1.405325
C	-1.890250	-5.788477	1.268286
H	-3.060122	-3.997896	1.795687
H	-3.706259	-4.883756	0.418187
C	-1.242347	-5.716303	-1.129409
H	-1.327528	-3.696311	-1.975974
H	-2.902342	-4.424542	-1.783375
H	-0.082124	-6.664271	0.447178
H	-2.455174	-6.705228	1.074160
H	-0.435488	-5.849618	-1.853994
H	-1.957539	-6.529301	-1.283226
N	-2.086891	-3.647508	-0.027178
C	-1.482542	-5.796939	2.713960
H	-0.992580	-4.899664	3.091172
C	-1.681593	-6.812335	3.544987
H	-2.172222	-7.724140	3.215637
H	-1.364049	-6.769416	4.580898
C	0.082038	-0.756875	2.280929
C	0.509728	0.250103	4.546050
C	-1.731665	0.370487	3.432024
C	-0.749171	1.082386	4.328796
C	-1.274340	-0.621125	2.550237
C	1.113737	-0.199345	3.221231
H	0.253123	-0.631909	5.141543
H	-0.499044	2.060042	3.900328
H	-1.992856	-1.103122	1.904205
H	1.633916	0.610664	2.706067
H	1.249278	0.818695	5.113941
H	-1.231606	1.297030	5.285514
H	1.870866	-0.971630	3.384416
C	-3.033384	0.865571	3.306205
H	-1.122950	-1.274505	0.016093
H	-3.290556	1.669624	3.993026
C	-4.200326	-0.007794	2.907099
H	-3.995315	-0.598547	2.010571
H	-4.449045	-0.706612	3.711657
H	-5.086084	0.605709	2.718902
N	-5.538377	0.371280	-0.504934
C	-5.494559	-0.603627	-1.563284
C	-4.252101	-1.488587	-1.467521
O	-3.681320	-1.901597	-2.451639
H	-5.369905	0.084264	0.445288
H	-5.418022	-0.052499	-2.500506
C	-6.723928	-1.511541	-1.625615
C	-8.943809	-3.199898	-1.783622
C	-6.856015	-2.407464	-2.687344
C	-7.712418	-1.465409	-0.649458
C	-8.817962	-2.306688	-0.728286
C	-7.958604	-3.246536	-2.765266
H	-6.087835	-2.445680	-3.452182
H	-7.629218	-0.761361	0.169838
H	-9.583303	-2.258778	0.038129
H	-8.051670	-3.935311	-3.597273
H	-9.806378	-3.853500	-1.845460
O	-3.911360	-1.781106	-0.228921
H	-3.131452	-2.467955	-0.207032
C	-5.797307	1.684109	-0.758026
O	-5.834827	2.368434	0.397022
O	-5.970988	2.142738	-1.865034
C	-5.999584	3.826628	0.408058
C	-5.911969	4.173456	1.890692
H	-6.642011	3.595658	2.462072
H	-6.125772	5.235584	2.028953
H	-4.912840	3.979540	2.288096
C	-4.862612	4.491742	-0.361758
H	-4.908868	5.572719	-0.208116
H	-4.926372	4.282264	-1.428274
H	-3.897637	4.140862	0.009734
C	-7.372427	4.192769	-0.146443
H	-8.155180	3.671355	0.410526
H	-7.451066	3.932629	-1.200797
H	-7.530734	5.268366	-0.035422
N	5.450702	0.080446	0.016132
C	4.620207	0.841988	-0.874415
C	3.194591	0.795019	-0.328544
O	2.942164	0.314857	0.761850
H	5.105291	-0.094386	0.945884
H	4.600734	0.351987	-1.852970
C	5.082603	2.285824	-1.071057
C	5.921426	4.922292	-1.455675
C	5.988550	2.870654	-0.193412
C	4.598406	3.030124	-2.144893
C	5.010666	4.341882	-2.332872
C	6.410472	4.181773	-0.387182
H	6.377602	2.294502	0.637817
H	3.889731	2.581253	-2.830966
H	4.626447	4.909967	-3.172571
H	7.123776	4.623056	0.299923
H	6.250537	5.943920	-1.608190
O	2.322242	1.298301	-1.152204
H	1.386194	1.282458	-0.788998
C	6.619559	-0.476296	-0.388600
O	7.203606	-1.098896	0.646209
O	7.053049	-0.396565	-1.518336
C	8.465257	-1.815656	0.479244
C	8.700206	-2.407334	1.863852
H	8.723256	-1.618054	2.618578
H	9.654446	-2.938402	1.884469
H	7.906720	-3.113355	2.119341

C	8.319974	-2.926267	-0.557645
H	9.206013	-3.565666	-0.525239
H	8.219609	-2.514348	-1.560828
H	7.438664	-3.530903	-0.333865
C	9.574987	-0.831013	0.122210
H	9.632007	-0.037303	0.871740
H	9.399778	-0.384911	-0.855795
H	10.533822	-1.355450	0.103546

41. TS7-anti

Imaginary Frequencies = 1 (Transition State)

Energy (Hartree) = -3797.7439067499995

C	-0.993289	1.062823	-4.406095
C	-2.090205	1.687546	-3.902341
C	-2.146850	2.077968	-2.537905
C	-1.023986	1.826801	-1.700220
C	0.121268	1.201714	-2.259300
C	0.125166	0.801019	-3.571874
H	-0.946215	0.740198	-5.439178
H	-2.958707	1.890823	-4.516738
C	-1.143100	2.191815	-0.324669
H	0.977333	1.000597	-1.636658
C	-2.333774	2.747330	0.080565
C	-3.373430	2.971578	-0.837703
H	-2.519718	2.987553	1.118961
H	-4.307563	3.407706	-0.494146
N	-3.297650	2.655598	-2.105020
O	1.131723	0.137892	-4.177343
C	2.235596	-0.269245	-3.383283
H	1.912956	-0.937713	-2.578087
H	2.912229	-0.802995	-4.045626
H	2.748328	0.597538	-2.957132
C	-0.050270	1.910676	0.696555
H	0.682021	1.230880	0.257797
N	-0.672707	1.214858	1.821739
H	-1.671663	1.046780	1.733718
C	0.745635	3.130252	1.242291
H	1.419958	2.694203	1.982169
C	-0.067620	4.242409	1.939975
C	2.705981	4.480185	0.959807
C	0.953783	4.659272	-0.673931
C	0.579938	5.594062	1.612459
H	-1.097062	4.275550	1.586887
H	-0.102845	4.060501	3.015568
C	2.096179	5.556980	1.899638
H	3.305868	3.755634	1.514531
H	3.355277	4.936244	0.209336
C	0.379403	5.859869	0.114951
H	0.171949	4.111424	-1.195376
H	1.677065	4.979010	-1.426585
H	0.116016	6.385140	2.204790
H	2.508465	6.536065	1.637345
H	-0.684704	5.981343	-0.102427
H	0.879818	6.788845	-0.171032
N	1.655734	3.726874	0.234959
C	2.412835	5.289626	3.343100
H	2.173627	4.294082	3.716235
C	2.961221	6.170999	4.169632
H	3.225609	7.170485	3.835327
H	3.167746	5.926162	5.205518
C	-0.054444	0.394164	2.674335
C	-0.212168	-0.937996	4.800093
C	1.843015	-0.970522	3.353567
C	1.003302	-1.738534	4.338681
C	1.307885	0.136610	2.673065
C	-0.969927	-0.310971	3.634794
H	0.127276	-0.138632	5.466456
H	0.703933	-2.694561	3.891425
H	1.966952	0.674609	2.005050
H	-1.538172	-1.049679	3.062307
H	-0.885774	-1.572900	5.379964
H	1.618356	-2.007833	5.202251
H	-1.706977	0.407749	4.002349
C	3.090953	-1.446681	2.942162
C	-1.331016	-4.291309	-0.265560
C	-2.871289	-3.940753	2.101982
C	-0.824726	-3.595914	0.852333
C	-2.653272	-4.734914	-0.152052
C	-3.421161	-4.552880	0.989608
C	-1.573641	-3.467114	2.016440
H	-3.118084	-5.238670	-0.986821
H	-4.449256	-4.894335	1.004071
H	-1.117025	-2.973920	2.862043
H	-3.446621	-3.811491	3.010767
C	-0.504729	-4.660307	-1.517252
C	0.152926	-3.450995	-2.208119
H	-0.565693	-2.647759	-2.383595
H	0.553897	-3.767640	-3.175116
H	0.989323	-3.046358	-1.641057
C	0.578793	-5.669299	-1.090677
H	0.119289	-6.574936	-0.684560
H	1.244354	-5.266880	-0.327114
H	1.185378	-5.950974	-1.956961
C	-1.373940	-5.347320	-2.584532
H	-2.171892	-4.690713	-2.942989
H	-1.819232	-6.277639	-2.223713
H	-0.743017	-5.601013	-3.439872
N	0.430800	-2.911718	0.835052
C	1.637547	-3.332897	1.366395
C	0.593824	-1.641857	0.218728
C	2.629443	-2.243286	1.058356
H	3.667752	-2.524847	0.963282
C	1.975448	-1.330031	0.240755
H	2.388274	-0.458721	-0.234316
O	-0.357027	-1.019704	-0.261302
O	1.827183	-4.380361	1.946126
H	3.713888	-0.703698	2.450829
C	3.825120	-2.542067	3.667255

H	4.697107	-2.863776	3.092615
H	4.181763	-2.194686	4.643258
H	3.194601	-3.419369	3.824353
N	-5.666071	0.648494	0.154241
C	-5.075188	-0.601271	-0.267935
C	-3.619001	-0.610141	0.179903
O	-3.256962	-0.026510	1.187224
H	-5.187325	1.161601	0.878210
H	-5.094226	-0.642886	-1.357194
C	-5.787070	-1.829183	0.279075
C	-7.105500	-4.073280	1.280495
C	-5.976153	-1.978540	1.651494
C	-6.254876	-2.811341	-0.586157
C	-6.914725	-3.930069	-0.088302
C	-6.632157	-3.095030	2.150518
H	-5.608810	-1.214802	2.328676
H	-6.113333	-2.694323	-1.655074
H	-7.283993	-4.686381	-0.771533
H	-6.777298	-3.202973	3.219528
H	-7.622597	-4.942925	1.669932
O	-2.861412	-1.299975	-0.620083
H	-1.891040	-1.269112	-0.356678
C	-6.501066	1.322502	-0.681720
O	-6.676095	2.575194	-0.231409
O	-7.001702	0.840708	-1.674197
C	-7.467852	3.546012	-0.986472
C	-7.289020	4.822601	-0.173337
H	-7.623928	4.671346	0.855286
H	-7.875382	5.630533	-0.616543
H	-6.238937	5.125193	-0.159130
C	-6.900031	3.723871	-2.392769
H	-7.366806	4.596385	-2.857228
H	-7.094282	2.848898	-3.010328
H	-5.819653	3.886230	-2.355927
C	-8.931106	3.116294	-1.004255
H	-9.296713	2.974544	0.015990
H	-9.055840	2.187763	-1.560080
H	-9.532528	3.894923	-1.480459
N	5.376473	-0.499570	-0.511089
C	5.141823	0.803790	-1.075898
C	4.007382	1.489698	-0.313273
O	3.882366	1.369855	0.890838
H	5.321076	-0.602097	0.490156
H	4.833153	0.655091	-2.110122
C	6.351881	1.730744	-1.056699
C	8.511469	3.498677	-1.016580
C	7.466751	1.457188	-0.272937
C	6.323682	2.896152	-1.822880
C	7.397620	3.775266	-1.803135
C	8.543329	2.337958	-0.254568
H	7.503265	0.546390	0.312528
H	5.455869	3.111428	-2.437250
H	7.368245	4.674598	-2.407792
H	9.409834	2.112811	0.356784
H	9.352001	4.182970	-1.002770
O	3.229578	2.200184	-1.084271
H	2.515357	2.756162	-0.527058
C	5.303860	-1.620691	-1.283799
O	5.388444	-2.707931	-0.506519
O	5.191532	-1.609835	-2.490754
C	5.248171	-4.059164	-1.062761
C	5.262828	-4.938511	0.182319
H	6.170698	-4.762434	0.763755
H	5.237475	-5.990020	-0.112148
H	4.392975	-4.741690	0.814157
C	3.918607	-4.202305	-1.797154
H	3.758264	-5.255356	-2.040492
H	3.901819	-3.618752	-2.715934
H	3.096171	-3.875532	-1.158329
C	6.442499	-4.361235	-1.960374
H	7.375317	-4.226264	-1.407225
H	6.447246	-3.709702	-2.833632
H	6.389173	-5.399664	-2.296662

42. TS7-syn

Imaginary Frequencies = 1 (Transition State)

Energy (Hartree) = -3797.74029922

C	1.212808	-4.155303	0.406150
C	2.442244	-4.058991	-2.152212
C	0.561501	-3.578988	-0.699658
C	2.554049	-4.513350	0.198894
C	3.167388	-4.465030	-1.040730
C	1.153563	-3.599911	-1.963917
H	3.143562	-4.860104	1.038187
H	4.202878	-4.768348	-1.134322
H	0.578975	-3.240019	-2.805825
H	2.881808	-4.065672	-3.142542
C	0.589806	-4.509411	1.777414
C	1.087154	-3.556370	2.875882
H	2.178660	-3.510208	2.893543
H	0.741037	-3.911122	3.852071
H	0.704865	-2.547313	2.721981
C	-0.948412	-4.533501	1.783689
H	-1.345071	-5.111788	0.944329
H	-1.396782	-3.541362	1.769802
H	-1.284261	-5.015946	2.705574
C	1.033130	-5.945866	2.141539
H	2.107907	-6.025571	2.312446
H	0.757723	-6.653128	1.354117
H	0.535354	-6.252461	3.065457
N	-0.688706	-2.901447	-0.598261
C	-1.883725	-3.291766	-1.183249
C	-0.910690	-1.772540	0.239724
C	-2.915734	-2.284818	-0.737977
H	-3.941594	-2.626444	-0.671962
C	-2.291228	-1.486130	0.222195
H	-2.720255	-0.725137	0.849286
O	0.001228	-1.224164	0.872894

O	-2.029180	-4.252676	-1.905218	H	-1.163640	-2.493617	-3.651443
C	4.754049	2.730240	1.677366	H	-2.201163	0.763815	-1.368699
C	3.998649	2.426666	2.766683	H	1.125692	-1.222634	-2.429969
C	2.611872	2.138214	2.647806	H	0.623103	-1.350277	-4.848377
C	1.999043	2.193953	1.366001	H	-1.945620	-1.491024	-4.835229
C	2.807279	2.536465	0.246460	H	1.580685	0.285920	-3.168276
C	4.149709	2.773507	0.395972	C	-3.370092	-1.272676	-2.447936
H	5.818995	2.910434	1.751830	H	-1.123311	1.266531	0.290786
H	4.433532	2.372828	3.757177	C	-4.143178	-2.241568	-3.302276
C	0.608194	1.875778	1.297018	H	-4.443684	-1.774513	-4.246642
H	2.363806	2.602798	-0.735016	H	-3.562483	-3.137913	-3.529763
C	-0.030831	1.539707	2.464118	H	-5.051759	-2.546027	-2.782893
C	0.675056	1.528877	3.681060	H	-3.973881	-0.577987	-1.872382
H	-1.082639	1.274863	2.453493	N	5.553667	-0.239507	-0.335972
H	0.155764	1.261276	4.597088	C	4.774537	-0.757009	0.754014
N	1.949092	1.812392	3.785837	C	3.311020	-0.775603	0.317178
O	4.993683	3.046467	-0.622763	O	2.966632	-0.479034	-0.813395
C	4.527930	2.841535	-1.942353	H	5.133429	-0.235644	-1.251519
H	3.780641	3.591707	-2.223610	H	4.837730	-0.064243	1.598952
H	5.399350	2.937826	-2.586286	C	5.206550	-2.137540	1.238444
H	4.101625	1.838710	-2.050055	C	5.909167	-4.691122	2.121830
C	-0.219776	1.822040	0.021376	C	5.941812	-2.985383	0.419547
N	0.458887	1.068451	-1.026400	C	4.838130	-2.568849	2.511108
H	1.428032	0.797560	-0.885381	C	5.183352	-3.839651	2.949476
C	-0.685660	3.182843	-0.590210	C	6.292887	-4.257396	0.859206
H	-0.843586	2.968645	-1.648853	H	6.241832	-2.648260	-0.565943
C	0.244294	4.402597	-0.448258	H	4.266274	-1.910166	3.154687
C	-2.651825	4.526691	-1.106757	H	4.887683	-4.165018	3.940551
C	-1.999228	4.257623	1.232064	H	6.866759	-4.910480	0.211137
C	-0.605659	5.669234	-0.282607	H	6.181444	-5.682968	2.464357
H	0.884329	4.306210	0.428788	O	2.511383	-1.113410	1.283586
H	0.899185	4.468423	-1.318307	H	1.551343	-1.198716	0.993699
C	-1.714147	5.733091	-1.355039	C	6.718757	0.426018	-0.130683
H	-2.834760	3.946641	-2.012316	O	7.200222	0.861667	-1.304575
H	-3.617796	4.830598	-0.704002	O	7.230432	0.578154	0.957995
C	-1.273856	5.608505	1.097130	C	8.459654	1.598018	-1.377141
H	-1.500445	3.572865	1.911492	C	8.571112	1.923517	-2.861902
H	-3.029804	4.353910	1.570503	H	8.549344	1.008001	-3.457170
H	0.026820	6.554261	-0.360985	H	9.509786	2.446191	-3.058808
H	-2.282603	6.652584	-1.192173	H	7.745677	2.565819	-3.178112
H	-0.522289	5.703125	1.882858	C	8.383914	2.880776	-0.553485
H	-1.975496	6.438099	1.211683	H	9.253245	3.503540	-0.780371
N	-2.045776	3.598217	-0.106740	H	8.375231	2.661235	0.512932
C	-1.176146	5.760367	-2.758502	H	7.476875	3.434929	-0.802877
H	-0.692963	4.852348	-3.117211	C	9.609890	0.696719	-0.937920
C	-1.254958	6.809082	-3.566689	H	9.617455	-0.222440	-1.529498
H	-1.736307	7.730802	-3.252262	H	9.522664	0.440402	0.117189
H	-0.843446	6.784084	-4.569256	H	10.558883	1.215292	-1.096481
C	-0.173229	0.372818	-1.978203	N	-5.772488	-0.080198	0.037484
C	-0.018910	-0.726870	-4.221936	C	-5.229579	0.384682	1.287872
C	-2.122257	-0.770151	-2.844168	C	-4.010264	1.289858	1.013109
C	-1.332093	-1.446355	-3.929345	O	-3.914966	1.771290	-0.157619
C	-1.551445	0.249442	-2.064943	H	-5.607772	0.491095	-0.776418
C	0.719927	-0.348250	-2.943935	H	-4.877838	-0.476755	1.859548
H	-0.232085	0.185175	-4.789104	C	-6.225059	1.154742	2.147011

C	-7.985929	2.620715	3.748072
C	-7.359002	1.740368	1.593736
C	-5.977484	1.310780	3.510172
C	-6.851688	2.038542	4.305820
C	-8.236565	2.468772	2.390308
H	-7.564485	1.616697	0.536425
H	-5.089045	0.863877	3.941423
H	-6.650656	2.147118	5.365857
H	-9.120084	2.914971	1.947215
H	-8.671887	3.184437	4.370298
O	-3.221635	1.490823	1.943499
C	-6.552129	-1.184598	-0.038333
O	-6.950305	-1.379706	-1.310412
O	-6.825632	-1.891420	0.908583
C	-7.946259	-2.398545	-1.636837
C	-8.141230	-2.212351	-3.137097
H	-8.482561	-1.197752	-3.354513
H	-8.889543	-2.918698	-3.503679
H	-7.206661	-2.388079	-3.674122
C	-7.413069	-3.796200	-1.332383
H	-8.128957	-4.539085	-1.693814
H	-7.268841	-3.935303	-0.262653
H	-6.462694	-3.967768	-1.843929
C	-9.246162	-2.108829	-0.890634
H	-9.574828	-1.085432	-1.090087
H	-9.120136	-2.238999	0.183371
H	-10.023861	-2.793006	-1.239043
H	-2.725630	2.761333	-0.068974

43. TS9-anti

Imaginary Frequencies = 1 (Transition State)

Energy (Hartree) = -3876.38325591

C	4.233290	5.915580	-2.359496
C	3.070362	6.385970	-2.878590
C	1.857765	5.659961	-2.730140
C	1.875136	4.424594	-2.023252
C	3.099862	3.963507	-1.458717
C	4.253892	4.690564	-1.639013
H	5.166472	6.454058	-2.471553
H	3.029476	7.320537	-3.424495
C	0.641195	3.714799	-1.946891
H	3.133055	3.050065	-0.876955
C	-0.463828	4.267381	-2.548731
C	-0.370633	5.511604	-3.194879
H	-1.415160	3.748571	-2.555075
H	-1.253880	5.947172	-3.653891
N	0.744219	6.192675	-3.291979
O	5.470046	4.344291	-1.182392
C	5.578216	3.178425	-0.378296
H	5.233015	2.290760	-0.917506
H	6.636228	3.076265	-0.145817
H	5.001736	3.289719	0.543071
C	0.528690	2.348563	-1.290487
H	1.524456	1.935009	-1.150902
N	-0.190916	1.435693	-2.166388
H	-1.208324	1.420839	-2.110868
C	-0.131939	2.271364	0.107312
H	-0.297845	1.201711	0.248071
C	-1.438162	3.035955	0.361981
C	0.408531	1.936633	2.447889
C	0.902123	4.113674	1.445722
C	-1.472615	3.449940	1.842253
H	-1.497927	3.939396	-0.248247
H	-2.288941	2.409261	0.093180
C	-1.071995	2.269403	2.756608
H	0.566584	0.870515	2.285920
H	1.085813	2.261993	3.236023
C	-0.441749	4.571229	2.041056
H	1.118127	4.597153	0.494991
H	1.754601	4.266325	2.105813
H	-2.470513	3.799705	2.107017
H	-1.132300	2.613743	3.792753
H	-0.773477	5.483203	1.541848
H	-0.334305	4.797846	3.104377
N	0.831950	2.642626	1.196095
C	-1.960205	1.065264	2.611887
H	-1.974955	0.576533	1.641160
C	-2.693367	0.555357	3.592686
H	-2.696272	0.993581	4.587160
H	-3.312701	-0.320000	3.433711
C	0.359802	0.440785	-2.858427
C	0.133298	-1.124072	-4.782535
C	2.175306	-1.116693	-3.294003
C	1.342273	-1.904881	-4.272989
C	1.709640	0.114541	-2.820926
C	-0.593096	-0.396483	-3.657253
H	-0.558086	-1.795396	-5.297563
H	1.976737	-2.193752	-5.117187
H	2.379695	0.724364	-2.236448
H	-1.388907	0.238331	-4.051699
H	0.465768	-0.384279	-5.517200
H	1.032602	-2.850456	-3.815143
H	-1.075785	-1.096296	-2.968074
C	3.319448	-1.694053	-2.705304
C	-1.876510	-3.876360	-0.050555
C	-2.918056	-3.935341	-2.693966
C	-1.122694	-3.422338	-1.146474
C	-3.199084	-4.248391	-0.335884
C	-3.724914	-4.267160	-1.615264
C	-1.628468	-3.507771	-2.445390
H	-3.849959	-4.534950	0.479692
H	-4.760597	-4.545063	-1.771016
H	-0.988728	-3.216431	-3.267185
H	-3.297742	-3.977388	-3.707370
C	-1.408651	-4.005622	1.416615
C	0.118653	-4.026232	1.602235
H	0.593602	-4.747415	0.931656
H	0.344711	-4.332628	2.627376

H	0.583394	-3.052289	1.457717
C	-2.006263	-2.856121	2.246494
H	-3.093948	-2.817136	2.139131
H	-1.596682	-1.894179	1.934573
H	-1.778441	-3.001425	3.307502
C	-1.916025	-5.348971	1.986915
H	-1.570457	-6.187816	1.376532
H	-3.003478	-5.401334	2.055249
H	-1.524753	-5.480224	2.999185
N	0.147723	-2.785019	-1.008899
C	0.326589	-1.521199	-0.398419
C	1.390320	-3.345676	-1.280024
C	1.704892	-1.280091	-0.265889
H	2.102313	-0.399099	0.213305
C	2.407101	-2.308563	-0.898613
H	3.384077	-2.650771	-0.585679
O	1.568942	-4.458052	-1.723406
O	-0.641783	-0.829390	-0.025970
N	-5.631497	0.174609	0.295692
C	-5.166260	0.167340	-1.070231
C	-3.639865	0.112737	-1.113906
O	-3.162324	-0.792156	-0.303181
H	-5.376679	-0.608040	0.878847
H	-5.462107	1.118118	-1.513444
C	-5.731190	-0.982690	-1.894933
C	-6.731691	-3.064303	-3.468518
C	-6.683816	-1.855765	-1.382093
C	-5.286052	-1.156574	-3.205925
C	-5.782537	-2.189606	-3.987307
C	-7.181238	-2.892112	-2.166209
H	-7.052451	-1.720291	-0.372787
H	-4.545731	-0.477997	-3.616996
H	-5.428628	-2.311209	-5.004772
H	-7.925069	-3.564776	-1.754384
H	-7.120163	-3.872041	-4.078058
O	-2.984179	0.841771	-1.835708
C	-5.844521	1.363520	0.940455
O	-6.003811	1.136743	2.247429
O	-5.897059	2.439065	0.384550
C	-6.336211	2.220530	3.174844
C	-6.384991	1.503755	4.518168
H	-7.136777	0.711596	4.502923
H	-6.641522	2.213201	5.308032
H	-5.413607	1.060701	4.748601
C	-5.240535	3.282562	3.176232
H	-5.443643	4.003553	3.972215
H	-5.201720	3.809531	2.223948
H	-4.271793	2.816737	3.368822
C	-7.701426	2.799697	2.818453
H	-8.451485	2.005561	2.783356
H	-7.672696	3.306202	1.854300
H	-7.999737	3.519841	3.584369
H	-2.155692	-0.857843	-0.305235
H	1.787966	2.269221	0.948780
C	3.888174	-2.958307	-3.309447
H	3.132421	-3.729777	-3.458971
H	4.659085	-3.375644	-2.659266
N	4.532838	-0.725344	1.707940
H	4.190330	-0.608353	0.767533
C	4.167558	0.294285	2.661755
C	3.656589	1.550588	1.916277
O	3.690221	2.628378	2.513230
O	3.141624	1.350720	0.768247
C	3.031378	-0.106177	3.598974
C	2.124503	-1.106820	3.262244
C	1.023263	-1.360600	4.071587
C	0.815110	-0.617637	5.226977
C	1.729136	0.370363	5.578721
C	2.831385	0.620563	4.771263
C	4.996152	-1.935635	2.103473
O	5.304068	-2.207472	3.243252
O	5.066844	-2.775626	1.047313
C	5.266226	-4.210804	1.253969
C	5.195934	-4.785566	-0.156277
C	6.640187	-4.477842	1.862114
C	4.127377	-4.762094	2.108097
H	6.707496	-4.078663	2.871892
H	6.817292	-5.556125	1.890985
H	7.417612	-4.019545	1.245162
H	5.930659	-4.298191	-0.802109
H	5.420115	-5.854208	-0.126220
H	4.201167	-4.663915	-0.591043
H	4.170637	-4.371327	3.124203
H	3.164001	-4.496070	1.663329
H	4.196186	-5.852052	2.146390
H	5.035666	0.580342	3.257946
H	3.530821	1.406455	5.033097
H	1.584344	0.947985	6.485065
H	-0.048764	-0.812282	5.852347
H	0.321630	-2.136091	3.790000
H	2.263482	-1.677630	2.351059
C	4.369891	-0.795420	-2.062548
H	3.915647	-0.103995	-1.347098
H	5.051627	-1.426653	-1.483240
C	5.171553	0.007283	-3.090208
H	5.948785	0.596889	-2.597241
H	5.660864	-0.649596	-3.814128
H	4.524395	0.694548	-3.642139
H	4.353548	-2.745431	-4.278828

44. Z-dienamine_anti-open_external_OMe
Imaginary Frequencies = 0 (Equilibrium Geometry)
Energy (Hartree) = -1327.4039387899998

C	-2.454244	3.686828	0.192901
C	-2.256723	3.330630	1.498743
C	-1.478219	2.203961	1.849726
C	-0.879007	1.433685	0.814022
C	-1.088792	1.824698	-0.527793

C	-1.862414	2.918572	-0.838324
H	-3.063044	4.550691	-0.038004
H	-2.701203	3.901479	2.305103
C	-0.137180	0.277477	1.205380
H	-0.665496	1.268032	-1.353035
C	-0.052819	-0.004404	2.541542
C	-0.667906	0.841336	3.487715
H	0.467183	-0.895153	2.874093
H	-0.583746	0.610193	4.546704
N	-1.356545	1.908033	3.173210
O	-2.012178	3.192420	-2.156217
C	-2.830224	4.283729	-2.535473
H	-2.434854	5.232415	-2.157902
H	-2.817812	4.299691	-3.623354
H	-3.860488	4.151041	-2.189511
C	0.544180	-0.650044	0.208608
H	0.203556	-0.397490	-0.803309
N	0.248709	-2.038716	0.485579
H	1.049568	-2.626731	0.282758
C	2.067880	-0.452203	0.275982
H	2.363799	-0.621319	1.316727
C	2.511391	0.966693	-0.162488
C	4.187955	-1.437104	-0.173692
C	2.594096	-1.278821	-1.944209
C	3.688734	0.815363	-1.134491
H	1.700081	1.495225	-0.671690
H	2.780165	1.572767	0.705123
C	4.812125	-0.042027	-0.505656
H	4.302752	-1.665242	0.889905
H	4.697420	-2.227353	-0.730437
C	3.176666	0.090766	-2.386374
H	1.532565	-1.358406	-2.187940
H	3.094249	-2.104149	-2.456251
H	4.086978	1.797275	-1.399457
H	5.594301	-0.165287	-1.262069
H	2.411682	0.699060	-2.877262
H	3.992876	-0.044735	-3.101737
N	2.762844	-1.491074	-0.501045
C	5.430423	0.598026	0.701439
H	4.785147	0.706349	1.573190
C	6.686684	1.020347	0.777525
H	7.366709	0.924119	-0.064616
H	7.080221	1.474992	1.680022
C	-0.978792	-2.576423	0.138968
C	-2.470219	-4.594330	0.313952
C	-3.352108	-2.409551	-0.539064
C	-3.368606	-3.910677	-0.717091
C	-2.054610	-1.833369	-0.198023
C	-1.035966	-4.081875	0.200649
H	-2.489989	-5.679358	0.180982
H	-4.391711	-4.289465	-0.652554
H	-1.973284	-0.756378	-0.212206
H	-0.436938	-4.425382	1.049797
H	-2.850502	-4.382656	1.319153
H	-2.995313	-4.154789	-1.720986
H	-0.561773	-4.489449	-0.702235
C	-4.461416	-1.676408	-0.718134
H	-5.373348	-2.204214	-0.991676
C	-4.586864	-0.183684	-0.602090
H	-4.651326	0.289467	-1.589600
H	-3.749021	0.276465	-0.073944
H	-5.501180	0.084089	-0.063737

45. Z-dienamine_anti-open_internal_OMe
Imaginary Frequencies = 0 (Equilibrium Geometry)
Energy (Hartree) = -1327.4069107

C	-2.208396	3.938125	-0.056592
C	-2.072738	3.689154	1.273430
C	-1.394705	2.529529	1.739773
C	-0.837410	1.632064	0.790627
C	-0.984279	1.918406	-0.595686
C	-1.663585	3.036450	-1.008021
H	-2.733055	4.812751	-0.421628
H	-2.485093	4.359535	2.017750
C	-0.196682	0.464136	1.294177
H	-0.576451	1.237709	-1.327128
C	-0.167934	0.285241	2.653683
C	-0.737277	1.247491	3.506140
H	0.275760	-0.610472	3.072235
H	-0.700565	1.101135	4.582617
N	-1.330180	2.337397	3.081854
O	-1.880159	3.375058	-2.297839
C	-1.409995	2.495061	-3.301312
H	-1.864156	1.503253	-3.201524
H	-1.706478	2.935819	-4.250674
H	-0.318740	2.402561	-3.269144
C	0.445190	-0.587149	0.398741
H	0.165620	-0.388117	-0.643211
N	0.024284	-1.924103	0.757029
H	0.788716	-2.584105	0.667208
C	1.976829	-0.506438	0.510794
H	2.227083	-0.616155	1.570778
C	2.548546	0.834657	-0.022604
C	4.038257	-1.628650	0.112266
C	2.392185	-1.607801	-1.615233
C	3.598858	0.514505	-1.093698
H	1.763846	1.451708	-0.469286
H	2.976985	1.422236	0.791970
C	4.725126	-0.370166	-0.509625
H	4.174770	-1.642203	1.197665
H	4.487131	-2.543931	-0.281198
C	2.905307	-0.271605	-2.214552
H	1.332286	-1.763852	-1.828387
H	2.924565	-2.459908	-2.044450
H	4.028214	1.438773	-1.486712
H	5.363067	-0.681509	-1.343915
H	2.077097	0.320257	-2.616800
H	3.601526	-0.455869	-3.037250

N	2.601465	-1.657391	-0.162157
C	5.584237	0.348033	0.486698
H	5.089159	0.678470	1.399693
C	6.881162	0.589056	0.336996
H	7.416058	0.269656	-0.553330
H	7.453607	1.111459	1.095576
C	-1.210317	-2.398277	0.348363
C	-2.853275	-4.294754	0.513151
C	-3.501772	-2.114515	-0.540385
C	-3.611424	-3.620007	-0.630129
C	-2.199878	-1.605996	-0.117385
C	-1.381216	-3.889171	0.492839
H	-2.941489	-5.382209	0.443227
H	-4.661058	-3.924196	-0.632951
H	-2.042184	-0.538845	-0.174976
H	-0.879408	-4.216527	1.408780
H	-3.297655	-3.992790	1.467660
H	-3.175209	-3.953286	-1.581707
H	-0.865930	-4.385315	-0.340576
C	-4.536674	-1.324893	-0.865724
H	-5.456555	-1.811562	-1.185463
C	-4.573610	0.176390	-0.854601
H	-4.612452	0.580409	-1.873541
H	-3.712027	0.625038	-0.356468
H	-5.471860	0.535054	-0.342006

46. Z-dienamine_syn-open_external_OMe
Imaginary Frequencies = 0 (Equilibrium Geometry)
Energy (Hartree) = -1327.40223528

C	0.582205	4.359020	0.041594
C	0.795551	3.980670	1.339188
C	0.703269	2.628843	1.739576
C	0.380433	1.643261	0.764296
C	0.171894	2.053708	-0.571103
C	0.266934	3.378512	-0.928821
H	0.661825	5.403623	-0.228492
H	1.042749	4.711534	2.099647
C	0.282068	0.287896	1.212542
H	-0.032470	1.324438	-1.341302
C	0.521878	0.040745	2.537725
C	0.841812	1.095185	3.420355
H	0.474019	-0.972262	2.923144
H	1.028979	0.877338	4.468919
N	0.929323	2.345914	3.053935
O	0.048864	3.663234	-2.236463
C	0.154294	5.007176	-2.665859
H	1.160603	5.405132	-2.498006
H	-0.050622	4.994663	-3.734518
H	-0.580821	5.647827	-2.167301
C	-0.142882	-0.867193	0.318137
H	0.046099	-1.787637	0.894862
N	0.539896	-0.932106	-0.951401
H	-0.077899	-1.286256	-1.673072
C	-1.659421	-0.770904	0.040980
H	-1.843767	0.218340	-0.388221
C	-2.522966	-0.940410	1.313205
C	-3.395818	-1.352160	-1.478418
C	-2.111335	-3.096699	-0.468875
C	-3.722359	-1.827926	0.951741
H	-1.952799	-1.424527	2.112859
H	-2.838173	0.031266	1.698773
C	-4.436580	-1.293628	-0.312325
H	-3.321145	-0.381519	-1.976658
H	-3.704864	-2.080569	-2.232064
C	-3.189896	-3.232555	0.638825
H	-1.119416	-3.354160	-0.090974
H	-2.313697	-3.771078	-1.304127
H	-4.431130	-1.863182	1.781878
H	-5.267233	-1.971664	-0.534004
H	-2.765469	-3.680589	1.541464
H	-4.009605	-3.877973	0.309799
N	-2.064183	-1.728511	-1.000701
C	-4.999368	0.084678	-0.126012
H	-4.275543	0.888881	0.005600
C	-6.293376	0.380834	-0.122199
H	-7.049078	-0.388133	-0.258276
H	-6.643494	1.397928	0.015577
C	1.869679	-1.278646	-1.069205
C	3.643719	-2.224783	-2.579985
C	4.150523	-1.654390	-0.191648
C	4.658447	-1.613098	-1.615795
C	2.722523	-1.391905	-0.027304
C	2.318614	-1.473353	-2.495775
H	3.484342	-3.276613	-2.318448
H	4.823712	-0.566000	-1.903434
H	2.346537	-1.252353	0.976489
H	2.412002	-0.487462	-2.968144
H	4.022576	-2.198622	-3.605186
H	5.622203	-2.123092	-1.688507
H	1.539252	-2.007848	-3.050543
C	4.994065	-1.890778	0.824796
H	6.038898	-2.068232	0.575577
C	4.673035	-1.926695	2.289818
H	4.980486	-2.880575	2.732232
H	3.610049	-1.794660	2.497962
H	5.215103	-1.141362	2.828647

47. Z-dienamine_syn-open_internal_OMe
Imaginary Frequencies = 0 (Equilibrium Geometry)
Energy (Hartree) = -1327.40569485

C	-0.577953	4.401587	0.323537
C	-0.781740	4.154851	-0.998862
C	-0.696594	2.835995	-1.521506
C	-0.390066	1.766284	-0.638531
C	-0.191885	2.045670	0.741498
C	-0.282885	3.332126	1.208108

H	-0.642114	5.403172	0.731624
H	-1.015049	4.953369	-1.692703
C	-0.293121	0.460046	-1.202948
H	-0.010910	1.219308	1.410362
C	-0.525498	0.331270	-2.549403
C	-0.832257	1.459562	-3.333296
H	-0.478232	-0.643862	-3.022490
H	-1.013761	1.340733	-4.398450
N	-0.913109	2.675294	-2.853716
O	-0.115823	3.694216	2.500814
C	0.168775	2.673916	3.438347
H	1.106600	2.162067	3.195640
H	0.265196	3.169052	4.402387
H	-0.642576	1.939410	3.484639
C	0.128546	-0.771961	-0.414971
H	-0.066457	-1.637651	-1.069050
N	-0.546018	-0.946543	0.849661
H	0.070542	-1.388182	1.522929
C	1.646223	-0.703240	-0.135998
H	1.831373	0.243231	0.380923
C	2.507134	-0.755192	-1.419393
C	3.383847	-1.413126	1.325600
C	2.110901	-3.063886	0.155726
C	3.715396	-1.659395	-1.137430
H	1.938953	-1.174297	-2.256293
H	2.812295	0.248843	-1.720863
C	4.423328	-1.233563	0.170755
H	3.302529	-0.495790	1.915743
H	3.698846	-2.208905	2.005027
C	3.197185	-3.091556	-0.952087
H	1.122649	-3.287185	-0.252351
H	2.309563	-3.813496	0.925128
H	4.425006	-1.612798	-1.966207
H	5.262927	-1.917925	0.330371
H	2.782969	-3.464172	-1.892875
H	4.022179	-3.754299	-0.674762
N	2.054741	-1.751364	0.813205
C	4.967912	0.163383	0.111936
H	4.234151	0.966953	0.049411
C	6.257849	0.475479	0.140161
H	7.023321	-0.292724	0.209505
H	6.594719	1.505307	0.095672
C	-1.883090	-1.276035	0.946902
C	-3.667244	-2.314040	2.383521
C	-4.177594	-1.520824	0.060041
C	-4.674660	-1.597049	1.486600
C	-2.744845	-1.277131	-0.092907
C	-2.326244	-1.586932	2.354522
H	-3.532733	-3.341461	2.028016
H	-4.815553	-0.576754	1.868202
H	-2.372140	-1.058872	-1.083849
H	-2.395316	-0.644826	2.913262
H	-4.038371	-2.371374	3.410254
H	-5.648696	-2.091142	1.521692
H	-1.555249	-2.185065	2.853380
C	-5.032927	-1.644511	-0.966301
H	-6.079571	-1.822094	-0.724919
C	-4.723697	-1.553514	-2.431329
H	-5.025431	-2.469725	-2.950802
H	-3.664214	-1.392095	-2.636290
H	-5.278947	-0.731460	-2.896557

48. Z-iPr-EtMe-Dienamine
Imaginary Frequencies = 0 (Equilibrium Geometry)
Energy (Hartree) = -563.954924243

C	1.684174	1.861701	0.425274
C	1.445316	0.442816	-0.052100
C	0.037378	0.070430	-0.213401
C	-0.993657	0.931869	-0.064291
C	-0.765918	2.389937	0.241836
C	0.642867	2.826995	-0.139006
N	-2.323607	0.590145	-0.207816
C	-2.803580	-0.783933	-0.208372
C	-4.251854	-0.795134	-0.684898
C	-2.655050	-1.462594	1.156584
C	2.446974	-0.434449	-0.264513
C	2.200888	-1.852940	-0.735389
H	1.631902	1.888241	1.522583
H	2.680148	2.207273	0.146770
H	-0.185049	-0.966451	-0.427329
H	-0.935569	2.558180	1.313710
H	-1.510667	2.989073	-0.292691
H	0.831911	3.841015	0.223801
H	0.729280	2.851496	-1.230729
H	-2.970854	1.253581	0.192024
H	-2.203729	-1.333030	-0.941945
H	-4.890125	-0.238594	0.010593
H	-4.338416	-0.337552	-1.672658
H	-4.630772	-1.818097	-0.737773
H	-2.951474	-2.513671	1.101845
H	-1.621930	-1.414625	1.507125
H	-3.292289	-0.964891	1.894716
C	3.902337	-0.135662	-0.008035
C	1.937832	-2.824963	0.421397
H	1.368349	-1.894277	-1.443115
H	3.082574	-2.193867	-1.289864
H	4.355794	-0.953036	0.564917
H	4.462624	-0.070040	-0.949232
H	4.073454	0.783436	0.551449
H	2.766047	-2.814674	1.136177
H	1.030072	-2.544233	0.962837
H	1.817731	-3.850705	0.060986

49. Z-iPr-HMe-Dienamine
Imaginary Frequencies = 0 (Equilibrium Geometry)
Energy (Hartree) = -485.329276201

C	2.490568	-1.164174	-0.291215
C	1.932927	0.237578	-0.187496
C	0.474522	0.331674	-0.175654
C	-0.333926	-0.749716	-0.078059
C	0.220452	-2.143871	0.093445
C	1.673716	-2.136948	0.556595
N	-1.706270	-0.711085	-0.187080
C	-2.485624	0.516896	-0.206161
C	-2.533247	1.204404	1.161852
C	-3.885757	0.201693	-0.721979
C	2.760854	1.292659	-0.132223
C	2.387784	2.743372	-0.047266
H	2.445055	-1.491589	-1.338747
H	3.542015	-1.176140	0.006021
H	0.030354	1.314690	-0.266921
H	0.132618	-2.672038	-0.864248
H	-0.402927	-2.694158	0.807522
H	2.088412	-3.146629	0.494333
H	1.721917	-1.829086	1.606725
H	-2.187362	-1.505365	0.207470
H	-2.003631	1.189189	-0.923749
H	-3.060962	0.570666	1.881847
H	-1.527132	1.390282	1.543241
H	-3.059825	2.160607	1.097947
H	-4.485547	1.112332	-0.784413
H	-3.840220	-0.256757	-1.712039
H	-4.399019	-0.489737	-0.044354
H	3.829971	1.089873	-0.161357
H	1.320057	2.899505	0.114903
H	2.919417	3.231447	0.776433
H	2.667086	3.279101	-0.962081
